# Phase 1 Study in Malaria Naïve Adults of BSAM2/Alhydrogel®+CPG 7909, a Blood Stage Vaccine against *P. falciparum* Malaria

**DOI:** 10.1371/journal.pone.0046094

**Published:** 2012-10-04

**Authors:** Ruth D. Ellis, Yimin Wu, Laura B. Martin, Donna Shaffer, Kazutoyo Miura, Joan Aebig, Andrew Orcutt, Kelly Rausch, Daming Zhu, Anders Mogensen, Michael P. Fay, David L. Narum, Carole Long, Louis Miller, Anna P. Durbin

**Affiliations:** 1 Laboratory of Malaria Immunology and Vaccinology, National Institute of Allergy and Infectious Diseases, National Institutes of Health (NIAID/NIH), Rockville, Maryland, United States of America; 2 Center for Immunization Research, Johns Hopkins Bloomberg School of Public Health, Baltimore, Maryland, United States of America; 3 Biostatistics Research Branch, NIAID/NIH, Rockville, Maryland, United States of America; 4 Laboratory of Malaria and Vector Research, NIAID/NIH, Rockville, Maryland, United States of America; Naval Medical Research Center, United States of America

## Abstract

A Phase 1 dose escalating study was conducted in malaria naïve adults to assess the safety, reactogenicity, and immunogenicity of the blood stage malaria vaccine BSAM2/Alhydrogel®+ CPG 7909. BSAM2 is a combination of the FVO and 3D7 alleles of recombinant AMA1 and MSP1_42_, with equal amounts by weight of each of the four proteins mixed, bound to Alhydrogel®, and administered with the adjuvant CPG 7909. Thirty (30) volunteers were enrolled in two dose groups, with 15 volunteers receiving up to three doses of 40 µg total protein at Days 0, 56, and 180, and 15 volunteers receiving up to three doses of 160 µg protein on the same schedule. Most related adverse events were mild or moderate, but 4 volunteers experienced severe systemic reactions and two were withdrawn from vaccinations due to adverse events. Geometric mean antibody levels after two vaccinations with the high dose formulation were 136 µg/ml for AMA1 and 78 µg/ml for MSP1_42_. Antibody responses were not significantly different in the high dose versus low dose groups and did not further increase after third vaccination. *In vitro* growth inhibition was demonstrated and was closely correlated with anti-AMA1 antibody responses. A Phase 1b trial in malaria-exposed adults is being conducted.

**Trial Registration:**

Clinicaltrials.gov NCT00889616

## Background

While significant advances have been made against malaria, the World Health Organization estimates that the *Plasmodium falciparum* parasite was responsible for an estimated 216 million illnesses and 655,000 deaths in 2010, mostly in African children [1]. Others have estimated as many as 1.2 million deaths were due to malaria in 2010 [2]. A partially effective vaccine may be licensed within the next 5 years [3], but a more highly protective second generation vaccine is needed, particularly in the face of growing parasite and mosquito resistance to interventional tools.

Over time, people living in endemic areas develop partially protective immunity against *P. falciparum* disease as a result of repeated natural infection. This acquired immunity is thought to be mediated in part by blood-stage (merozoite)-specific antibodies, which have been shown to be protective in passive transfer experiments [4]. Non-human primate models have also shown that protection against disease can be induced by vaccination with recombinant merozoite proteins and that protection was associated with high antibody titers [5], [6]. Merozoite Surface Protein 1 (MSP1) and Apical Membrane Antigen 1 (AMA1) are the blood stage antigens which have been most widely tested in clinical trials, including field trials in target populations [ as reviewed in 7] [and 8]. Strain-specific protection against clinical malaria in Malian children was recently reported with a highly immunogenic recombinant AMA1 vaccine, although overall protection against malaria was low [9].

The slow acquisition of protective immunity by natural infection and the limited allele-specific protection induced by experimental vaccination indicate that a single component malaria vaccine is unlikely to be sufficiently effective to protect clinical malaria. A multicomponent vaccine may provide greater protection by broadening antibody responses in a given individual and also by broadening coverage against antigenic diversity in parasites. In addition, there may be additive or synergistic consequences of targeting two merozoite surface proteins simultaneously, as observed by passive immunization in a rodent model [10]. Previous trials have shown safety and marked enhancement of the antibody responses to AMA1-C1 and MSP1_42_-C1when the adjuvant CPG 7909 was added to an Alhydrogel® formulation [11]–[13]. The blood stage vaccine candidate Blood Stage Antigen Mixture (BSAM) 2, containing two recombinant allelic proteins each of AMA1 and MSP1_42_, formulated on Alhydrogel® with CPG 7909, was therefore evaluated for safety and immunogenicity in malaria-naïve healthy adults, prior to a study in malaria-exposed adults.

## Methods

The protocol for this trial and supporting CONSORT checklist are available as supporting information; see Checklist S1 and Protocol S1.

### Participants

Participants were healthy adults age 18–50 enrolled at the Center for Immunization Research in Washington, DC. Exclusion criteria included prior malaria infection, recent or planned travel to a malaria-endemic area, recent use of malaria prophylaxis, and pre-existing autoimmune disease. Participants were required to be in good general health, without known significant medical conditions or significant medical history, and were required to have normal results for screening laboratories: complete blood count, alanine aminotransferase (ALT), and creatinine; no serologic evidence of hepatitis B, hepatitis C, or human immunodeficiency virus infection; and negative anti-double stranded DNA (dsDNA) as a marker for autoimmune disease. Serum pregnancy testing was performed at screening and urine pregnancy testing was performed prior to each vaccination for females.

### Ethics

The study protocol and consent documents were reviewed and approved by the Institutional Review Board (IRB) of the National Institute of Allergy and Infectious Diseases (NIAID) and the Western IRB. The study protocol was also reviewed and approved by the FMPOS Ethics Committee in Bamako, Mali, since the protocol combined Phase 1 trials; one in malaria-naïve adults in the U.S. and the other in malaria-exposed adults in Mali, the results of which are being published separately. Individual written informed consent was obtained from all participants prior to any study procedures.

### Interventions

BSAM2 is a combination of the FVO and 3D7 alleles of AMA1 and MSP1_42_, with equal amounts by weight of each of the four recombinant proteins mixed, bound to Alhydrogel®, and administered by mixing with CPG 7909 at the time of injection. The AMA1 and MSP1_42_ combination vaccines (AMA1-C1/Alhydrogel®+/−CPG 7909 and MSP1_42_-C1/Alhydrogel®+/−CPG 7909) have been previously evaluated in Phase 1 trials, and production and characterization of the recombinant proteins and the formulated vaccines have also been previously described [11], [12], [14]–[17]. The AMA1 protein used in this study was made using a modified procedure which will be reported elsewhere. In brief, the purification procedure as described in [15], was modified to increase the quality and scalability of the recombinant AMA-1 drug substances. The order and type of column chromatography procedures were revised to include capture by Ni-Sepharose-FF (GE Healthcare), purification using a Butyl-Sepharose 4 Fast Flow (GE Healthcare) hydrophobic interaction column and Q-Sepharose-HP (GE Healthcare) anion exchange column, followed by size exclusion chromatography (Superdex 75 (GE Healthcare)), sterile filtration using a 0.22 µm filter prior to bulk vialing and storage at <−70°C. All column chromatography procedures and solutions were in accordance with cGMP. Final GMP manufacture and fill of the vialed CPG 7909 and BSAM2/Alhydrogel® was conducted at the Biopharmaceutical Development Program, National Cancer Institute, Science Applications International Corporation, Frederick, Maryland.

A GLP toxicology study using a higher dose product (BSAM1/Alhydrogel® +/− CPG 7909, containing 160 µg MSP1_42_ and 80 µg AMA1 for a total of 240 µg antigenic proteins) was conducted in rabbits prior to study initiation (CRL Preclinical Services, Horsham, Pennsylvania), with no safety issues identified, and with induction of antibodies which inhibited growth in an in vitro parasite growth inhibition assay (GIA). BSAM2/Alhydrogel® and CPG 7909 were also separately evaluated in rabbit pyrogenicity tests using a cGLP compliant procedure, and none of the vaccine components were pyrogenic. Since the CPG 7909 used in the current study was manufactured in a new production facility (Avecia Biotechnology Inc., Milford Massachusetts), potency of the new CPG 7909 (without antigens) in comparison with the lot used in previous clinical trials was confirmed in a mouse study.

The components of the vaccine, BSAM-2/Alhydrogel® (low and high dose) and CPG 7909, were supplied separately in single-use vials to the study site pharmacist where a point of injection formulation was prepared. Vaccine components were stored at 2–8°C. Shortly before vaccination, 0.7 mL of BSAM-2/Alhydrogel® was withdrawn and added to a single dose vial containing 0.08 mL CPG 7909. The calculated total dose of antigen in the 0.55 mL injected volume was 39.5 µg (low dose) and 158.0 µg (high dose) of protein, shown as 40 µg or 160 µg for simplicity, with 564 µg of CPG 7909 administered with both the high and low doses. The stability of BSAM2/Alhydrogel® and CPG 7909 was assessed annually during the course of the study, and potency of BSAM2/Alhydrogel® in mice was assessed every six months. All components remained stable and potent during the course of the clinical trial.

Vaccines were given by intramuscular injection into the deltoid muscle in alternating arms when possible at study Days 0, 56, and 180. An independent Safety Monitoring Committee (SMC) reviewed adverse event data at scheduled safety reviews and as needed as the study progressed.

### Objectives

The primary objective of this study was to assess safety and reactogenicity of BSAM2/Alhydrogel®+CPG 7909 in malaria-naïve US adults. The secondary objective was to determine the antibody response of the combination vaccine to the AMA1 and MSP1_42_ proteins, as measured by antibody levels and parasite growth inhibition assay. Exploratory objectives included determination of the extent to which the antibody responses to the individual antigens (AMA1 and MSP1_42_) were correlated with each other.

### Outcomes

#### Safety

Volunteers were observed for 30 minutes after each vaccination to evaluate immediate adverse events and were given diary cards to record events occurring during the first week after each vaccination. The diary cards were used as a memory prompt and were reviewed with volunteers at follow up visits, when adverse events were recorded. Participants were seen at 3, 7, 14, and 28 days after each vaccination, and then approximately monthly for a total of 12 months (to study Day 360), with an additional follow up phone call scheduled at study Day 720. All adverse events were graded for severity and relationship to study product. Solicited injection site adverse events were pain, erythema, and induration. Solicited systemic adverse events were fever, headache, nausea, myalgia, arthralgia, and rash. Fever was graded as 1 = 100.4°F–101.5°F (38.0°C–38.6°C), 2 = 101.6°F–102.4°F (38.7°C–39.1°C), 3 = ≥102.5°F (≥39.2°C). Pain and other solicited adverse events were graded as follows: 0 = absent/none, 1 = easily tolerated, 2 = interferes with daily activity or treatment given, 3 = prevents daily activity. Unless otherwise specified, non-solicited adverse events were graded as 0 = none, 1 = no effect on activities of daily living and no treatment given, 2 = partial limitation in activities of daily living or treatment given, 3 = activities of daily living limited to <50% of baseline or medical evaluation required. Injection site erythema, swelling, and induration were graded based on the maximum diameter as follows: mild = >0 to 20 mm, moderate = 21–50 mm, and severe = >50 mm. Hematological (hemoglobin, white blood cell counts, and platelets) and biochemical (ALT and creatinine) laboratory parameters were measured at screening, on days of immunization, and 3 and 14 days after each vaccination; hematological parameters were also checked 7 days after each vaccination. Anti-dsDNA was checked as a marker for autoimmunity at screening, 14 days after the second and third vaccination, and at Day 360. Serious adverse events (SAEs) followed the FDA Code of Federal Regulation, and were defined as any adverse event that resulted in death; was life-threatening; required hospitalization; resulted in disability, incapacity, congenital anomaly, or birth defect; or any other event that required intervention to prevent such outcomes.

#### Immunogenicity

The standardized methodology for performing the enzyme-linked immunosorbent assay (ELISA) and the growth inhibition assay (GIA) have been described previously [11] [14] [18] [19]. For both AMA1 and MSP1_42_, FVO and 3D7 allele specific IgG antibody levels were assessed by ELISA at baseline for each vaccination, two weeks after each vaccination, and at Days 270 and 360. The minimum detection level of the assay was 33 ELISA units; for all antigens results less than these values were assigned a value of 17 ELISA units for statistical analysis. ELISA results were converted to ng/mL by multiplying the ELISA units by protein and parasite specific conversion factors (AMA1-FVO = 58.37, AMA1–3D7 = 47.28, MSP1-FVO = 21.64, MSP1–3D7 = 24.39). GIA was performed using purified IgG from serum collected two weeks post-second vaccination to assess biologic activity of the induced antibody against FVO and 3D7 parasites. In this assay, purified antibody was added to the parasite cultures at approximately the same concentration as present in the corresponding serum sample (10 mg/mL in GIA well).

### Sample Size

The study was powered to provide sufficient safety data before proceeding to a clinical trial in malaria-exposed adults. A sample size of 12 in each dose group gave a probability of 0.80 for detecting 1 or more adverse events that occurred with a frequency of 0.125 per participant. Fifteen volunteers per dose group were included in case of withdrawals or loss to follow-up.

### Group Assignment

Participants were sequentially enrolled in the two dose escalating groups. Within each group enrollment was staggered for safety purposes. Five volunteers were planned to be vaccinated at each dose level followed by vaccination of the remaining 10 volunteers in the group a minimum of 2 weeks later. Enrollment was further staggered after the occurrence of severe related adverse events, as described in the Results section.

### Blinding

This was an open label dose-escalating study, with both participants and those conducting clinical and laboratory assessments aware of the interventions received.

### Statistical Methods

The safety analysis was descriptive, with the frequency of local and systemic adverse events presented by dose group (40 µg low dose, and 160 µg high dose), and by vaccination (first, second, third). Data from all participants who received one or more vaccination are shown. For the immunological analysis only subjects who received all 3 vaccines and did not meet criteria for exclusion (i.e., received H1N1 vaccine close to the time of study vaccine administration) were used. Antibody results were analyzed as follows: concordance for allelic responses (FVO and 3D7) to the AMA1 and MSP1_42_ antigens was calculated using the random marginal agreement coefficient with the squared error loss function [20]. FVO and 3D7 responses at each time point were averaged, since responses for each allele were highly concordant, consistent with previous studies [12] [17]. Average FVO and 3D7 responses were plotted over time. Confidence intervals on geometric means used the t-distribution on log transformed responses. To test for a dose response (low versus high) a Wilcoxon Mann Whitney (WMW) test with Hodges-Lehmann confidence intervals was used to compare log transformed AMA1 and MSP1_42_ average antibody levels. To test for a further increase in antibody after third vaccination (Day 70 versus Day 194) a Wilcoxon signed rank test was used. GIA results were plotted against ELISA antibody levels in the GIA well for the homologous AMA1 and MSP1_42_ antigens. The correlation between antibody level and growth-inhibitory activity was tested by a Spearman rank test with confidence intervals calculated using Fisher's Z transformation on the correlation of the ranks. Calculations were done using R 2.14.2.

## Results

### Recruitment and Participant Flow

**Figure 1:**
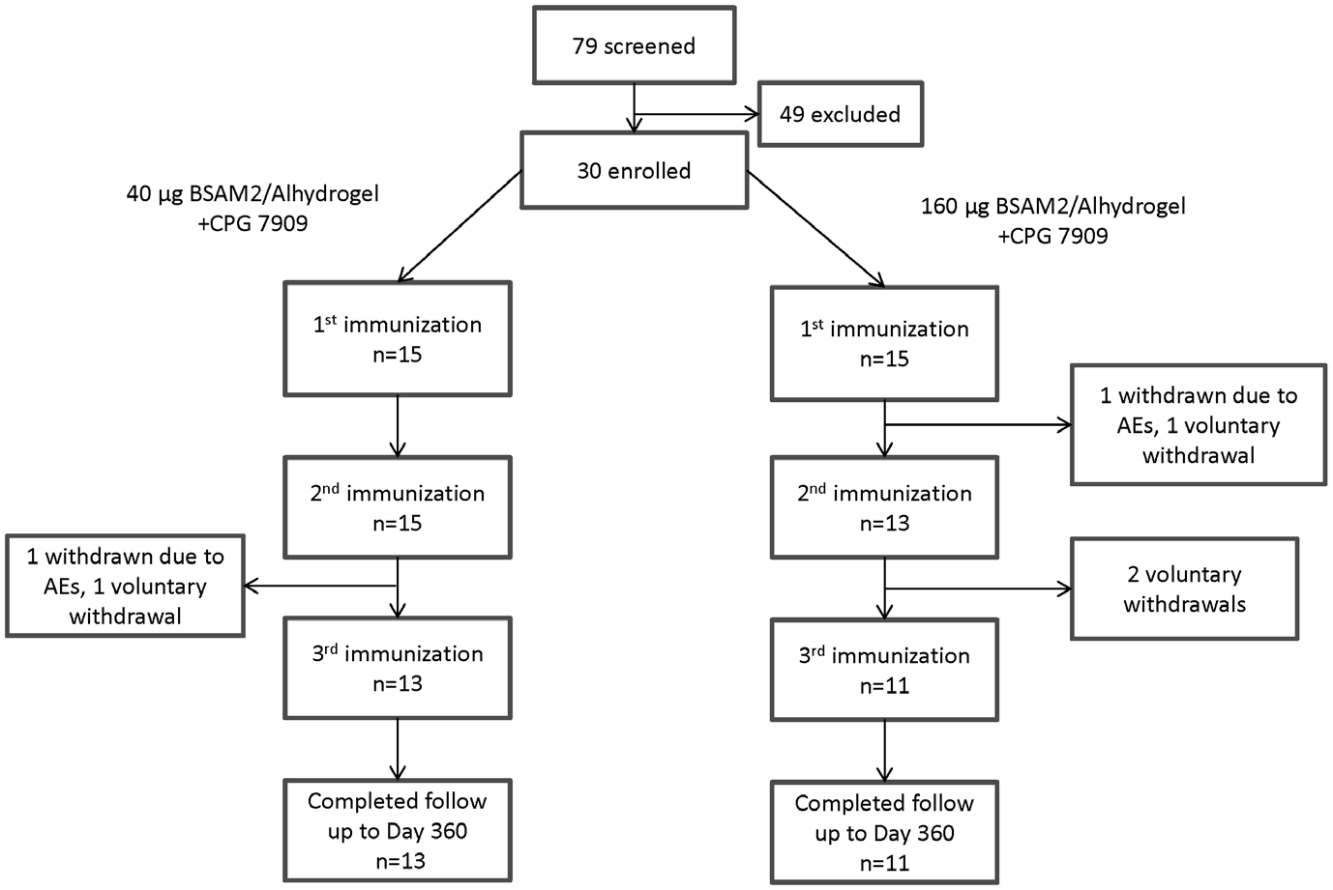
Participants flow sheet. Thirty (30) participants were enrolled, 24 participants completed vaccinations, and 24 completed follow-up to Day 360.

Using a general screening protocol, 79 volunteers were recruited from the Washington DC area. Fifty-six volunteers signed a study specific consent. Thirty-three of the seventy-nine did not meet eligibility criteria, 7 declined participation, and 9 others were eligible but were not enrolled. 30 healthy adults (13 female) were enrolled in the study. The mean age of participants was 33 years (range 18–50). Vaccinations began in June 2009 and were completed by March 2010. Participant flow through study Day 360 is shown in Figure 1. One participant in the low dose group was withdrawn after the second vaccination and one in the high dose group was withdrawn after first vaccination, both due to Grade 3 adverse events as described below. A second participant in the high dose group voluntarily withdrew after the second vaccination due to reported muscle soreness that was judged to be unrelated to the vaccine. These participants were taken off-treatment but remained in the study for safety follow-up. Three additional participants withdrew due to schedule conflicts, one after the first vaccination and the other two after the second vaccination. Twenty-six (13 in the low dose and 13 in the high dose groups) of 30 participants were contacted by telephone for additional safety follow up at approximately study Day 720.

### Numbers Analyzed

Safety data is presented for all participants who received at least one vaccination (n = 30). Immunogenicity results to Day 360 (last data point) were analyzed for 12 participants in the low dose group and 11 participants in the high dose group. Subjects who did not receive all three vaccines were excluded from the immunogenicity analyses, as well as one subject in the low dose group who received an H1N1 vaccine within a week of the third vaccination.

### Outcomes and Estimation

#### Safety

**Figure 2:**
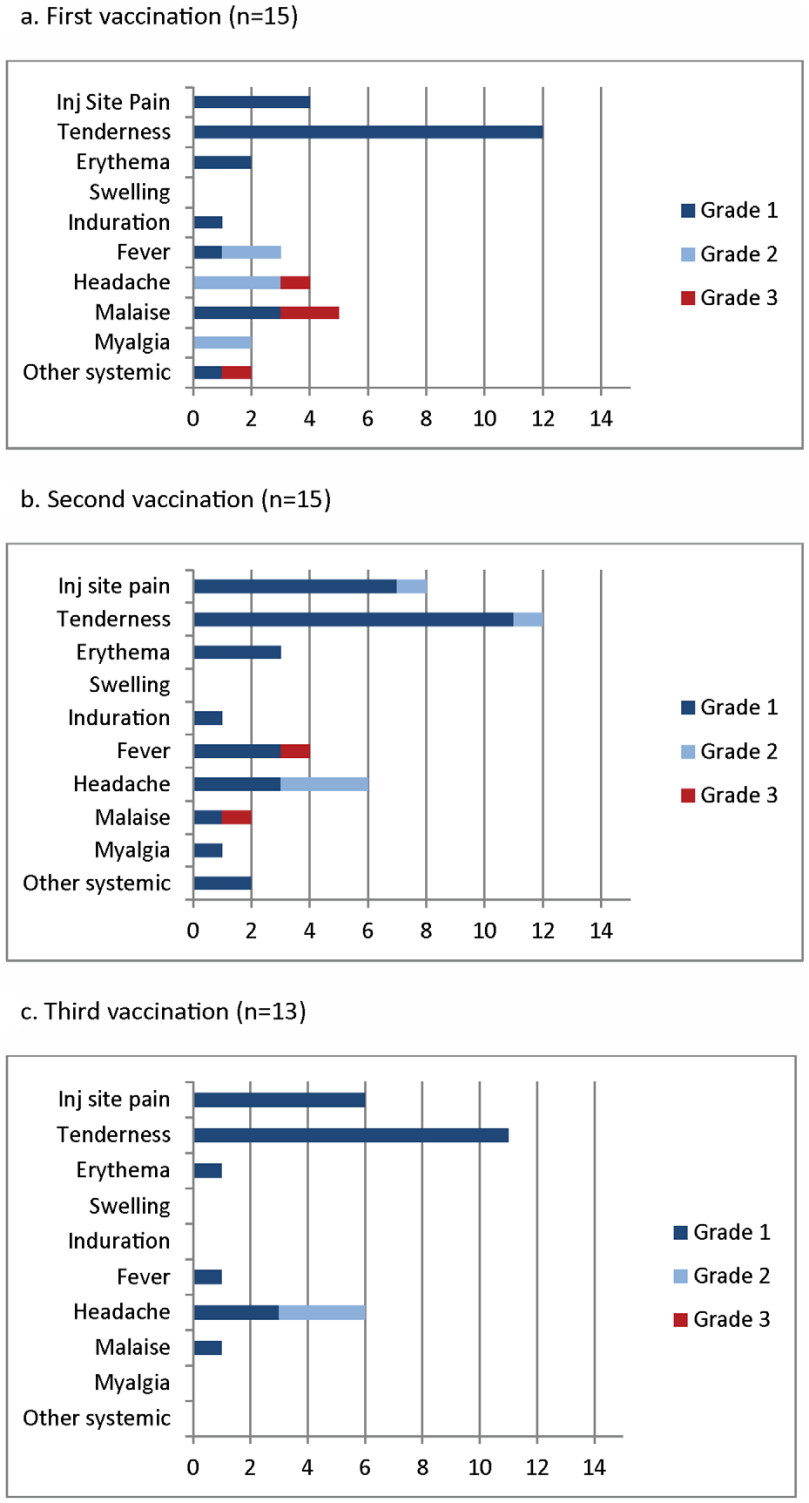
Local (injection site pain, tenderness, erythema, swelling, induration) and related systemic adverse events after first, second and third vaccinations in the low dose group. The x-axis shows the number of participants experiencing adverse events, with the highest severity event for each participant after each vaccination shown.

**Figure 3:**
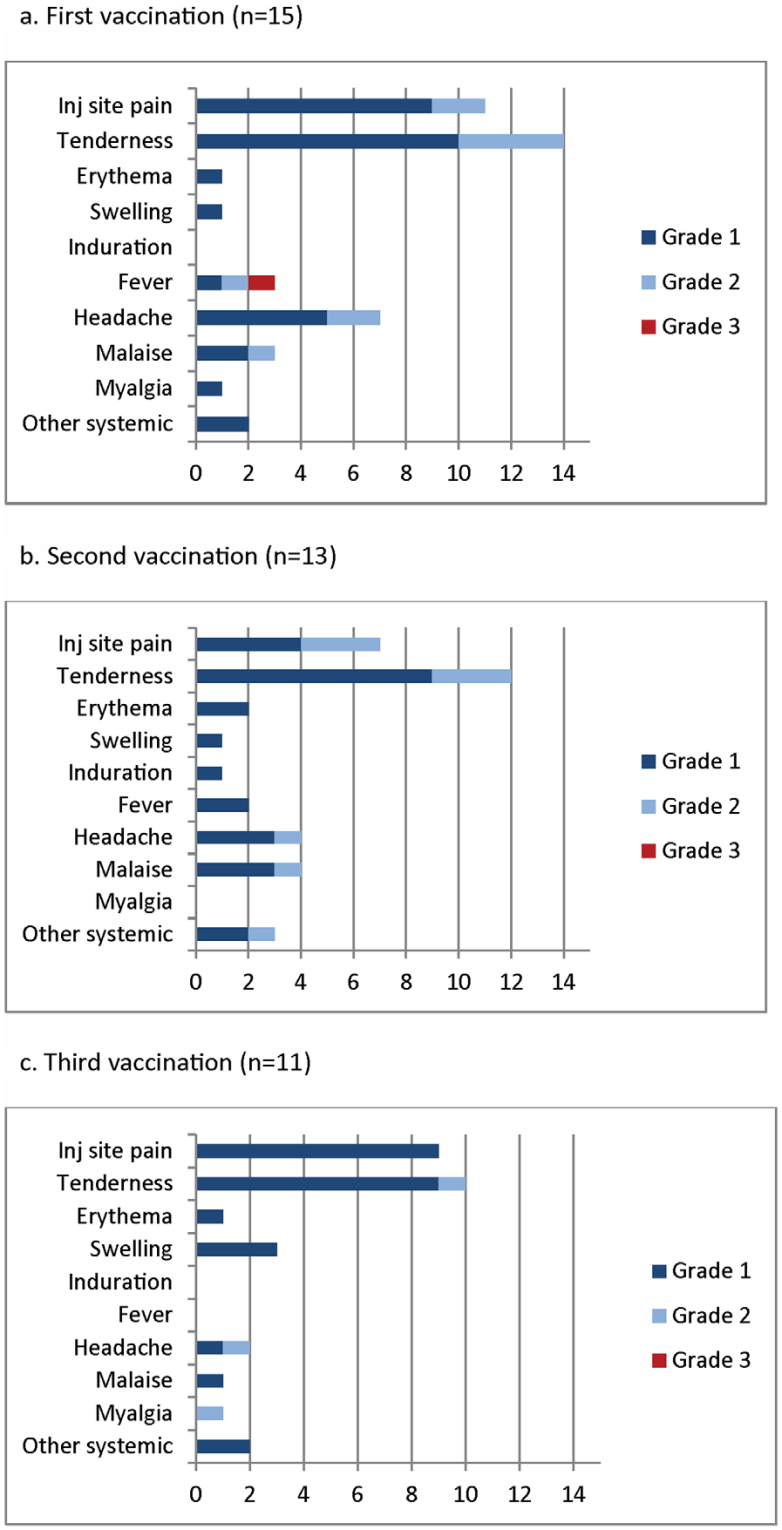
Local (injection site pain, tenderness, erythema, swelling, induration) and related systemic adverse events after first, second and third vaccinations in the high dose group. The x-axis shows the number of participants experiencing adverse events, with the highest severity event for each participant after each vaccination shown. Other systemic adverse events in both high and low dose groups were: diarrhea (1 severe, 2 mild), abdominal pain, nausea, and cough (all mild or moderate).

One serious adverse event unrelated to vaccination occurred during the study: a participant in the low dose group was electively hospitalized for surgery for cholelithiasis 2 months after his second vaccination. This participant had already been taken off treatment due to systemic adverse events following his first and second vaccinations (see below). No other serious adverse events occurred. One female in the high dose group became pregnant approximately three months after the third vaccination and elected to terminate the pregnancy; she continued follow up for safety and immunogenicity. Adverse events (AEs) related to vaccination for the low and high dose groups are shown in Figures 2 and 3. Most related AEs were mild or moderate, but three participants in the low dose group and one participant in the high dose group experienced severe systemic reactions consisting of fever, headache, malaise, and diarrhea. Two of these participants were withdrawn from treatment but remained in the study for safety follow-up: one participant in the low dose group who experienced moderate or severe fever, myalgia, and headache after first and second vaccinations; and one in the high dose group who experienced severe fever after first vaccination. Systemic adverse events had a flu-like pattern, including gastrointestinal symptoms in six participants and cough in one participant, but evaluation for an infectious etiology in a participant with a typical influenza syndrome was negative for influenza A and B (including H1N1, because enrollment and vaccination were concurrent with the 2009–2010 H1N1 influenza pandemic), parainfluenza 1, 2, and 3, respiratory syncytial virus, adenovirus, rhinovirus, and enterovirus; a second participant was negative for influenza A and B (including H1N1), Salmonella, Shigella, and Campylobacter. Both were in the low dose group. Flu-like symptoms typically had onset within 24 hours of vaccination and resolved within an additional 24 hours, and were relieved by over the counter (OTC) analgesics. After consultation with the Safety Monitoring Committee, participants were instructed to take OTC medications at the first sign of such symptoms, and no severe related events were seen subsequent to this change. One to two tablets of acetaminophen and/or ibuprofen and naproxen were taken on an as needed basis by thirteen participants who experienced either moderate to severe fever one to two days post vaccination. Of these thirteen participants, eight experienced repeated symptoms after more than one vaccination. Two of the four participants that experienced severe systemic reactions took only acetaminophen for their symptoms, while the remaining two participants took other non-steroidal anti-inflammatory products. No apparent difference in antibody response was noted between these four participants. Local adverse events were all mild or moderate, and were mostly pain and tenderness. Six participants (2 in the low dose group and 4 in the high dose group) had mild injection site swelling or induration. There was no apparent worsening of systemic or local reactogenicity after successive vaccinations or with increasing dose of vaccine, but this may have been masked by the pre-emptive use of analgesics with later vaccinations as the trial progressed.

Transient mild or moderate neutropenia/leukopenia is expected with the administration of CPG 7909 [21] and was observed in 9 participants in the low dose group and 7 in the high dose group. Transient thrombocytopenia is also expected and mild thrombocytopenia occurred in one participant in the low dose group. The only other laboratory adverse event considered related to vaccination was a mild transient elevation of creatinine in a low dose participant. CPG 7909 is a DNA analogue and anti-dsDNA is routinely monitored in clinical trials using CPG 7909. No elevations of anti-dsDNA occurred and there were no clinical events suggestive of autoimmune disease. No new onset of chronic or significant medical events was reported at the Day 720 follow up phone call.

#### Immunogenicity

**Figure 4:**
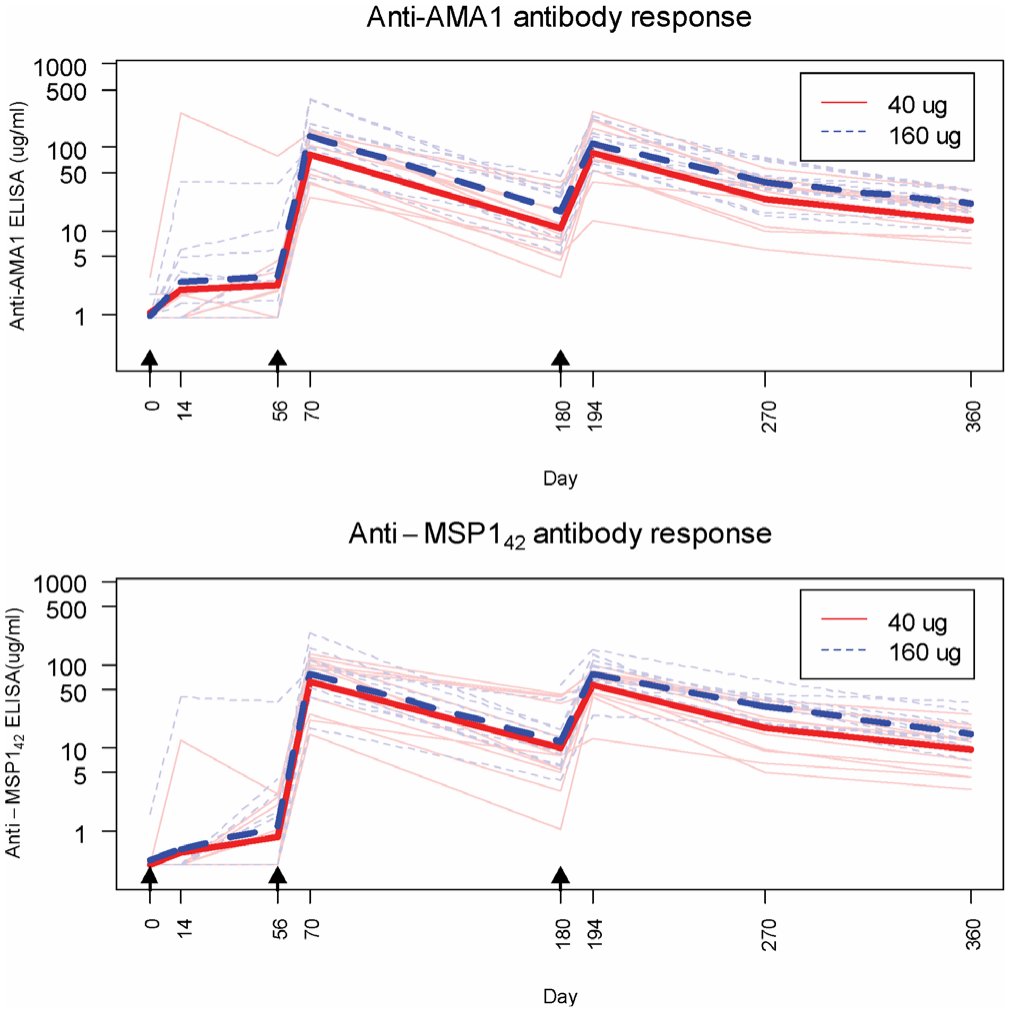
Antibody responses shown are the arithmetic mean of the FVO and 3D7 responses for each antigen for all volunteers who received all 3 vaccines and were not excluded per protocol (n = 23). Thicker lines show the geometric mean response; arrows indicate vaccinations. Mann-Whitney tests with Hodges-Lehmann confidence intervals were done to compare responses in low versus high dose groups and 2 weeks after 2^nd^ and 3^rd^ vaccinations (days 70 and 194); although the 160 µg group had slightly higher geometric means (AMA1 D70: Fold Change = 1.49 [95% CI 0.78, 2.92] p = 0.28; AMA1 D194: FC = 1.30 [0.68, 2.49] p = 0.61;MSP1 D70: FC = 1.18 [0.57,2.49] p = 0.70; MSP1 D 194: FC = 1.30 [0.85, 1.91]) p = 0.21; differences were not significant at the 0.05 level.

**Figure 5:**
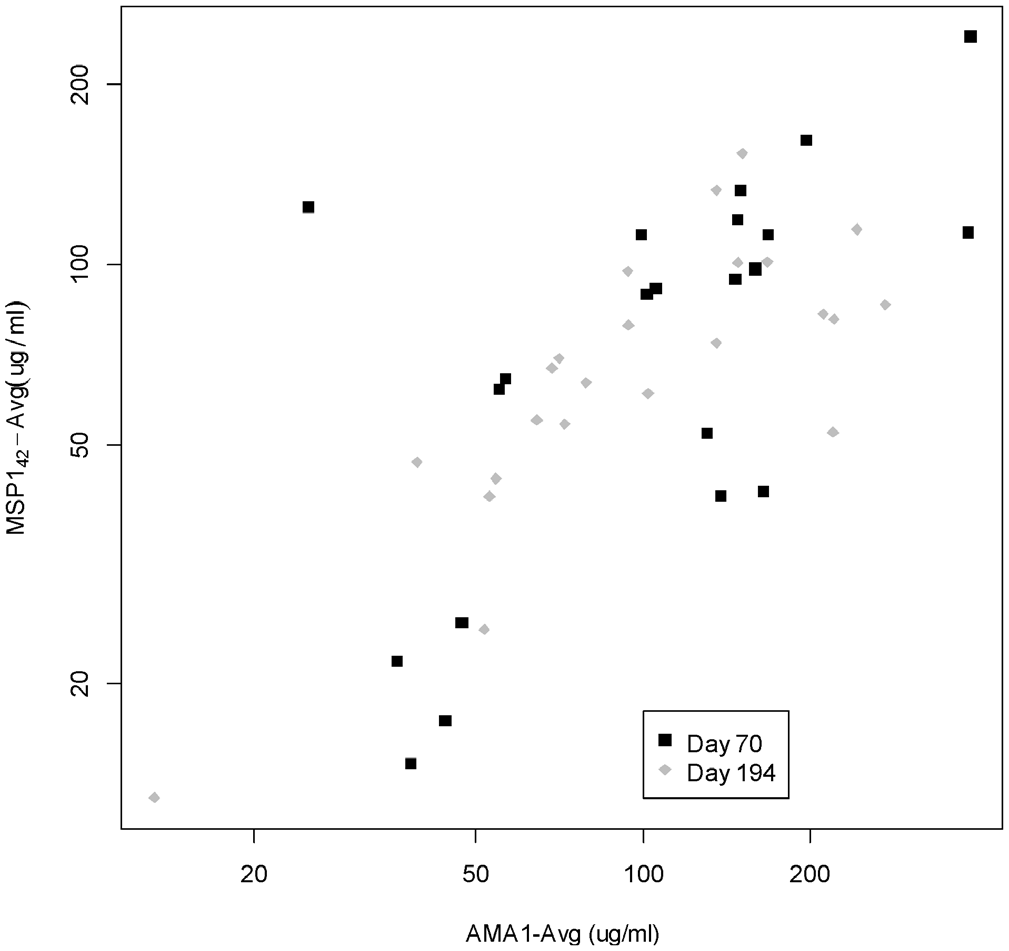
Correlation between average anti-AMA1 and -MSP1_42_ antibody responses at Day 70 and Day 194 (two weeks after second and third vaccinations). Day 70: Spearman correlation, r = 0.60 [95% CI 0.23, 0.82]; Day 194: Spearman's correlation, r = 0.75 [0.50,0.89].

Antibody responses after vaccination are shown in Figure 4. The concordance for the anti-AMA1 antibody responses by ELISA was 0.940 (95% CI 0.926, 0.951, p<0.0001) and for the anti-MSP1 antibody was 0.990 (95% CI 0.988, 0.993, p<0.0001). Both the high and low doses of vaccine were immunogenic, with responses seen after first vaccination that subsequently boosted after second vaccination. Day 70 geometric mean responses to AMA1were 84 µg/mL (95% CI: 53, 131) and 136 µg/mL (95% CI: 76, 244) for the low and high dose groups, respectively, and MSP1_42_ responses were 63 µg/mL (95% CI: 39, 103) and 78 µg/mL (95% CI: 42, 142) for the low and high dose groups, respectively. Day 194 geometric mean responses were 86 µg/ml (95% CI: 50, 148) and 113 µg/mL (95% CI: 80, 159) for low and high dose groups of AMA1 vaccines, respectively, and 57 µg/ml (95% CI: 40, 81) and 76 µg/mL (95% CI: 54, 108) for low and high dose groups of MSP1_42_ vaccines, respectively. There was no significant additional increase after the third vaccination, nor with the higher dose of vaccine (see Figure 4 legend). Anti-AMA1 and anti-MSP1_42_ antibody responses were similar overall and were correlated, as shown in Figure 5. Of the four participants who experienced severe systemic adverse events related to vaccination, one was a high responder and the others were low or mid-range responders.

**Figure 6:**
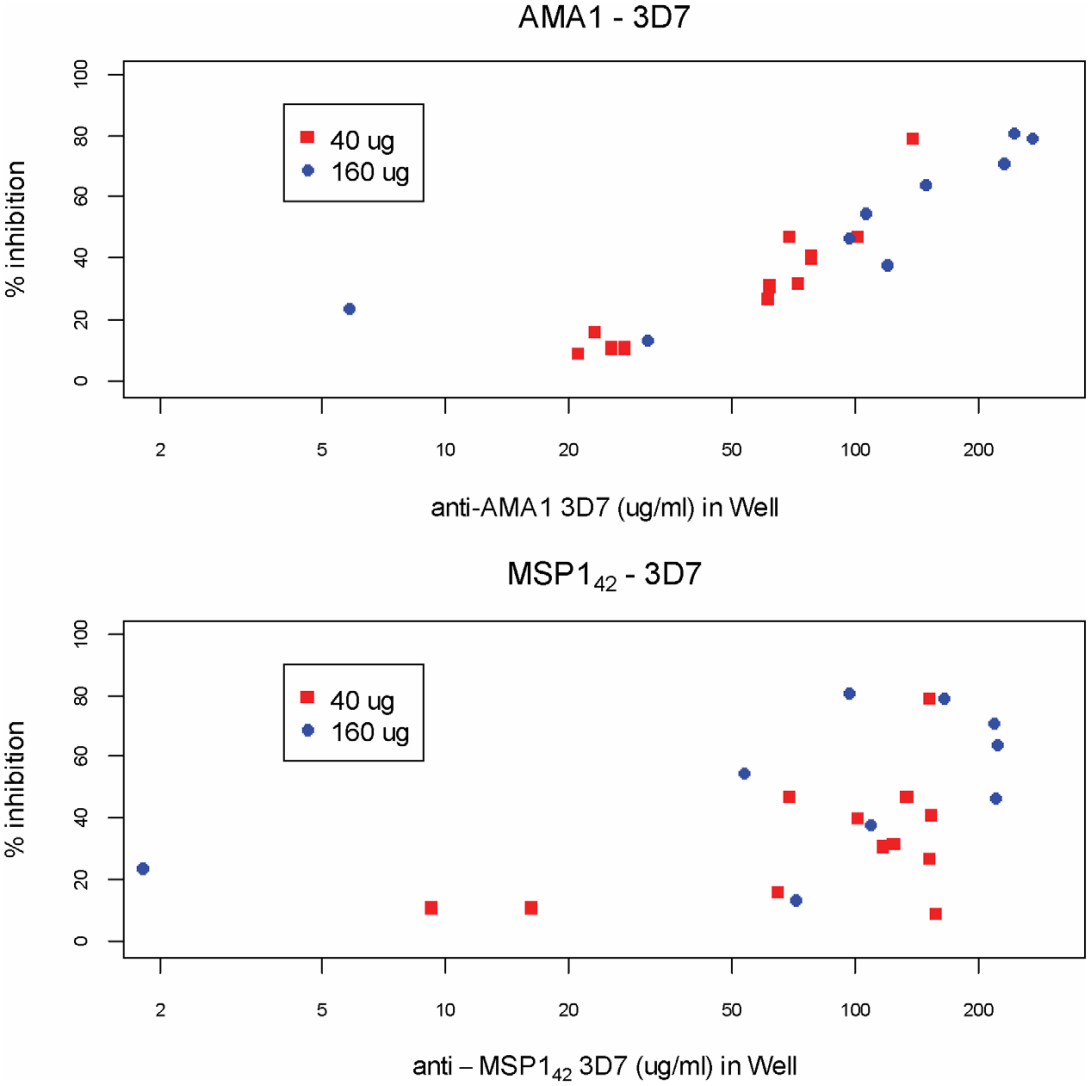
In vitro growth inhibition of homologous 3D7 parasites as a function of specific antibody concentration is shown. Purified IgG from Day 70 samples (two weeks after the second vaccination) were used at a concentration of 10 mg/mL.

Activity in the GIA of total purified IgG against homologous 3D7 parasites is shown in Figure 6. Maximum inhibition against 3D7 parasites was 81% with interquartile range (IQR) of 20–59% and median of 41%; maximum inhibition against FVO parasites was 55% with IQR of 4–15% and median of 7% (not shown). Growth inhibition against homologous parasites increased as a function of antibody against both AMA1 and MSP1_42_ antigens, but was more closely correlated with anti-AMA1 antibody (3D7: Spearman's correlation r = 0.92 [95% CI 0.81, 0.97]; FVO: Spearman's correlation r = 0.89 [95% CI 0.74,0.95]) than anti-MSP1_42_ antibody (3D7: Spearman's correlation r = 0.46 [95% CI 0.03, 0.74]; FVO: Spearman's correlation r = 0.62 [95% CI 0.25, 0.83]).

## Discussion

Immunity against blood stages of *P. falciparum* protects populations in endemic areas against clinical disease and is likely to be declining as exposure decreases in many populations. A blood stage vaccine, either alone or as a component of a multi-stage vaccine, is needed to protect against severe or epidemic malaria. BSAM2/Alhydrogel®+CPG 7909 was shown in this study to be moderately reactogenic, but induced high levels of antibodies against 2 allelic proteins each of AMA1 and MSP1_42_. The induced antibodies were active in the GIA.

### Interpretation

In our previous clinical trials AMA1-C1/Alhydrogel® with and without CPG 7909 and MSP1_42_-C1/Alhydrogel® with and without CPG 7909 were reasonably well tolerated, although systemic adverse events were more frequent and more severe when CPG 7909 was added to the Alhydrogel® formulations [11] [12] [17]. Preclinical pyrogenicity and toxicology studies on the vaccine and the components showed no safety concerns. Thus, the flu-like systemic events seen in this study were more severe than had been anticipated. These systemic events resulted in a modification of the protocol to allow for early administration of OTC analgesics. (Our standard practice has been to request that participants not take OTC medications after vaccination so as not to mask adverse events.) After this change was made, there were no severe related systemic adverse events and no participants were withdrawn from participation due to AEs. One clinical trial of AMA1-C1/Alhydrogel® with and without CPG 7909 has been conducted in malaria-exposed adults and showed the CPG 7909 formulation to be well tolerated, with minimal reactogenicity in this population [13]. Other studies have shown reduced reactogenicity to an adjuvanted vaccine in malaria-exposed versus malaria-naïve populations [22]–[] [24]. Thus the reactogenicity seen here, while unexpected, was considered to be manageable and to not preclude further evaluation of the vaccine in malaria-exposed adults.

Average antibody responses to AMA1 and MSP1_42_ were high and in the same range as those seen in previous trials of AMA1-C1 (at the same dose as in this study) and MSP1_42_-C1 vaccines (at a higher dose than in this study) with CPG 7909 [11] [12] [17]. Therefore, while immune competition was not directly assessed in this trial, there was no apparent reduction in immunogenicity due to combining AMA1 and MSP1_42_ antigens in one vaccine. On the other hand, the combination did not seem to broaden the immune coverage, as both the allelic responses and the average responses for the AMA1 and MSP1_42_ antigens were correlated. CPG 7909 has a dose-sparing effect [12], which is an important consideration for a vaccine intended for resource poor settings where cost of goods may limit distribution.

Biologic activity of the induced antibody was confirmed using purified total IgG (containing both anti-AMA1 and anti-MSP1_42_ antibody) in the GIA. Growth inhibitory activity was closely correlated with anti-AMA1 antibody levels. While in vitro growth inhibition has also been induced by vaccination with MSP1_42_/Alhydrogel®+CPG 7909, peak activity was much less (∼30%), compared to over 90% in some individuals vaccinated with AMA1-C1/Alhydrogel®+CPG 7909 [11] [12]. The amount of human IgG required to induce 50% inhibition of parasite growth in the GIA has been shown to be ∼6-fold higher for MSP1_42_ than for AMA1 [25]. Thus it appears likely that the growth inhibition seen here was predominantly due to responses to the AMA1 components of the vaccine. Growth inhibition with induced antibody has not been shown to be associated with protection against clinical malaria, although a significant inverse association with parasite multiplication rates in vaccinated participants was seen in a blood-stage challenge study of AMA1-C1/Alhydrogel®+CPG 7909 [26]. Evidence for an association between activity in the GIA and clinical malaria in field studies is conflicting [reviewed in 27]. The level of antibody needed for protection against blood stages of malaria is unknown, and may be quite high. Other mechanisms not measured by GIA may also contribute to protection [as reviewed in 9].

### Generalizability

This study in malaria-naïve adults has provided the initial safety and immunogenicity data needed prior to a study in malaria-exposed adults in Mali. Our previous experience with a multi-allelic AMA1 vaccine adjuvanted with Alhydrogel® and CPG 7909 showed the vaccine to be well tolerated and immunogenic in Malian adults [13], but no other studies of a CPG-adjuvanted vaccine have been conducted in malaria-exposed populations or in children and further trials are needed to confirm safety and immunogenicity in these populations.

AMA1 is highly diverse, with hundreds of haplotypes identified [28]. However, the inclusion of a limited number of alleles may provide protection against most field strains [29]. Allele-specific protection has been reported in a recent field trial of a highly immunogenic single-allelic (3D7) AMA1 vaccine, adjuvanted with AS02 [9]. MSP1_42_ is dimorphic in the MSP1_33_ region and largely conserved in the MSP1_19_ domain, and a bi-allelic vaccine should cover most strain diversity for this protein [16]. No protection was shown in a field trial of a MSP1_42_ vaccine which used the AS02 adjuvant, but allele-specific effects could not be ruled out [30]. No overall protection or allele-specific effects were shown in a field trial of AMA1/Alhydrogel®, but the formulation was not very immunogenic [31] [32]. Both AMA1 and MSP1_42_ have been shown to be protective in animal models [5] [6] [33] [34]. Thus a highly immunogenic, multi-allelic combination of AMA1 and MSP1_42_ warrants further evaluation in the field.

Matching of the allelic variants in a candidate vaccine with those present in field strains may be important in vaccine design, although limited availability of well-characterized antigens suitable for clinical development, together with limited knowledge of field diversity for most antigens, constrains this approach. Optimized formulation, including the use of potent adjuvants such as CPG 7909, also appears to be needed for the high antibody responses which are more likely to be protective [34]. While cellular responses were not analyzed in this study, careful formulation and use of adjuvants may also boost T cell responses, which were shown to be associated with protection in one model of blood stage immunity [35].

### Overall Evidence

More reactogenicity than expected was seen in this Phase 1 study of BSAM2/Alhydrogel®+CPG 7909 in malaria-naïve adults. However, the vaccine was well tolerated with more liberal use of OTC medications. Antibody responses to the AMA1 and MSP1_42_ antigens were high and functional activity of the induced antibody was demonstrated. A Phase 1 study in Malian adults is under way which will extend the safety profile of the vaccine and adjuvant, and also look for trends against clinical malaria endpoints.

## Supporting Information

Checklist S1 **CONSORT Checklist.**
(DOC)

Protocol S1 **Trial Protocol.**
(PDF)

## References

[1] WHO (2011) World Malaria Report 2011. http://www.who.int/malaria/world_malaria_report_2011/en/Ref Type: Internet Communication

[2] Murray CJL, Rosenfeld LC, Lim SS, Andrews KG, Foreman KJ, et al (2012) Global malaria mortality between 1980 and 2010: a systematic analysis. Lancet 379 (9814) 413–31. 22305225 10.1016/S0140-6736(12)60034-8

[3] Agnandji ST, Lell B, Soulanoudjingar SS, Fernandes JF, Abossolo BP, et al (2011) First results of phase 3 trial of RTS,S/AS01 malaria vaccine in African children. N Engl J Med 365 (20) 1863–75. 22007715 10.1056/NEJMoa1102287

[4] Cohen S, McGregor IA, Carrington (1961) Gamma-globulin and acquired immunity to human malaria. Nature 192: 733–7. 13880318 10.1038/192733a0

[5] Singh S, Miura K, Zhou H, Muratova O, Keegan B, et al (2006) Immunity to recombinant plasmodium falciparum merozoite surface protein 1 (MSP1): protection in Aotus nancymai monkeys strongly correlates with anti-MSP1 antibody titer and in vitro parasite-inhibitory activity. Infect Immun 74 (8) 4573–80. 16861644 10.1128/IAI.01679-05 PMC1539572

[6] Stowers AW, Kennedy MC, Keegan BP, Saul A, Long CA, et al (2002) Vaccination of monkeys with recombinant Plasmodium falciparum apical membrane antigen 1 confers protection against blood-stage malaria. Infect Immun 70 (12) 6961–7. 12438375 10.1128/IAI.70.12.6961-6967.2002 PMC133036

[7] Ellis RD, Sagara I, Doumbo O, Wu Y (2010) Blood stage vaccines for Plasmodium falciparum: Current status and the way forward. Human Vaccines 6 (8) 627–34. 20519960 10.4161/hv.6.8.11446 PMC3056062

[8] Goodman AL, Draper SJ (2010) Blood-stage malaria vaccines - recent progress and future challenges. Ann Trop Med Parasitol 104 (3) 189–211. 20507694 10.1179/136485910X12647085215534

[9] Thera MA, Doumbo OK, Coulibaly D, Laurens MB, Ouattara A, et al (2011) A field trial to assess a blood-stage malaria vaccine. N Engl J Med 365 (11) 1004–13. 21916638 10.1056/NEJMoa1008115 PMC3242358

[10] Narum DL, Ogun SA, Batchelor AH, Holder AA (2006) Passive immunization with a multicomponent vaccine against conserved domains of apical membrane antigen 1 and 235-kilodalton rhoptry proteins protects mice against Plasmodium yoelii blood-stage challenge infection. Infect Immun 74 (10) 5529–36. 16988228 10.1128/IAI.00573-06 PMC1594904

[11] Mullen GE, Ellis RD, Miura K, Malkin E, Nolan C, et al (2008) Phase 1 Trial of AMA1-C1/Alhydrogel plus CPG 7909: An Asexual Blood-Stage Vaccine for Plasmodium falciparum Malaria. PLoS One 3 (8) e2940. 18698359 10.1371/journal.pone.0002940 PMC2491586

[12] Ellis RD, Martin LB, Shaffer D, Long CA, Miura K, et al (2010) Phase 1 Trial of the Plasmodium falciparum blood stage vaccine MSP142-C1/Alhydrogel with and without CPG 7909 in Malaria Naïve Adults. PLoS One 5 (1) e8787. 20107498 10.1371/journal.pone.0008787 PMC2809736

[13] Sagara I, Ellis RD, Dicko A, Niambele MB, Kamate B, et al (2009) A randomized and controlled Phase 1 study of the safety and immunogenicity of the AMA1-C1/Alhydrogel+CPG 7909 vaccine for Plasmodium falciparum malaria in semi-immune Malian adults. Vaccine 27 (52) 7292–8. 19874925 10.1016/j.vaccine.2009.10.087 PMC2808270

[14] Malkin EM, Diemert DJ, McArthur JH, Perreault JR, Miles AP, et al (2005) Phase 1 clinical trial of apical membrane antigen 1: an asexual blood-stage vaccine for Plasmodium falciparum malaria. Infect Immun 73 (6) 3677–85. 15908397 10.1128/IAI.73.6.3677-3685.2005 PMC1111886

[15] Giersing B, Miura K, Shimp R, Wang J, Zhou H, et al (2005) Posttranslational Modification of Recombinant Plasmodium falciparum Apical Membrane Antigen 1: Impact on Functional Immune Responses to a Malaria Vaccine Candidate. Infect Immun 73 (7) 3963–70. 15972483 10.1128/IAI.73.7.3963-3970.2005 PMC1168543

[16] Malkin E, Long CA, Stowers AW, Zou L, Singh S, et al (2007) Phase 1 Study of Two Merozoite Surface Protein 1 (MSP1(42)) Vaccines for Plasmodium falciparum Malaria. PLoS Clin Trials 2 (4) e12. 17415408 10.1371/journal.pctr.0020012 PMC1847697

[17] Ellis RD, Mullen GE, Pierce M, Martin LB, Miura K, et al (2009) A Phase 1 study of the blood-stage malaria vaccine candidate AMA1-C1/Alhydrogel with CPG 7909, using two different formulations and dosing intervals. Vaccine 24;27 (31) 4104–9. 10.1016/j.vaccine.2009.04.077 PMC2722923 19410624

[18] Miura K, Orcutt AC, Muratova OV, Miller LH, Saul A, et al (2008) Development and characterization of a standardized ELISA including a reference serum on each plate to detect antibodies induced by experimental malaria vaccines. Vaccine 26 (2) 193–200. 18054414 10.1016/j.vaccine.2007.10.064 PMC2253722

[19] Miura K, Zhou H, Diouf A, Moretz SE, Fay MP, et al (2009) Anti-apical-membrane-antigen-1 antibody is more effective than anti-42-kilodalton-merozoite-surface-protein-1 antibody in inhibiting plasmodium falciparum growth, as determined by the in vitro growth inhibition assay. Clin Vaccine Immunol 16 (7) 963–8 Epub 2009 May 13. 19439523 10.1128/CVI.00042-09 PMC2708396

[20] Fay MP (2005) Random marginal agreement coefficients: rethinking the adjustment for chance when measuring agreement. Biostatistics 6: 171–80. 15618535 10.1093/biostatistics/kxh027

[21] Krieg AM, Efler SM, Witpoth M, Al Adhami MJ, Davis HL (2004) Induction of systemic Th1-like innate immunity in normal volunteers following subcutaneous but not intravenous admiration of CPG 7909, a synthetic B-class CpG oligodeoxynucleotide TLR9 agonist. J Immunotherapy 27 (6) 460–71. 10.1097/00002371-200411000-00006 15534490

[22] Saul A, Lawrence G, Smillie A, Rzepczyk CM, Reed C, et al (1999) Human phase I vaccine trials of 3 recombinant asexual stage malaria antigens with Montanide ISA720 adjuvant. Vaccine 17 (23–24) 3145–59. 10462251 10.1016/s0264-410x(99)00175-9

[23] Genton B, Al-Yaman F, Anders R, Saul A, Brown G, et al (2000) Safety and immunogenicity of a three-component blood-stage malaria vaccine in adults living in an endemic area of Papua New Guinea. Vaccine 18 (23) 2504–11. 10775784 10.1016/s0264-410x(00)00036-0

[24] Genton B, Al-Yaman F, Betuela I, Anders RF, Saul A, et al (2003) Safety and immunogenicity of a three-component blood-stage malaria vaccine (MSP1, MSP2, RESA) against Plasmodium falciparum in Papua New Guinean children. Vaccine 22 (1) 30–41. 14604568 10.1016/s0264-410x(03)00536-x

[25] Miura K, Zhou H, Diouf A, Moretz SE, Fay MP, et al (2009) Anti-apical-membrane-antigen-1 antibody is more effective than anti-42-kilodalton-merozoite-surface-protein-1 antibody in inhibiting plasmodium falciparum growth, as determined by the in vitro growth inhibition assay. Clin Vaccine Immunol 16 (7) 963–8. 19439523 10.1128/CVI.00042-09 PMC2708396

[26] Duncan CJ, Sheehy SH, Ewer KJ, Douglas AD, Collins KA, et al (2011) Impact on malaria parasite multiplication rates in infected volunteers of the protein-in-adjuvant vaccine AMA1-C1/Alhydrogel+CPG 7909. PLoS One 6 (7) e22271. 21799809 10.1371/journal.pone.0022271 PMC3142129

[27] Duncan CJA, Hill AVS, Ellis RD (2012) Can growth inhibition assays (GIA) predict blood-stage malaria vaccine efficacy? Human Vaccines and Immunotherapeutics 8 (6) Epub ahead of print. 10.4161/hv.19712 PMC3495712 22508415

[28] Takala SL, Coulibaly D, Thera MA, Batchelor AH, Cummings MP, et al (2009) Extreme polymorphism in a vaccine antigen and risk of clinical malaria: implications for vaccine development. Sci Transl Med 1 (2) 2ra5. 10.1126/scitranslmed.3000257 PMC2822345 20165550

[29] Duan J, Mu J, Thera MA, Joy D, Kosakovsky Pond SL, et al (2008) Population structure of the genes encoding the polymorphic Plasmodium falciparum apical membrane antigen 1: implications for vaccine design. Proc Natl Acad Sci U S A 105 (22) 7857–62. 18515425 10.1073/pnas.0802328105 PMC2409418

[30] Ogutu BR, Apollo OJ, McKinney D, Okoth W, Siangla J, et al (2009) Blood stage malaria vaccine eliciting high antigen-specific antibody concentrations confers no protection to young children in Western Kenya. PLoS One 4 (3) e4708. 19262754 10.1371/journal.pone.0004708 PMC2650803

[31] Sagara I, Dicko A, Ellis RD, Fay MP, Diawara SI, et al (2009) A randomized controlled phase 2 trial of the blood stage AMA1-C1/Alhydrogel malaria vaccine in children in Mali. Vaccine 27 (23) 3090–8. 19428923 10.1016/j.vaccine.2009.03.014 PMC2713037

[32] Ouattara A, Mu J, Takala-Harrison S, Saye R, Sagara I, et al (2010) Lack of allele-specific efficacy of a bivalent AMA1 malaria vaccine. Malar J 9: 175. 20565971 10.1186/1475-2875-9-175 PMC2908102

[33] Lyon JA, Angov E, Fay MP, Sullivan JS, Girourd AS, et al (2008) Protection induced by Plasmodium falciparum MSP1(42) is strain-specific, antigen and adjuvant dependent, and correlates with antibody responses. PLoS One 3 (7) e2830. 18665258 10.1371/journal.pone.0002830 PMC2474699

[34] Dutta S, Sullivan JS, Grady KK, Haynes JD, Komisar J, et al (2009) High antibody titer against apical membrane antigen-1 is required to protect against malaria in the Aotus model. PLoS One 4 (12) e8138. 19997632 10.1371/journal.pone.0008138 PMC2780715

[35] Pombo DJ, Lawrence G, Hirunpetcharat C, Rzepczyk C, Bryden M, et al (2002) Immunity to malaria after administration of ultra-low doses of red cells infected with Plasmodium falciparum. Lancet 360 (9333) 610–7. 12241933 10.1016/S0140-6736(02)09784-2

