# Supplementary material for: Phase 1 Study in Malaria Naïve Adults of BSAM2/Alhydrogel®+CPG 7909, a Blood Stage Vaccine against P. falciparum Malaria
Source: PLoS One. 2012 Oct 4;7(10):e46094. doi: 10.1371/journal.pone.0046094 (PMC3464250; doi:10.1371/journal.pone.0046094)
Supplement: Protocol S1 — Trial Protocol. (PDF) [file pone.0046094.s002.pdf]

Phase 1 Study of the Safety and Immunogenicity of BSAM-2/Alhydrogel®+CPG 7909, an Asexual Blood Stage Vaccine for *Plasmodium falciparum* Malaria in Adults in the US and in Bancoumana, Mali

NIAID Protocol Number: 09-I-N134
Project Assurance: FWA #00005897
Multi-institution: Yes

Western IRB Protocol Number: 20090887
Project Assurance: FWA #00000287

FMPOS Protocol Number: 09-55
Project Assurance: FWA #00001769

Sponsored by:
National Institute of Allergy and Infectious Diseases (NIAID)
Laboratory of Malaria Immunology and Vaccinology (LMIV)
National Institutes of Health

Pharmaceutical Support Provided by:
Coley Pharmaceutical Group, a Pfizer Company
340 Terry Fox Drive, Suite 200
Ottawa ON Canada K2K3A2

IND Sponsor:
Regulatory Compliance and Human Subjects Protection Branch (RCHSPB)
National Institutes of Allergy and Infectious Diseases (NIAID)
IND: BB-IND #14022

Principal Investigators:
Anna Durbin, MD, Center for Immunization Research (CIR)
Issaka Sagara, MD, MSPH University of Bamako
Ruth D. Ellis, MD, MPH, NIAID

Medical Monitor (Mali):
Mamadou Dembélé, MD
Service de Medicine Interne
Centre Hospitalo-Universitaire du Point G
50 02/ +223 2022 5003 Cel: + 223 7604 93 87
Email: hassiramadydembele@yahoo.fr

Version 7.0: 18 November 2011

CONFIDENTIAL

Team Roster

Center for Immunization Research Principal Investigator:

Anna Durbin, MD
Center for Immunization Research (CIR)
Johns Hopkins School of Public Health
624 N. Broadway
Baltimore, MD 21205
410-614-4736

CIR Co-Investigators:

Noreen Hynes, MD
Donna Shaffer, MS, RNCS, FNP
Beulah Sabundayo, RPh, MPH
Center for Immunization Research

University of Bamako Principal Investigator:

Issaka Sagara, MD, MSPH
Malaria Research and Training Center (MRTC)
Department of the Epidemiology of Parasitic Diseases (DEAP)
Faculty of Medicine, Pharmacy, and Dentistry (FMPOS), University of Bamako
BP 1805, Point G
Bamako, Mali
isagara@icermali.org
+223-2022-8109

University of Bamako Co-Investigators:

Alassane Dicko, MD, MS
Amagana Dolo, PharmD, PhD
Mahamadoun Hamady Assadou, MD
Mamady Kone, MD
Ousmane Guindo, MD
Mahamadou S. Sissoko, MD, MSPH
Merepen dite Agnes Guindo, PharmD
Renion Saye, PharmD
Boubacar Traoré, PharmD, PhD
Didier Doumtabe, PharmD
Safiatou Niare Doumbo, MD
Malaria Research and Training Center
MRTC/DEAP/FMPOS
Universite de Bamako
B.P. 1805

NIAID Principal Investigator/Coordinating Investigator:
Ruth D. Ellis, MD, MPH
LMIV/NIAID/NIH
5640 Fishers Lane, Twinbrook I
Rockville, Maryland 20852
ellisru@niaid.nih.gov
fax: 301-435-1962
tel: 301-435-3064

NIAID Co Investigator: Yimin Wu, PhD
LMIV/NIAID/NIH

Senior Investigators: Ogobara Doumbo, MD, PhD
Dapa Aly Diallo, MD, PhD
MRTC, University of Bamako

Patrick Duffy, MD
LMIV/NIAID/NIH

Biostatistician: Michael P. Fay, PhD
NIAID/NIH
6700 A Rockledge Drive
Room 5133
Bethesda, Maryland 20892
(301) 451-5124

Participating Sites

**Clinical Trial Sites**	Center for Immunization Research<br>Johns Hopkins Bloomberg School of Public Health<br>2112 F Street, NW, Suite 305<br>Washington DC 20037<br>(202) 223-6564
	Bancoumana Malaria Vaccine Center<br>MRTC/DEAP/FMPOS<br>Bancoumana, Mali
**Immunology Laboratories**	LMIV/NIAID/NIH<br>5640 Fishers Lane, Twinbrook I<br>Rockville, Maryland 20852<br>(301) 435-3405
	Laboratory of Immunogenetics<br>NIAID/NIH<br>2441 Parklawn Drive, Twinbrook II<br>Rockville, Maryland 20852
	Laboratory of Malaria and Vector Research (LMVR)<br>NIAID/NIH<br>12735 Twinbrook Parkway<br>Twinbrook III<br>Bethesda, MD 20892-8132
	MRTC/DEAP Immunology Laboratory<br>FMPOS, University of Bamako<br>BP1805, Point G<br>Bamako, Mali
**Clinical Laboratories**	Quest Diagnostics<br>1901 Sulphur Springs Road<br>Baltimore, Maryland 21227<br>(800) 368-2576
	MRTC/DEAP Clinical Laboratory<br>FMPOS, University of Bamako<br>BP1805, Point G<br>Bamako, Mali

TABLE OF CONTENTS

Page

LIST OF ABBREVIATIONS 9

PRÉCIS 11

1. INTRODUCTION AND RATIONALE 13

1.1 Malaria Epidemiology and Rationale for Vaccine 13
1.2 AMA1 Protein 13
1.3 $MSP1_{42}$ Protein 14
1.4 Combination Vaccine 14
1.5 Description of Study Agent 15
1.5.1 AMA1-C1 Drug Substance 15
1.5.2 $MSP1_{42}$-C1 Drug Substance 15
1.5.3 BSAM-2 15
1.5.4 Alhydrogel® 16
1.5.5 BSAM-2/Alhydrogel® 16
1.5.6 CPG 7909 16
1.6 Previous Human Experience 16
1.6.1 AMA1-C1/Alhydrogel® 16
1.6.2 AMA1-C1/Alhydrogel®+CPG 7909 17
1.6.3 AMA1-C1/Alhydrogel®+CPG 7909 (phosphate and saline) 18
1.6.4 $MSP1_{42}$/Alhydrogel® 18
1.6.5 $MSP1_{42}$-C1/Alhydrogel®+ CPG 7909 19
1.6.6 Clinical Experience with BSAM-2/Alhydrogel+CPG 7909 to date 19
1.6.7 Previous Clinical Experience with CPG 7909 (VaxImmune®) 19
1.6.8 Clinical Development Plan 20

2. STUDY OBJECTIVES 20

2.1 Primary Objective 20
2.2 Secondary Objectives 20
2.3 Exploratory Objectives 21

3. STUDY DESIGN 21

3.1 Overall Design 21
3.2 Study Endpoints 22
3.2.1 Primary Endpoints 22
3.2.2 Secondary Endpoints 23
3.2.3 Exploratory Endpoints 23
3.3 Sample Size and Estimated Duration of Study 23

4. STUDY POPULATION 23

4.1 Description of Population and Site 23
4.1.1 US 23
4.1.2 Mali 23
4.2 Recruitment: US 23
4.3 Recruitment: Mali 24

4.3.1 Community Consent ... *24*
4.3.2 Individual Recruitment and Informed Consent ... *24*
4.4 Inclusion Criteria ... 25
4.5 Exclusion Criteria (US) ... 25
4.6 Exclusion Criteria (Mali) ... 26
4.7 Rationale for Different Inclusion/Exclusion Criteria in US and Mali Sites ... 28

5. STUDY AGENTS ... 28

5.1 Vaccine ... 28
5.2 Euvax B/Hepatitis B Vaccine ... 28
5.3 Vaccine Administration ... 29
5.4 Vaccine Storage ... 29
5.5 Vaccine Accountability ... 29
5.5.1 Disposition ... *29*
5.5.2 Documentation ... *29*

6. STUDY PROCEDURES ... 29

6.1 Screening ... 29
6.2 Assignment to Groups ... 30
6.3 Blinding and Randomization (Mali) ... 30
6.4 Immunization Procedure ... 31
6.5 Clinical Monitoring and Evaluation ... 31
6.6 Symptom Memory Enhancement Tool (US Only) ... 38
6.7 Photographs of Rash or Injection Site Reactions ... 38
6.8 Contraindications to Vaccination ... 38
6.9 Indications for Deferral of Vaccination ... 38
6.10 Subject Withdrawal Criteria ... 39
6.11 Replacement of Subjects ... 39
6.12 Treatments that Could Potentially Interfere with Vaccine-induced Immunity ... 39
6.13 Clinical Laboratory Testing ... 40
6.14 Immunologic Laboratory Testing ... 40
6.14.1 Antibody Assay (ELISA) ... *40*
6.14.2 Growth Inhibition Assay (GIA) ... *41*
6.14.3 B-Cell Assays ... *41*
6.14.4 T-Cell Assays ... *41*
6.14.5 Filter paper ... *41*

7. USE, STORAGE, AND TRACKING OF SPECIMENS AND DATA ... 42

8. RETENTION OF SPECIMENS FOR FUTURE USE ... 42

9. SAFETY AND ADVERSE EVENT REPORTING ... 43

9.1 Adverse Events ... 43
9.2 Serious Adverse Events ... 43
9.3 Assessment of Adverse Events ... 44
9.3.1 Identification of Adverse Events ... *44*
9.3.2 Determination of Severity ... *45*
9.3.3 Association with Receipt of the Study Vaccine ... *45*

9.4 Serious Adverse Event Reporting 46
9.4.1 Reporting to IND Sponsor 46
9.4.2 Reporting to Coley Pharmaceutical Group, a Pfizer Company ***Error! Bookmark not defined.***
9.4.3 Reporting to the IRBs .... ***Error! Bookmark not defined.***
9.4.4 Reporting of Pregnancy .... ***Error! Bookmark not defined.***
9.5 Halting Rules 49

10. CLINICAL MONITORING 49

10.1 Site Monitoring Plan 49
10.2 Safety Monitoring Plan 50
10.2.1 Safety Monitoring Committee (SMC) 50
10.2.2 Medical Monitor 50

11. STATISTICAL CONSIDERATIONS 51

11.1 Description of Statistical Methods 51
11.1.1 Primary Objective 51
11.1.2 Secondary Objective 51
11.1.3 Exploratory Objectives 52
11.2 Sample Size and Power Calculations 52

12. HUMAN SUBJECT PROTECTIONS AND ETHICAL OBLIGATIONS ... 52

12.1 Institutional Review Board 52
12.2 Informed Consent Process 52
12.3 Justification for Exclusion of Children 53
12.4 Participant Confidentiality 53
12.5 Risks 53
12.5.1 Venipuncture 53
12.5.2 Immunization 54
12.6 Benefits 55
12.7 Compensation 55
12.7.1 US 55
12.7.2 Mali 56

13. DATA HANDLING AND RECORD KEEPING 56

13.1 Source Documentation 56
13.2 Retention of Study Records 57
13.3 Protocol Revisions 57
13.4 Publication Policy 57

14. REFERENCES 58

APPENDIX A: MALARIA COMPREHENSION EXAM 62

APPENDIX B: SCHEDULE OF PROCEDURES/EVALUATIONS 66

APPENDIX C: TOXICITY TABLE 68

APPENDIX D: TOXICITY TABLE FOR GRADING LABORATORY ADVERSE EVENTS 69

APPENDIX E: TABLE FOR GRADING ADVERSE EVENTS 70

LIST OF ABBREVIATIONS

ADR	adverse drug reaction
AE	adverse event/adverse experience
ALT	alanine transaminase
AMA1	Apical Membrane Antigen 1
anti-dsDNA	anti-double stranded DNA
AVA	anthrax vaccine absorbed
CBC	complete blood count
CFR	Code of Federal Regulations
CIB	Clinical Investigator’s Brochure
CIOMS	Council for International Organizations of Medical Sciences
CIR	Center for Immunization Research (Johns Hopkins Univ.)
CLIA	Clinical Laboratory Improvement Amendment of 1988
COI	Conflict of Interest
CONSORT	Consolidated Standards of Reporting Trials
CRADA	Cooperative Research and Development Agreement
CRF	case report form
CRO	Contract Research Organization
CRIMSON	Clinical Research Information Management System of the NIAID
DCR	Division of Clinical Research
DHHS	Department of Health and Human Services
DSMB	Data and Safety Monitoring Board
DSMC	Data and Safety Monitoring Committee
ELISA	enzyme-linked immunosorbent assay
ELISPOT	enzyme-linked immunosorbent spot assay
FDA	Food and Drug Administration
FWA	Federal Wide Assurance
GCP	Good Clinical Practice
GIA	(parasite) growth inhibition assay
HBsAg	hepatitis B surface antigen
HCV	hepatitis C virus
HIPAA	Health Insurance Portability and Accountability Act
HIV	human immunodeficiency virus
IB	Investigator’s Brochure
ICF	Informed Consent Form
ICH	International Conference on Harmonization
IDE	Investigational Device Exemption
IEC	Independent or Institutional Ethics Committee
IM	intramuscular
IND	Investigational New Drug
IRB	Institutional Review Board
ISM	Independent Safety Monitor
MedDRA©	Medical Dictionary for Regulatory Activities
MOP	Manual of Procedures/Manual of Operations

LIST OF ABBREVIATIONS

MRTC	Malaria Research and Training Center
MSP1	Merozoite Surface Protein 1
LMIV	Laboratory of Malaria Immunology and Vaccinology (of NIAID)
N	number (typically refers to subjects or participants)
NCI	National Cancer Institute, NIH
NDA	New Drug Application
NIAID	National Institute of Allergy and Infectious Diseases, NIH
NIH	National Institutes of Health
OHRP	Office for Human Research Protections
OHSR	Office of Human Subjects Research
PHI	Protected Health Information
PI	Principal Investigator
PK	pharmacokinetics
QA	quality assurance
QC	quality control
RCHSPB	Regulatory Compliance and Human Subjects Protection Branch
RCHSPP	Regulatory Compliance and Human Subjects Protection Program
SAE	serious adverse event/serious adverse experience
SMC	Safety Monitoring Committee
SOP	Standard Operating Procedure
WBC	white blood cell
WHO	World Health Organization
β-hCG	human choriogonadotropin

PRÉCIS

Full Title	Phase 1 Study of the Safety and Immunogenicity of BSAM-2/Alhydrogel®+CPG 7909, an Asexual Blood Stage Vaccine for *Plasmodium falciparum* Malaria in Adults in the US and in Bancoumana, Mali
Short Title	Phase 1 Study of BSAM-2 + CPG 7909
Clinical Phase	1
IND Sponsor	RCHSPB
Conducted By	Center for Immunization Research (US), University of Bamako (Mali)
Principal Investigators	Anna Durbin, MD (US), Issaka Sagara, MD, MSPH (Mali), Ruth D. Ellis, MD, MSPH (NIAID, Coordinating Investigator)
Sample Size (number vaccinated)	N = 30 (US)<br>N = 30 (Mali)
Accrual Ceiling	200 (US)<br>200 (Mali)
Study Population	Malaria-naïve US adults, semi-immune Malian adults
Accrual Period	May 2009 (US), October 2009 (Mali) (approximate)
Study Design	Randomized, open label (US)/single blinded (Mali), dose escalating Phase 1 clinical trial. The highest safe dose will be administrated in Mali after safety data in US adults have been reviewed by the SMC.
Study Duration	Start Date (approximate): May, 2009<br>End Date (approximate): December, 2011<br>Study participants will be enrolled for a total of 52 weeks, with additional passive follow up to 104 weeks in Mali, and an optional follow up visit at 104 weeks in the US.
Study Agent/Intervention Description	BSAM-2: A mixture of AMA1 and $MSP1_{42}$ proteins representing 2 different allelic variants. Equal mass proportions of 2 allelic forms of either AMA1 or $MSP1_{42}$ proteins are present within the vaccine. BSAM-2 is a tetravalent vaccine containing $MSP1_{42}$ and AMA1 in a 1:1:1:1 ratio. The BSAM-2 mixture was adsorbed onto aluminum hydroxide gel (Alhydrogel®) and will be mixed with CPG 7909 in a point-of-injection formulation.<br>In Mali, a comparator vaccine (Euvax B/Hepatitis B) will be given.
Primary Objective	To assess safety and reactogenicity of BSAM-2/Alhydrogel®+CPG 7909 in malaria-naïve US adults and semi-immune Malian adults.
Secondary Objectives	To determine the antibody response of the combination vaccine to the AMA1 and $MSP1_{42}$ proteins, as measured by antibody levels and parasite growth inhibition.
Exploratory Objectives	1. To determine the extent to which the antibody response to the individual antigens (AMA1 and $MSP1_{42}$) is correlated

	when the combination vaccine (BSAM-2/Alhydrogel®+CPG 7909) is given.<br>2. To determine T and B cell responses to vaccination.
Endpoints	1. Incidence of local and systemic adverse events.<br>2. Antibody levels elicited by BSAM-2/Alhydrogel®+CPG 7909 to AMA1 and $MSP1_{42}$ proteins, analogous to the FVO and 3D7 *P. falciparum* proteins, as judged by ELISA.<br>3. Inhibition of *P. falciparum* parasite growth in vitro at selected time points and the extent to which anti-AMA1 or anti-$MSP1_{42}$ antibody levels correlate with growth inhibition.<br>4. T cell responses to AMA1 and $MSP1_{42}$ as measured by cytokine levels and ELISPOT assays<br>5. The number and proportion of AMA1 and $MSP1_{42}$ specific memory B cells as measured by flow cytometry and ELISPOT assays.

1. INTRODUCTION AND RATIONALE

MALARIA EPIDEMIOLOGY AND RATIONALE FOR VACCINE

Globally, the *Plasmodium falciparum* parasite is responsible for at least 247 million acute cases of malaria each year, with about 1 million deaths [1]. Approximately 90% of these deaths, the majority in children under 5 years of age, occur in Africa due to infection with *P. falciparum*. Morbidity and mortality caused by malaria also has significant direct and indirect costs on the economic development of the endemic countries [2]. It is estimated that malaria accounts for 40% of public health expenditures, more than 30% of inpatient admissions, and approximately 50% of outpatient visits in some African countries [3]. These factors, as well as growing drug resistance of the parasite, widespread resistance of mosquitoes to insecticide, and increased human travel necessitate the need for new approaches to malaria control and eradication. A vaccine that could reduce both mortality and morbidity secondary to *P. falciparum* infection would be a valuable resource in the fight against this disease.

Over time, people living in endemic areas develop natural immunity to *P. falciparum* as a result of repeated infection. Consequently, children who survive to 5 years of age rarely succumb to life-threatening disease despite frequent infection. This acquired immunity is mediated in part by blood-stage parasite-specific antibodies [4]. Thus, parasite proteins expressed during the blood-stage have been proposed to be good candidates for inclusion in a vaccine. The purpose of an asexual blood-stage vaccine is to elicit immune responses that either destroy the parasite in the blood stream or inhibit the parasite from infecting red blood cells, thus reducing or preventing complications of the disease such as cerebral malaria, anemia, or renal failure.

A number of *P. falciparum* merozoite antigens have been identified as promising blood-stage vaccine candidates [4], including Merozoite Surface Protein 1 (MSP1) and Apical Membrane Antigen 1 (AMA1).

AMA1 PROTEIN

The *P. falciparum* Apical Membrane Antigen 1 (AMA1) gene product is an integral membrane protein that is synthesized as an 83 kDa precursor in merozoites [5]. At about the time of red blood cell invasion, the N-terminus of the 83 kDa precursor form is processed to a 66 kDa protein, and the resulting AMA1 is translocated to the merozoite cell surface [6].

The precise role of AMA1 in the parasite is unknown; however, it is critical in the erythrocyte invasion process across divergent *Plasmodium* species. AMA1 is critical for blood-stage multiplication of the parasite [7]. Recent analysis of the *P. falciparum* proteome detected expression of AMA1 in the sporozoite stage [8] and suggests an additional role for AMA1 during the liver-stage invasion [9]. Therefore, an immune response against AMA1 may have an effect on liver-stage parasites as well as having an effect on blood-stage parasites, thus protecting the host by multiple immune mechanisms. AMA1 is a target of protective immunity. Human and animal anti-AMA1 antibodies inhibit merozoite invasion in vitro [10-12] and correlate with protection against parasite challenge [13-15]. T-cells specific for *P. falciparum* AMA1 have also been demonstrated in individuals living in endemic areas [16-18].

At least 68 known amino acid polymorphisms of AMA1 have been demonstrated [19-21], and animal studies have shown that the polymorphisms in AMA1 are not immunologically silent

[10]. The combination of 2 or more divergent AMA1 sequences within a single vaccine formulation may reduce evasion of the host immune response by some *P. falciparum* field isolates.

$MSP1_{42}$ PROTEIN

The Merozoite Surface Protein 1 (MSP1) is synthesized as a ~200 kilodalton (kDa) polypeptide. MSP1 is processed, at or just prior to merozoite release from the red blood cell, into smaller fragments which form a non-covalently associated complex [22]. The 42 kDa C-terminal fragment of MSP1 ($MSP1_{42}$) is responsible for tethering the complex to the surface of the merozoite [23,24].

At the time of merozoite invasion of red blood cells, $MSP1_{42}$ undergoes a secondary processing event and is cleaved into $MSP1_{33}$ and $MSP1_{19}$ [25]. $MSP1_{19}$ is the only part of MSP1 carried into the newly invaded erythrocyte on the surface of the parasite [28]. This "secondary processing" of $MSP1_{42}$ is thought to be essential for parasite invasion of red blood cells as inhibition of this cleavage inhibits invasion and methods that prevent invasion also block cleavage [26].

In studies of natural infection, the majority of the B-cell epitopes have been localized to the highly conserved $MSP1_{19}$ region [27]. However, the T-cell epitopes which induce proliferation to $MSP1_{42}$ have been localized to the dimorphic region of the molecule, $MSP1_{33}$ [27,28]. This suggests that the development of an appropriate memory response following $MSP1_{42}$ vaccination requires the inclusion of the relevant T-helper epitopes from the dimorphic region as well as the conformation-dependent B epitopes.

Antibodies to the C-terminal region of $MSP1_{42}$ are associated with protection from high parasitemia and clinical disease [29]. Human anti-$MSP1_{19}$ antibodies have been shown to inhibit parasite multiplication in vitro [30,31]. Vaccination with $MSP1_{42}$-FVO, $MSP1_{42}$-3D7, or $MSP1_{42}$-C1 formulated with water-in-oil adjuvants induced antibodies which protect monkeys from experimental challenge with *P. falciparum* [32].

COMBINATION VACCINE

The aim of a combination vaccine (AMA1 and $MSP1_{42}$) is to avoid vaccine failure due to insufficient antibody responses in a given individual or antigenic diversity in parasite populations. In addition, it is believed there will be additive or synergistic consequences of targeting 2 merozoite surface proteins simultaneously. The result should be better immune coverage of an individual and the population, resulting in a vaccine with greater efficacy than either $MSP1_{42}$ or AMA1 used alone.

The native proteins of both AMA1 and $MSP1_{42}$ are polymorphic and the variants are thought to have emerged as a result of immunologic pressure [33,34]. Specificity of the antibody response could influence success or failure in a clinical situation. A vaccine composed of only 1 allelic form of either AMA1 or $MSP1_{42}$ may not be sufficient to protect against parasites carrying other variants of these proteins. A combination vaccine containing divergent polymorphic forms of these antigens would generate immune responses against 2 or more strains of the parasite and the polyclonal antibody response against different forms of these 2 proteins could provide appropriate protective epitopes to eliminate the majority of *P. falciparum* parasites. Vaccines that induce immunity against the most prevalent variant forms of the parasite would, in theory,

be most successful because they would diminish the negative impact polymorphism may have on vaccine efficacy, eliminate or reduce the likelihood for emergence of "break-through" parasites in the immunized individuals, and be less likely to result in rapid selection of vaccine-resistant parasite populations.

DESCRIPTION OF STUDY AGENT

1.1.1 AMA1-C1 Drug Substance

The AMA1-C1 component of BSAM-2/Alhydrogel® is an equal mixture of the correctly folded ectodomain portion of recombinant AMA1 analogous to 2 different lines of *P. falciparum* (FVO and 3D7). Recombinant AMA1-FVO and AMA1-3D7 proteins were separately expressed from synthetic DNA sequences in *Pichia pastoris*, and were then purified and characterized. Fermentation and purification of the 2 proteins are performed by procedurally similar batch production methods. The AMA1 proteins are supplied in phosphate buffered saline pH 7.4 without additional stabilizers or preservatives. This combination of AMA1 antigens was chosen because of concerns about parasite polymorphism, evidence for strain-specific protection as outlined above, and because their sequences are sufficiently different when comparing all sequenced *P. falciparum*. The intent is to provide a broad range of protection against the different strains of the parasite occurring in the field. Although relatively different among all known AMA1 sequences, the 2 recombinant proteins in the vaccine remain more than 95% homologous, with the cysteine residues in identical positions.

1.1.2 $MSP1_{42}$-C1 Drug Substance

Recombinant $MSP1_{42}$-FVO and $MSP1_{42}$-3D7 are highly purified proteins that correspond to the external domain of the *P. falciparum* $MSP1_{42}$ FVO and 3D7 lines, respectively. The proteins consist of the $MSP1_{42}$ fragment sequence excluding the glycosylphosphatidylinositol anchor sequence. The recombinant proteins $MSP1_{42}$-FVO and $MSP1_{42}$-3D7 were each expressed from synthetic genes in *Escherichia coli* and were purified from solubilized inclusion bodies by metal affinity chromatography. After refolding, the proteins were further purified by a combination of anion-exchange and size-exclusion chromatography. Both proteins were supplied in phosphate buffered saline pH 7.4 containing 0.2% polysorbate 80 without additional preservatives, and both underwent comprehensive quality control analysis to ensure purity, identity, and integrity. $MSP1_{42}$ is a dimorphic antigen and the proteins from the FVO and 3D7 *P. falciparum* parasite lines represent both known dimorphisms: $MSP1_{42}$-C1 is an equal mixture of the recombinant $MSP1_{42}$-FVO and $MSP1_{42}$-3D7 proteins. It is hoped that this combination will generate a protective immune response against epitopes found in all *P. falciparum* parasites.

1.1.3 BSAM-2

BSAM-2 contains a mixture of AMA1-C1 (AMA1-FVO+AMA1-3D7) and $MSP1_{42}$-C1 ($MSP1_{42}$-FVO+$MSP1_{42}$-3D7) as described above. $MSP1_{42}$-C1 and AMA1-C1 are mixed in a 1:1 ratio, based on antibody responses to the individual proteins in malaria naïve adults and results of stability studies. (BSAM-1 is a combination of the same proteins at higher doses, and is not being taken forward to clinical trials.)

1.1.4 Alhydrogel®

Alhydrogel®, an aluminum hydroxide gel (Brenntag, Denmark), has been extensively used as an adjuvant in many licensed human vaccines. Aluminum-containing adjuvants are in routine human use and are contained in many licensed human vaccines. Alhydrogel® is supplied as a sterile product in water without preservatives.

1.1.5 BSAM-2/Alhydrogel®

The BSAM-2/Alhydrogel® vaccine refers to BSAM-2 adsorbed to Alhydrogel®. BSAM-2/Alhydrogel® is supplied as a slightly turbid suspension in single-dose vials containing 1.0 mL. The vaccine Lot #L0811005 consists of 80 µg of total protein and 1600 µg of Alhydrogel® per 1.0 mL (low dose). The vaccine Lot #L0811007 consists of 320 µg of total protein and 1600 µg of Alhydrogel® per 1.0 mL (high dose). These products conform to the Code of Federal Regulations for endotoxin, sterility, and general safety.

1.1.6 CPG 7909

CPG 7909 (Coley Pharmaceutical Group, a Pfizer Company) is an investigational agent. CPG 7909 is a short synthetic oligodeoxynucleotide of the following sequence: 5'-TCG TCG TTT TGT CGT TTT GTC GTT-3', with all nucleotides linked with phosphorothioate bonds. CPG 7909 is manufactured according to current Good Manufacturing Practice (cGMP) standards. CPG 7909 Lot #L0808002 is packaged in sterile single dose vials of 0.08 mL at 10 mg/mL, formulated in a saline buffer for intramuscular (IM) administration. The product conforms to the Code of Federal Regulations for endotoxin and sterility. CPG 7909 is being studied extensively in humans as a cancer therapeutic agent and as a vaccine adjuvant.

PREVIOUS HUMAN EXPERIENCE

This Phase 1 study is the first time that the combination vaccine BSAM-2/Alhydrogel®+CPG 7909 will be given to humans. The components of the vaccine (both antigen and adjuvants) have been evaluated in prior clinical trials described below. Details on adverse events and immunogenicity results of these trials are described in the Investigators Brochure.

1.1.7 AMA1-C1/Alhydrogel®

There have been 4 human clinical trials to date using the recombinant AMA1-C1/Alhydrogel® vaccine.

The first clinical trial to use AMA1-C1/Alhydrogel® was performed at Johns Hopkins University (Baltimore, MD) [11]. Thirty subjects received up to 3 immunizations (Study Days 0, 28, and 180) with 5, 20, or 80 µg doses of AMA1-C1/Alhydrogel® in an open-label dose escalating study. No safety concerns were identified. Adverse events related to vaccination were limited to local injection site reactions, which were mild to moderate in severity. There was a dose response with the 80 µg group giving the highest antibody levels at Study Day 42 (14 days after the second vaccination) and at Study Day 194 (14 days after the third vaccination).

The second clinical trial using AMA1-C1/Alhydrogel® was started in Mali in May 2004. This study was a randomized, double blind, controlled trial [35]. Fifty-four healthy Malian adults were randomized 2:1 to receive either AMA1-C1/Alhydrogel® at 5, 20, or 80 µg doses, or the

Recombivax® Hepatitis B vaccine. Study participants received 3 immunizations on Study Days 0, 28, and 360. No vaccine-related serious or Grade 3 events were observed. AMA1 antibody responses to the 80 µg dose increased rapidly from baseline levels 14 and 28 days after the first vaccination and continued to increase after the second vaccination. Anti-AMA antibody levels peaked 14 days after the second vaccination then declined, reaching baseline levels 1 year later at the time of the third vaccination. Unlike the second vaccination, the third vaccination induced little or no increase in antibody levels.

The third trial was conducted at the NIH Clinical Center (Bethesda, MD). This study planned to vaccinate 12 healthy adults with 80 µg of AMA1-C1/Alhydrogel® and 6 with Alhydrogel® alone. Enrollment was closed at 6 volunteers due to difficulty with recruitment; 4 volunteers received AMA1-C1/Alhydrogel® and 2 received Alhydrogel®. The objectives of this study were to observe B-cell responses to vaccination. No safety concerns were identified with the 6 volunteers vaccinated.

The fourth trial was a Phase 1/2 randomized, controlled study of the safety and immunogenicity of AMA1-C1/Alhydrogel® in Malian children. Phase 1 of this trial was completed in May 2006, and Phase 2 completed vaccinations in September 2006. In Phase 1, 36 children were randomized to receive 2 doses of 20 µg AMA1-C1/Alhydrogel®, 80 µg AMA1-C1/Alhydrogel®, or the comparator vaccine (Hiberix®) [36]. In Phase 2, 300 children were vaccinated with either 80 µg AMA1-C1/Alhydrogel® or the comparator vaccine, and were followed through the malaria transmission season to assess biologic impact. No effect on frequency of parasitemia >3000/µL or on other measures of clinical malaria or parasite density was seen in the first transmission season (to Study Day 154). A statistically significant decrease in mean hemoglobin during clinical malaria episodes (9.95 g/dL vs 10.33 g/dL) and an increase in episodes of anemia (hemoglobin <8.5 g/dL, 0.75 episodes vs 0.48 episodes) were seen in the group who received AMA1-C1/Alhydrogel®, although these were not significant after correction was made for multiple tests. The original protocol planned to follow participants for 12 months after first vaccination. In the first year after the close of the study, 4 children had events which would have been considered serious adverse events, including 2 deaths. All were in the group which received AMA1-C1/Alhydrogel®. These events are described in the Investigator's Brochure. The protocol was subsequently reopened and additional safety data for the following transmission season was obtained. No significant differences in hemoglobin, frequency of anemia, or severity of malaria were detected in the extended follow up period.

1.1.8 AMA1-C1/Alhydrogel®+CPG 7909

A Phase 1 clinical trial of the recombinant AMA1-C1/Alhydrogel®+CPG 7909 vaccine was conducted at the University of Rochester (Rochester, NY) starting in May 2005 [37]. A total of 75 volunteers were enrolled, with 15 receiving 20 µg of AMA1-C1/Alhydrogel® + 500 µg CPG 7909 (phosphate), 30 receiving 80 µg AMA1-C1/Alhydrogel® + 500 µg CPG 7909 (phosphate) and 30 receiving 80 µg AMA1-C1/Alhydrogel®. Volunteers received up to 3 doses at Study Days 0, 28, and 56. The aim of this study was to test both the safety of the AMA1-C1/Alhydrogel®+CPG 7909 vaccine, and to compare its immunogenicity with the AMA1-C1/Alhydrogel® formulation. The vaccine was well tolerated, with pain at the injection site being the most commonly observed reaction. Anti-AMA1 immunoglobulins G (IgG) were detected by enzyme-linked immunosorbent assay (ELISA), and the immune sera of volunteers

that received 20 µg or 80 µg of AMA1-C1/Alhydrogel® + CPG 7909 had up to 14-fold significant increases in anti-AMA1 antibody concentration compared to 80 µg of AMA1-C1/Alhydrogel® alone. The addition of CPG 7909 to the AMA1-C1/Alhydrogel vaccine elicited AMA1-specific immune IgG with in vitro growth inhibition of homologous parasites as high as 96%. Antibody levels were the same following the second and third doses in volunteers receiving CPG 7909, indicating no additional boosting with the third dose.

1.1.9 AMA1-C1/Alhydrogel®+CPG 7909 (phosphate and saline)

The studies described in **Sections 1.6.2** (above) and **1.6.5** and **1.6.6** (below) used CPG 7909 in a phosphate buffer. Studies at the Laboratory of Malaria Immunology and Vaccinology (LMIV), formerly the Malaria Vaccine Development Branch, indicate that binding of the AMA1-C1 protein to Alhydrogel® is optimized when the CPG 7909 is formulated in a saline buffer. A Phase 1 study of AMA1-C1 with CPG 7909 in a saline buffer was conducted by the Center for Immunization Research to evaluate safety and immunogenicity of the saline formulation, and to demonstrate that the addition of CPG 7909 in saline enhanced the immune response to AMA1-C1 in a manner similar to that seen with the addition of CPG 7909 in phosphate. Twenty-four (24) volunteers were enrolled and received 2 vaccinations of either AMA1-C1/Alhydrogel®+CPG 7909 (phosphate) or AMA1-C1/Alhydrogel®+CPG 7909 (saline), on a 0,1 or 0,2 month schedule in a 2x2 design. No safety issues were identified. Responses were similar in the groups receiving the phosphate and saline formulations, and were higher in the groups receiving vaccinations on a 2 month rather than 1 month schedule.

A Phase 1 study in malaria-exposed adults in Mali completed vaccinations in December, 2007 and follow up was completed in May 2008. Twenty-four (24) volunteers were enrolled and randomized 1:1 to receive either AMA1-C1/Alhydrogel® or AMA1-C1/Alhydrogel ®+500 µg CPG 7909. Two vaccinations were given 1 month apart. One volunteer was withdrawn from vaccinations due to a Grade 1 hypersensitivity reaction; this volunteer had a previously undisclosed history of allergic reactions requiring treatment with steroids. No other safety issues were identified and both vaccines were well tolerated. A ~2.5-fold increase in peak antibody responses was seen in volunteers receiving CPG 7909.

1.1.10 $MSP1_{42}$/Alhydrogel®

A Phase 1 study of $MSP1_{42}$-FVO and $MSP1_{42}$-3D7 proteins, separately adjuvanted with Alhydrogel® was conducted at Quintiles Phase 1 Services (Lenexa, KS) between July 2004 and November 2005 [38]. Sixty (60) volunteers were enrolled and received up to 3 doses of vaccine on Study Days 0, 28, and 180. Ten volunteers were assigned to each of 3 dose cohorts (5, 20, and 80 µg) for each vaccine for a total of 6 groups (3 groups for $MSP1_{42}$-FVO/Alhydrogel® and 3 groups for $MSP1_{42}$-3D7/Alhydrogel®). One volunteer in the 80 µg $MSP1_{42}$-3D7/Alhydrogel® dose group experienced an atypical injection site reaction with musculoskeletal pain; all other local reactions were mild or moderate. No serious adverse events occurred that were related to the vaccines. Anti-$MSP1_{42}$ antibodies were detected by enzyme-linked immunosorbent assay (ELISA) in 20/27 (74%) and 22/27 (81%) of volunteers receiving 3 vaccinations. Antibodies were cross-reactive to both proteins. However, T-cell responses were specific for the immunizing form of the protein [39]. Immunofluorescence microscopy demonstrated reactivity with FVO and 3D7 schizonts and free merozoites; minimal *in vitro* growth inhibition was observed.

1.1.11 $MSP1_{42}$-C1/Alhydrogel®+ CPG 7909

A Phase 1 study in malaria-naïve adults of $MSP1_{42}$-C1/Alhydrogel®, with and without CPG 7909 (phosphate), was conducted at the Center for Immunization Research. The study enrolled 30 volunteers in the low dose cohort (40 µg $MSP1_{42}$-C1 with and without 500 µg CPG 7909), and 30 volunteers in the high dose cohort (160 µg $MSP1_{42}$-C1 with and without 500 µg CPG 7909). Volunteers received up to 3 doses on Study Days 0, 28, and 56. No safety issues were identified. Antibody responses in the $MSP1_{42}$-C1/Alhydrogel®+ CPG 7909 groups were higher than in the $MSP1_{42}$-C1/Alhydrogel® groups. *In vitro* parasite growth inhibition was demonstrated, with an average of 14% (range 3-32%) in the 160 µg $MSP1_{42}$-C1/Alhydrogel®+CPG dose group. As with AMA1-C1/Alhydrogel®+CPG 7909, no additional boosting of antibody titers was seen with the third dose in groups receiving CPG 7909.

1.1.12 Clinical Experience with BSAM-2/Alhydrogel+CPG 7909 to date

The Phase 1 study of BSAM-2/Alhydrogel®+CPG 7909 described in this protocol began vaccinations at the Center for Immunization Research, Washington, DC, in June, 2009. As of September 30, 2009, a total of 30 malaria naïve US volunteers have been enrolled to receive up to 3 doses of either 40 or 160 µg BSAM-2 with 560 µg CPG 7909. Local adverse events have been as expected, with mild or moderate injection site pain. Systemic adverse events after first or second vaccination have reached Grade 3 severity in 4 volunteers (Grade 3 malaise in 2 volunteers, and Grade 3 fever and headache in 2 additional volunteers). Two volunteers have been withdrawn from vaccinations due to these adverse events. The events typically occur within 24 hours of vaccination and resolve within several hours of administration of acetaminophen or ibuprofen. Transient mild or moderate neutropenia has also been seen, as in previous studies with CPG 7909.

1.1.13 Previous Clinical Experience with CPG 7909 (VaxImmune®)

CPG 7909 (in a phosphate buffer) has been administered to humans in combination with Engerix-B® Hepatitis B vaccine (in both healthy and human immunodeficiency virus [HIV]-infected adults), Fluarix® killed influenza vaccine, and anthrax vaccine adsorbed (BioThrax®) [40-43]. Initial results from these clinical trials indicate that the addition of CPG 7909 is safe, and in the case of Engerix-B® and BioThrax®, induces substantially earlier and stronger antibody responses than the vaccine alone.

The Engerix-B® trial in healthy adults was a randomized, double-blind study in 56 subjects in Canada [41]. This was a dose escalating study comparing 0.125, 0.5, and 1.0 mg of CPG 7909 admixed with the licensed Engerix-B® vaccine. The most frequently reported adverse events were injection site reactions (pain and erythema), flu-like symptoms, and headache. All adverse events were mild or moderate in severity. One volunteer experienced a hypersensitivity-type reaction immediately following the third dose of Engerix-B® + 1.0 mg of CPG 7909. The symptoms included warmth, weakness, nausea, and dizziness. The symptoms were transient and resolved without treatment. One volunteer who received Engerix-B® + 1.0 mg of CPG 7909 had moderate elevations in anti-dsDNA which was initially detected 2 weeks after the second and third vaccinations. The anti-dsDNA returned to normal prior to receipt of the third dose and was normal at the end of the study. The volunteer was asymptomatic, and antinuclear antibody and rheumatoid factor remained negative throughout.

Engerix-B® mixed with CPG 7909 was also evaluated in a randomized, double-blind controlled trial in 58 HIV-infected adults in Canada [42]. The most common adverse events were local injection site reactions and influenza-like symptoms. Serious adverse events were reported in 2 individuals who received Engerix-B® with CPG 7909: 1 who developed unstable angina and myocardial infarction 2 and 4 months after the last vaccination respectively, and 1 who had laparoscopy for chronic pelvic inflammatory disease 16 days following vaccination. Laboratory abnormalities included transient declines in total lymphocytes and CD4 cells. Two subjects receiving Engerix-B® plus CPG 7909 and 2 subjects receiving CPG 7909 alone had transient elevations of anti-dsDNA; there were no clinical findings of autoimmunity.

The Fluarix® trial was a randomized, controlled, double-blind study in 60 healthy subjects in Canada [40]. Subjects received either the licensed Fluarix® vaccine with or without CPG 7909 or 1/10th the dose of Fluarix® with or without CPG 7909. The most frequently reported adverse events were injection site pain, headache, myalgia, and fatigue. All injection site reactions were mild or moderate in severity, with the exception of 1 volunteer in the 1/10th dose Fluarix® + CPG 7909 group who reported severe pain which resolved without treatment within 4 days. Transient reductions in total white blood cells (WBC), neutrophils, lymphocytes, eosinophils, and platelets were observed in all 4 study arms 2 days post-vaccination, with larger reductions in WBC in the CPG 7909 groups. None of these results were felt to be clinically significant.

CPG 7909 combined with anthrax vaccine absorbed (AVA; licensed as BioThrax®), was evaluated in a randomized, controlled, double-blind study in 69 healthy adults [43]. Local injection site and systemic symptoms were expected and were the most common adverse events. There was a trend to greater frequency and severity of adverse events in the AVA plus CPG 7909 group compared to the AVA alone and CPG 7909 alone groups but this was not statistically significant. Grade 1 leukopenia and hypokalemia were seen in all study arms.

1.1.14 Clinical Development Plan

Should safety be demonstrated in US and Malian adults, and immunogenicity demonstrated in US adults, BSAM-2/Alhydrogel®+CPG 7909 (or another closely related CPG adjuvant) will be evaluated for safety and immunogenicity in Malian children. If the vaccine is found to be safe and immunogenic in this population, a Phase 2 study in Malian children is anticipated. Options for demonstrating benefit as well as safety in adults prior to studies in children are also being explored.

2. STUDY OBJECTIVES

PRIMARY OBJECTIVE

The primary objective of this study is to assess safety and reactogenicity of BSAM-2/Alhydrogel®+CPG 7909 in malaria-naïve US adults and semi-immune Malian adults.

SECONDARY OBJECTIVES

The secondary objective of this study is to determine the antibody response of the combination vaccine to the AMA1 and $MSP1_{42}$ proteins, as measured by antibody levels and parasite growth inhibition assay.

EXPLORATORY OBJECTIVES

Exploratory objective of this study include the following:

- To determine the extent to which the antibody response to the individual antigens (AMA1 and $MSP1_{42}$) is correlated when the combination vaccine (BSAM-2/Alhydrogel®+CPG 7909) is given.
- To determine T and B cell responses to vaccination.

3. STUDY DESIGN

OVERALL DESIGN

This is a dose escalating multi-site Phase 1 clinical trial of the blood stage malaria vaccine candidate BSAM-2/Alhydrogel®+CPG 7909. Dose escalation will occur in the first part of the study, conducted in malaria-naïve adults at the Johns Hopkins Center for Immunization Research (CIR), in Washington, DC. Fifteen (15) participants will receive 40 µg BSAM-2/Alhydrogel® + 500 µg CPG 7909 (saline), with a group of 5 vaccinated first, followed approximately 2 weeks later by a group of 10. Providing halting rules are not met, an additional 15 participants will receive 160 µg BSAM-2/Alhydrogel® + 500 µg CPG 7909 (saline), also on a similar staggered schedule. All participants will receive 3 doses of vaccine administered IM, with the first 2 doses approximately 2 months apart and the third dose 6 months after the first. After the Safety Monitoring Committee reviews the safety data (through Study Day 63) of the last group to be vaccinated in the US, the second part of the study will be conducted in semi-immune adults in Bancoumana, Mali. This part of the study will be single-blinded and will include a comparator group. The comparator group in Mali will allow for better interpretation of antibody response in a population which has pre-existing immunity, particularly antibodies to the vaccine antigens, and which may experience malaria infection during the vaccination period, which would also affect antibody levels. Use of a comparator vaccine and blinding will also allow for more objective assessment of adverse events in this population, which has a higher rate of febrile illness and other clinical events that could be perceived as related to vaccination. Thirty (30) participants in Mali will be randomized 1:1 to receive the highest safe dose of BSAM-2/Alhydrogel®+CPG 7909, as determined at the SMC review, or the comparator vaccine (Euvax B/ Hepatitis B Vaccine, Recombinant), on a staggered schedule as in the US. The study vaccination schedule is outlined in **Table 2** below.

Table 2: Vaccination Schedule

Part 1: CIR/Malaria-naïve US adults (30 participants total)		
Week*	Vaccination 1	Vaccination 2
0	**5**: 40 µg BSAM-2/Alhydrogel® + 500 µg CPG 7909	
2	**10**: 40 µg BSAM-2/Alhydrogel® + 500 µg CPG 7909	
4	**5**: 160 µg BSAM-2/Alhydrogel® + 500 µg CPG 7909	
6	**10**: 160 µg BSAM-2/Alhydrogel® + 500 µg CPG 7909	
8		**5**: 40 µg BSAM-2/Alhydrogel® + 500 µg CPG 7909
10		**10**: 40 µg BSAM-2/Alhydrogel® + 500 µg CPG 7909
12		**5**: 160 µg BSAM-2/Alhydrogel® + 500 µg CPG 7909
14		**10**: 160 µg BSAM-2/Alhydrogel® + 500 µg CPG 7909
16	Safety data analysis	
18	SMC Review of safety data to Day 63 (SMC to include Malian representative)	
26-32	40 or 160 µg BSAM-2/Alhydrogel® + 500 µg CPG 7909 (Vaccination 3)	
**Part 2: University of Bamako/Semi-immune Malian adults (30 participants)**		
0 (following SMC Review)	**5**: 160** µg BSAM-2/Alhydrogel® + 500 µg CPG 7909<br>**5**: Euvax B/Hepatitis B Vaccine,	
2	**10**: 160 µg BSAM-2/Alhydrogel® + 500 µg CPG 7909<br>**10**: Euvax B/Hepatitis B Vaccine	
4		
6		
8		**5**: 160 µg BSAM-2/Alhydrogel® + 500 µg CPG 7909<br>**5**: Euvax B/ Hepatitis B Vaccine
10		**10**: 160 µg BSAM-2/Alhydrogel® + 500 µg CPG 7909<br>**10**: Euvax B/ Hepatitis B Vaccine
17-19	160 µg BSAM-2/Alhydrogel® + 500 µg CPG 7909 or Euvax B/ Hepatitis B Vaccine (Vaccination 3)	

*Schedule is approximate; a minimum of 2 weeks will separate the last participants vaccinated at the lower dose level from the first vaccinated at the higher dose level.
**Here and elsewhere, it is assumed that the dose administered in Mali will be 160 µg BSAM-2/Alhydrogel®+CPG 7909. Should safety issues at the higher dose be identified at the SMC review, the lower dose may be used.

Participants in Mali will be told of their vaccination status at completion of the study. At that time, participants who received BSAM-2/Alhydrogel®+CPG 7909 may start the 3 dose Euvax B/ Hepatitis B Vaccine series if they wish to do so.

STUDY ENDPOINTS

3.1.1 Primary Endpoints

The incidence and severity of systemic and local adverse events

3.1.2 Secondary Endpoints

1. Antibody response at selected time-points elicited by BSAM-2/Alhydrogel®+CPG 7909 to AMA1 and $MSP1_{42}$ proteins, analogous to the FVO and 3D7 *P. falciparum* proteins, will be determined by ELISA.
2. Inhibition of *P. falciparum* FVO and 3D7 parasite growth *in vitro* will be determined at selected time-points, as well as the extent to which anti-AMA1 or anti-$MSP1_{42}$ antibody levels correlate with growth inhibition.

3.1.3 Exploratory Endpoints

1. T-cell responses will be measured by cytokine levels and ELISPOT assays.
2. The number and proportion of AMA1 and $MSP1_{42}$ specific memory B cells will be measured by flow cytometry and ELISPOT assays.

Sample Size and Estimated Duration of Study

A total of 60 participants will be vaccinated in this trial, 30 in the US, and 30 in Mali. Each participant will be monitored actively for approximately 52 weeks from the time of the first injection, with additional scheduled follow up 18 months after the last vaccination. In the US this additional visit or phone call will be optional. In Mali passive follow up will continue until this last visit occurs. Up to 200 participants will be screened in the US and up to 200 will be screened in Mali to accommodate possible screening failures.

4. STUDY POPULATION

Description of Population and Site

4.1.1 US

The US part of the study will be conducted at the CIR in Washington, DC. This group has successfully conducted 4 previous Phase 1 studies of malaria vaccine candidates in collaboration with LMIV. Malaria-naïve volunteers will be recruited from the surrounding community through a screening protocol as described in **Section 4.2**.

4.1.2 Mali

The study will be conducted by the Malaria Research and Training Center (MRTC), University of Bamako in Bancoumana, a village in Mali. Bancoumana is located 60 km southwest of Bamako and has a population of about 8,000 people. The site is situated in the Sudanian area of Mali. The climate is hot, with daily temperatures ranging from 19°C to 40°C. The annual rainfall varies between 600 mm and 1200 mm and occurs from June to October.

Epidemiological malaria surveys have been done in Bancoumana [44,45]. These data show that Bancoumana is a suitable village for conducting Phase 1 and Phase 2 malaria vaccine trials. The MRTC has established a clinical trial center inside the village health center. Because of the excellent trial infrastructure and community involvement, Bancoumana has been chosen as the site for this study.

Recruitment: US

Healthy adult male and nonpregnant female volunteers will be recruited from a variety of sources

including those previously screened or enrolled in other vaccine trials at the CIR, or by the use of an IRB-approved screening protocol and study-specific print or media advertising. Clinical trial site staff will contact prospective volunteers by telephone to impart background information concerning the trial and to review basic inclusion and exclusion criteria. If prospective candidates seem qualified for the trial, they will be scheduled for a screening visit.

During the initial screening visit, the volunteer will read the consent form, be encouraged to ask questions, and then complete a comprehension evaluation questionnaire (**Appendix A**). The questionnaire is used to identify the areas of the study and consent that the volunteer may not fully understand. In the US the evaluation is done by the volunteer in a written format. The person administering consent then reviews the initial answers with the volunteer. If the volunteer gets a question wrong, the person administering the consent reviews the portion of the consent form that relates to that particular question with the volunteer. If after this process the volunteer is not able to answer all questions correctly he or she will not be enrolled in the study. The volunteer may either sign the consent form during the screening visit, or return after further consideration.

RECRUITMENT: MALI

4.1.3 Community Consent

During previous studies conducted by the MRTC in Bancoumana, residents have become familiar with the informed consent process, including the need for written, signed consent forms.

The community consent process goes through the following steps:

i. Study personnel explain the study to village leaders, including the village chief and elders.
ii. The village leaders then discuss the study with community members and relay any additional questions or concerns they may have to study personnel.
iii. The study is explained to heads of families by study personnel.

At the time of community consent, the need for both husband and wife to agree to avoid pregnancy for the specified period if a wife chooses to volunteer will also be addressed.

The informed consent will be translated into French. The study team conducts careful word-for-word review of the study consent form, and will translate the consent orally into local languages, as the majority of potential study volunteers do not read or speak French. (Written versions of the local languages do not exist, so a written translation is not possible.) Verification that the oral translations are accurate and that the potential participants understand the contents of the informed consent form will be done by an independent witness chosen by the participant, and is not a member of the study team.

4.1.4 Individual Recruitment and Informed Consent

Potential volunteers in the village will be invited through a general announcement to come to the study clinic for informed consent and screening. During this initial visit, the volunteer will read the consent form or have it explained to him/her in cases of illiteracy. Volunteers will be encouraged to ask questions, and then take a multiple-choice questionnaire to evaluate consent comprehension (**Appendix A**); the comprehension document will be translated into French and

administered orally in their native dialect in the case of potential volunteers who cannot read. As in the US, the volunteer must answer all questions correctly prior to being eligible for enrollment. Study staff will use incorrect answers from the questionnaire to identify those areas of the informed consent form that need further review with the volunteer. This will help ensure the volunteer has sufficient understanding before the consent form is signed. The volunteer may either sign the consent form immediately or later after further consideration. Volunteers unable to read will place a fingerprint in the place of a signature; in addition, an independent witness will sign the consent form to attest that the volunteer fully comprehended the contents.

INCLUSION CRITERIA

All of the following criteria must be fulfilled for a volunteer to participate in this trial:

- Age between 18 and 50 years (US) or 18 and 45 years (Mali), inclusive
- Good general health as a result of review of medical history and/or clinical testing at the time of screening
- Available for the duration of the trial (52 weeks in the US, and 104 weeks in Mali)
- Willingness to participate in the study as evidenced by signing the informed consent document, or by fingerprinting the consent document with the signature of a witness (Mali)
- Known resident of the village of Bancoumana (Mali)

EXCLUSION CRITERIA (US)

A volunteer will be excluded from participating in this trial if any one of the following criteria is fulfilled:

1. Pregnancy as determined by a positive urine or serum test at any point during the study for human choriogonadotropin (β-hCG) (if female).
2. Subject is unwilling to use reliable contraception methods for the period of at least 2 months prior to first vaccination to 3 months after last vaccination (if female). Reliable methods of birth control include: pharmacologic contraceptives including oral, parenteral, and transcutaneous delivery; condoms with spermicide; diaphragm with spermicide; surgical sterilization; vaginal ring; intrauterine device; abstinence; and post-menopause (if female).
3. Currently is lactating and breast-feeding (if female).
4. Behavioral, cognitive, or psychiatric disease that in the opinion of the investigator affects the ability of the participant to understand and cooperate with the study protocol.
5. Neutropenia as defined by an absolute neutrophil count $<1500/mm^3$.
6. Alanine transaminase (ALT) level above the laboratory-defined upper limit of normal.
7. Evidence of clinically significant neurologic, cardiac, pulmonary, hepatic, endocrine, rheumatologic, autoimmune, or renal disease by history, physical examination, and/or laboratory studies including urinalysis.
8. Other condition that in the opinion of the investigator would jeopardize the safety or rights of a participant participating in the trial or would render the subject unable to comply with the protocol.
9. History of receiving any investigational product within the past 30 days.

10. Participant has had medical, occupational, or family problems as a result of alcohol or illicit drug use during the past 12 months.
11. History of a severe allergic reaction or anaphylaxis.
12. Severe asthma. This will be defined as:
    - Asthma that is unstable or required emergent care, urgent care, hospitalization or intubation during the past 2 years, or that requires the use of oral or parenteral corticosteroids
    - Clinically significant reactive airway disease that does not respond to bronchodilators
13. Positive ELISA and confirmatory Western blot tests for HIV-1.
14. Positive ELISA and confirmatory tests for hepatitis C virus (HCV).
15. Positive hepatitis B surface antigen (HBsAg) by ELISA.
16. Pre-existing autoimmune or antibody-mediated diseases including but not limited to: systemic lupus erythematosus, rheumatoid arthritis, multiple sclerosis, Sjögren's syndrome, or autoimmune thrombocytopenia.
17. Known immunodeficiency syndrome.
18. Positive serum anti-dsDNA titer.
19. Use of corticosteroids (excluding topical or nasal) or immunosuppressive drugs within 30 days of starting this study.
20. Receipt of a live vaccine within past 4 weeks or a killed vaccine within past 2 weeks prior to entry into the study.
21. History of a surgical splenectomy.
22. Receipt of blood products within the past 6 months.
23. Previous receipt of an investigational malaria vaccine.
24. Receipt of antimalarial prophylaxis during the past 12 months, or receipt of chloroquine or related compounds (hydroxychloroquine, amodiaquine, or primaquine) in the 8 weeks prior to study entry.
25. Prior malaria infection by history.
26. Any medical, psychiatric, social, or occupational condition or other responsibility that, in the judgment of the Principal Investigator (PI), would interfere with the evaluation of study objectives.

EXCLUSION CRITERIA (MALI)

A volunteer will be excluded from participating in this trial if any one of the following criteria is fulfilled:

1. Pregnancy as determined by a positive urine β-hCG test at any point during the study (if female).
2. If female, subject and her spouse have not used or are unwilling to use reliable contraceptive methods such as: abstinence, birth control pills or birth control patches or vaginal ring, diaphragm with spermicide, IUD (intrauterine device), condom with spermicide, progestin implant or injection, or surgical sterilization (hysterectomy, bilateral oophorectomy, tubal ligation) prior to enrollment to 3 months after the final vaccination. (At the time of vaccination, a female subject must have had a negative urine pregnancy test on 2 occasions at least 2 weeks apart, and must have used a reliable contraceptive method in the interim.)
3. Currently is lactating and breast-feeding (if female).

4. Evidence of clinically significant neurologic, cardiac, pulmonary, hepatic, rheumatologic, chronic infectious or renal disease by history, physical examination, and/or laboratory studies including urinalysis.
5. Behavioral, cognitive, or psychiatric disease that in the opinion of the investigator affects the ability of the subject to understand and cooperate with the study protocol.
6. Pre-existing known autoimmune diseases including but not limited to: systemic lupus erythematosus, rheumatoid arthritis, multiple sclerosis, Sjögren's syndrome, autoimmune thrombocytopenia.
7. Laboratory evidence of possible autoimmune disease determined by anti-dsDNA titer that equals or exceeds 25 IU.
8. Laboratory evidence of liver disease (alanine aminotransferase [ALT] greater than 1.25 times the upper limit of normal of the testing laboratory).
9. Laboratory evidence of renal disease (serum creatinine greater than the upper limit of normal of the testing laboratory, or more than trace protein or blood on urine dipstick testing confirmed by repeat testing of clean-catch, midstream sample). (More than trace blood on urine dipstick will not exclude a female who is actively menstruating.)
10. Laboratory evidence of hematologic disease (absolute leukocyte count $<3000/mm^3$ or $>11,500/mm^3$; hemoglobin <0.9 times the lower limit of normal of the testing laboratory, by gender; absolute granulocyte count $<1300/mm^3$; absolute lymphocyte count $<1000/mm^3$; or platelet count $<110,000/mm^3$).
11. Other condition that, in the opinion of the investigator, would jeopardize the safety or rights of a volunteer participating in the trial or would render the subject unable to comply with the protocol.
12. Participation in another investigational vaccine or drug trial within 30 days of starting this study, or while this study is ongoing.
13. Volunteer has had medical, occupational, or family problems as a result of alcohol or illicit drug use during the past 12 months.
14. History of a severe allergic reaction or anaphylaxis.
15. Severe asthma. This will be defined as:
    - Asthma that is unstable or required emergent care, urgent care, hospitalization or intubation during the past 2 years, or that requires the use of oral or parenteral corticosteroids
    - Clinically significant reactive airway disease that does not respond to bronchodilators
16. Positive hepatitis B surface antigen (HBsAg). Hepatitis C antibody by rapid diagnostic test.
17. Known immunodeficiency syndrome.
18. Use of systemic corticosteroids (excluding topical or nasal) or immunosuppressive drugs within 30 days of starting this study.
19. Receipt of a live vaccine within past 4 weeks or a non-live vaccine within past 2 weeks prior to entry into the study.
20. History of a surgical splenectomy.
21. Receipt of blood products within the past 6 months.
22. Previous receipt of an investigational malaria vaccine.
23. History of use of chloroquine or related compounds (hydroxychloroquine, amodiaquine, or primaquine) within 8 weeks of study entry.

Rationale for Different Inclusion/Exclusion Criteria in US and Mali Sites

The age range for inclusion is slightly narrower in Mali than in the US. This is because nutritional and overall health status for Malian adults is in general poorer than in the US. Inclusion of slightly younger participants in Mali may help to correct for this. Previous Phase 1 studies in Mali and in the US have included adults within the age ranges specified here. Laboratory exclusion criteria are slightly different in the 2 sites to account for differences in the normal values in these populations, and also because the MRTC clinical laboratory is being used rather than a commercial laboratory as in the US. Screening for hepatitis B and C will be performed using rapid diagnostic tests in Mali. In addition to a history of severe allergic reaction or anaphylaxis, severe asthma is an exclusion criterion in Mali because high level medical care is not available in the village and, in the event of a worsening of asthma due a vaccine reaction or other illness, such care would not be available. This is not the case in the US.

We do not plan to test for HIV at the time of screening in Mali. This malaria vaccine (BSAM-2/Alhydrogel®+CPG 7909) as well as the control vaccine (Euvax B/Hepatitis B vaccine) are both killed vaccines (a part of the germ) and not attenuated vaccines; therefore, there is little or no risk for aggravating the respective disease for which the vaccine is given. Also, the HIV seroprevalence is 1.3% in Mali, one of the lowest rates in sub-Saharan Africa. Although no serosurveys have been done in Bancoumana itself, this site is in a rural area and is likely to have a prevalence rate not greater than the average for the entire country; therefore, there is little chance to find positive HIV cases among the 30 enrolled subjects. Thus, HIV screening is not necessary in this study.

5. STUDY AGENTS

Vaccine

The BSAM-2/Alhydrogel® vaccine and CPG 7909 will be supplied to the study site pharmacist by the Biopharmaceutical Development Program, SAIC-Frederick, Inc., National Cancer Institute at Frederick, MD, where the materials were formulated and packaged. Each of the 2 components will be supplied separately in single-use vials containing 1 mL of 80 µg/mL BSAM-2/Alhydrogel® (Lot #L0811005), 1 mL of 320 µg/mL BSAM-2/Alhydrogel® (Lot #L0811007), or 0.08 mL of 10 mg/mL CPG 7909 (Lot #L0808002), and labeled for investigational use only.

Euvax B/Hepatitis B Vaccine

Euvax B consists of highly purified, non infectious particles of Hepatitis B surface antigen (HBsAG) absorbed onto aluminum salts as an adjuvant and preserved with thimerosal. It is a recombinant DNA hepatitis B vaccine derived from HBsAg produced by DNA recombinant technology in yeast cells (*Saccharomyces cerevisiae*). Other excipients are potassium phosphate monobasic, sodium phosphate dibasic and sodium chloride. Euvax B is for intramuscular use only. The vaccine is manufactured by LG Life Sciences, based in the Republic of Korea. The vaccine meets the WHO requirements for recombinant hepatitis B vaccines, and is widely used and available in Mali. No substances of human origin are used in its manufacture. While the recommended vaccination schedule is for 0, 1, and 6 months, per the Centers for Disease Control, recombinant hepatitis B vaccines may be administered on a 0, 1, and 4 or 0, 2, and 4 month schedule with dose-specific and final rates of seroprotection similar to those obtained on a

0, 1, and 6 month schedule. (MMWR Recommendations and Reports, December 8, 2006 / 55(RR16);1-25).

VACCINE ADMINISTRATION

The trial site pharmacy will prepare the point-of-injection formulation. Shortly before vaccination, 0.7 mL of BSAM-2/Alhydrogel® is withdrawn and added to the single dose vial containing 0.08 mL CPG 7909. The vial is then mixed. This formulation is referred to as BSAM-2/Alhydrogel®+CPG 7909. The vaccine must be administered not more than 6 hours after mixing the BSAM-2/Alhydrogel® with the CPG 7909 and should be stored at 2-8°C if not immediately administered. When ready to administer BSAM-2/Alhydrogel®+CPG 7909, 0.55 mL is withdrawn into a syringe and the vaccine is injected. A 0.55 mL dose of the vaccine corresponds to 500 µg of CPG 7909 and either 40 µg (low dose) or 160 µg (high dose) of BSAM-2, depending on the dose of BSAM-2/Alhydrogel®. (The actual dose of CPG 7909 will be 564 µg but is shown here and elsewhere as 500 µg for simplicity. The actual antigen dose in 0.55 mL is 39.5 µg (low dose) and 158.0 µg (high dose.)

VACCINE STORAGE

BSAM-2/Alhydrogel® vaccine, CPG 7909, and Euvax B/Hepatitis B vaccine will be transported and stored at temperature-controlled conditions, as per SOP. Temperature data loggers will accompany the vaccines at all times to ensure storage temperature limits have not been violated. Vaccines and adjuvant should NOT be frozen at any time. Refrigerator temperature will be continuously monitored.

VACCINE ACCOUNTABILITY

5.1.1 Disposition

After administration of a vaccine dose, the single-dose vial will be returned to the Pharmacy at the test site, and vials will be accounted for and stored until monitoring by the IND Sponsor. The used vials may then be disposed of according to site standard operating procedures after monitoring has been completed.

5.1.2 Documentation

The trial site pharmacist is responsible for maintaining an accurate inventory and accountability record of vaccine supplies for this study. Partially used vials may not be administered to other subjects.

6. STUDY PROCEDURES

SCREENING

The purpose of the screening visit is to determine volunteer eligibility for study participation. Subjects in the US who are diagnosed with a medical condition during the screening process (e.g., test positive for hepatitis B, hepatitis C, or HIV) will be notified and referred for medical care. In Mali, any clinically relevant finding that is discovered upon screening will be treated appropriately according to the standard of care as follows: initial management will be performed at the study clinic, free of charge. Should referral for more extensive investigation or treatment be required, the study will arrange and pay for transportation to a National Hospital and initial

consultation. Initial care according to the standard of care in Mali will be covered by the study; however, in the event that a chronic illness is discovered during the course of screening, long-term treatment and care will not be reimbursed by the study.

The following screening evaluation for this study must be completed within the 60 days prior to vaccination.

- Explain the study and Informed Consent to the subject
- Ensure the subject has signed the Informed Consent and receives a signed copy of the Informed Consent, and has correctly answered all questions on the Informed Consent Comprehensive Exam
- Explain the HIV Informed Consent and obtain a signed copy from the subject (US only)
- Elicit a complete medical history, including menstrual and contraceptive history and/or history of surgical sterility for females
- Administer a complete physical examination
- Obtain a signed medical release of information form from the patient (US only)

Obtain approximately 25 mL of blood for complete blood count (CBC) with differential and platelet count, ALT, creatinine, anti-dsDNA, hepatitis B surface antigen, hepatitis C antibody, HIV antibody (US only) and malaria smear and filter paper blood collection (Mali only) in all subjects. Obtain urine for pregnancy testing and dipstick testing for protein and blood.

ASSIGNMENT TO GROUPS

Subjects in the US will be assigned to dose groups in the order in which they are successfully screened. The Mali phase of the trial will be randomized and subjects will be blinded. Clinical investigators and other personnel in Mali conducting screening will also be blinded to the randomization code.

BLINDING AND RANDOMIZATION (MALI)

Vaccinations in Mali will be single-blinded, with half of the subjects receiving the comparator vaccine, Euvax B/ Hepatitis B Vaccine. The comparison group will allow for better interpretation of antibody levels in a population which has pre-existing antibody titers to the vaccine antigens, and which may experience malaria infection during the vaccination period, which would also affect antibody titers. Blinding will also allow for more objective assessment of adverse events in this population, which has a higher rate of febrile illness and other clinical events which could be perceived as related to vaccination. The study pharmacist will be aware of the vaccination assignment (BSAM-2/Alhydrogel®+CPG 7909 versus Euvax B/ Hepatitis B Vaccine), but subjects and clinical investigators responsible for assessment of adverse events will not be aware of vaccination assignment until the completion of the study. Syringes will be covered by a sleeve or opaque tape to mask their appearance and preserve blinding. In addition, vaccines will be administered by investigators who are not involved in assessment of adverse events. The senior investigators in Mali may choose to remain blinded, but will have access to the study codes if necessary. Randomization codes and labels will be generated by the study statistician, and will be used by the study pharmacist as per SOP. The study numbers will be assigned in the order in which participants are vaccinated.

Immunization Procedure

The study schedule and approximate amounts of blood drawn are summarized in **Appendix B**.

Clinical Monitoring and Evaluation

Note: Vaccinations will be given on a 0, 2, and 6 month schedule in the US, and a 0, 2, and 4 month schedule in Mali, to allow for the third vaccination to occur just prior to the malaria transmission season in Mali.

<u>Study Day 0 (Day of First Vaccination)</u>

1. Verify that Informed Consent was obtained.
2. Verify that all applicable eligibility criteria have been met.
3. Ensure that CBC, ALT, and creatinine measurements from screening tests are within protocol-defined limits (**Section 4.5** Exclusion Criteria) before vaccinating.
4. Perform abbreviated history and physical examination, focusing on any acute complaints.
5. Mali only: during the physical examination, study staff will educate the subject regarding signs and symptoms of potential adverse events (AEs), and indications for use of antipyretics (acetaminophen/paracetamol, or ibuprofen) if fever, headache, or malaise occurs.
6. Obtain approximately 55 mL of blood for CBC with differential and platelet count, ALT, creatinine, anti-AMA1 and anti-MSP1 antibody ELISA, parasite growth inhibition assay (GIA), and B and T cell studies. An additional 5 mL of blood will also be obtained and serum will be stored. If subsequent to second vaccination the subject develops clinical signs of autoimmune or other disease, this sample will be used for baseline laboratory tests as indicated. The total volume of blood to be drawn is 60 mL.
7. Obtain blood for malaria smear, filter paper collection and urine for chloroquine testing (Mali only).
8. Obtain a urine sample for dipstick testing and ensure that protein and blood are no more than trace positive before vaccinating. Should a female subject have more than trace blood in her urine on Day 0, she will not be excluded from vaccination if she is currently menstruating.
9. For females, obtain a urine or serum sample for β-hCG testing. Ensure that the test is negative before vaccinating; a positive test will exclude the subject from the trial.
10. Record vital signs (blood pressure, temperature, heart rate, and respiratory rate).
11. Administer the vaccine.
12. Observe for at least 30 minutes after vaccination to evaluate for immediate adverse reactions.
13. US only: education by study staff during 30-minute post-immunization wait period describing proper use of digital thermometer, injection-site reaction measurement, and malaria vaccine side-effect memory enhancement tool. Study staff will also discuss signs and symptoms of potential AEs, and indications for use of antipyretics (acetaminophen/paracetamol, or ibuprofen) if fever, headache, or malaise occurs.

Study Day 1 (Mali only)

1. Perform basic history and focused physical examination (including injection site), emphasizing examination of any acute complaints.
2. Record vital signs.

Study Day 2 (Mali only)

1. Perform basic history and focused physical examination (including injection site), emphasizing examination of any acute complaints.
2. Record vital signs.

Study Day 3 ±1 Day

1. Perform basic history and focused physical examination (including injection site), emphasizing examination of any acute complaints.
2. Review memory enhancement card.(US only)
3. Record vital signs.
4. Obtain approximately 10 mL of blood for CBC with differential and platelet count, ALT, and creatinine.
5. Obtain urine for dipstick test (Mali only)

Study Day 7 ±1 Day

1. Perform basic history and focused physical examination (including injection site), emphasizing examination of any acute complaints.
2. Record vital signs.
3. Obtain approximately 10 mL of blood for CBC with differential and platelet count and serum creatinine (Mali only).
4. Obtain urine for dipstick test.
5. US only: Review Days 0-6 memory enhancement tool.

Study Day 14 ±2 Days

1. Perform basic history and focused physical examination (including injection site), emphasizing examination of any acute complaints.
2. Record vital signs.
3. Obtain urine for dipstick testing. Obtain urine or serum for β-hCG testing.
4. Obtain approximately 15 mL of blood for CBC with differential and platelet count, ALT, creatinine, anti-AMA1 and anti-$MSP1_{42}$ ELISA.

Study Day 28 ±3 Days

1. Perform directed history and assessment of any physical complaint.
2. Record vital signs.
3. Obtain urine for dipstick test.

Study Day 56 +7 (Day of Second Vaccination)

1. Perform basic history and focused physical examination, emphasizing examination of any acute complaints.
2. Mali only: during the physical examination, study staff will educate the subject regarding signs and symptoms of potential AEs, and indications for use of antipyretics

(acetaminophen/paracetamol, or ibuprofen) if fever, headache, or malaise occurs.

3. Obtain approximately 40 (35 in Mali)mL of blood for CBC with differential and platelet count, ALT, creatinine, anti-AMA1 and anti-$MSP1_{42}$ antibody ELISA, and B cell studies.
4. Obtain blood for malaria smear and filter paper collection (Mali only).
5. For females, obtain a urine or serum sample for β-hCG testing. Ensure that the test is negative before vaccinating; a positive test will exclude the subject from the trial.
6. Obtain a urine sample for dipstick testing and ensure that protein and blood are no more than trace positive before vaccinating. Should a female subject be menstruating on Day 56, she will not be excluded from vaccination if she has more than trace blood in her urine.
7. Obtain urine for chloroquine testing (Mali only).
8. Record vital signs (blood pressure, temperature, heart rate, and respiratory rate).
9. Administer the vaccine.
10. Observe for at least 30 minutes after vaccination to evaluate for immediate adverse reactions.
11. US only: Review of proper use of digital thermometer, injection-site reaction measurement tool, and memory enhancement tool (diary) by study staff during 30-minute post-immunization wait period. Study staff will also discuss signs and symptoms of potential AEs, and indications for use of antipyretics (acetaminophen/paracetamol, or ibuprofen) if fever, headache, or malaise occurs.

Study Day 57 (Mali only) (1 Day after Second Vaccination)

1. Perform basic history and focused physical examination (including injection site), emphasizing examination of any acute complaints.
2. Record vital signs.

Study Day 58 (Mali only) (2 Days after Second Vaccination)

1. Perform basic history and focused physical examination (including injection site), emphasizing examination of any acute complaints.
2. Record vital signs.

Study Day 59 ±1 Day (3 Days after Second Vaccination)

1. Perform basic history and focused physical examination (including injection site), emphasizing examination of any acute complaints.
2. Review memory enhancement tool. (US only)
3. Record vital signs.
4. Obtain approximately 10 mL of blood for CBC with differential and platelet count, ALT, and creatinine.
5. Obtain urine for dipstick test (Mali only)

Study Day 63 ±1 Day (7 Days after Second Vaccination)

1. Perform basic history and focused physical examination (including injection site), emphasizing examination of any acute complaints.
2. Record vital signs.
3. Review Days 28-34 memory enhancement tool.
4. Obtain urine for dipstick tests.
5. Obtain approximately 50 mL of blood for CBC with differential and platelet count, serum creatinine (Mali only), and B and T cell studies.

Study Day 70±2 Days (14 Days after Second Vaccination)

1. Perform basic history and focused physical examination (including injection site), emphasizing examination of any acute complaints.
2. Record vital signs.
3. Obtain urine for dipstick testing. Obtain urine or serum for β-hCG testing.
4. Obtain approximately 40 mL of blood for CBC with differential and platelet count, ALT, creatinine, anti-dsDNA, anti-AMA1 and anti-$MSP1_{42}$ antibody ELISA, T cell studies, and GIA.
5. Obtain blood for malaria smear and filter paper collection (Mali only).

Study Day 84 ±3 Days (28 Days after Second Vaccination)

1. Perform basic history and assessment of any physical complaint.
2. Record vital signs.
3. Obtain urine for dipstick tests.

Study Day 180 ±14 Days (Day of Third Vaccination) (US)

1. Perform basic history and focused physical examination, emphasizing examination of any acute complaints.
2. Obtain approximately 40 mL of blood for CBC with differential and platelet count, ALT, creatinine, anti-AMA1 and anti-$MSP1_{42}$ antibody ELISA, and B cell studies.
3. For females, obtain a urine or serum sample for β-hCG testing. Ensure that the test is negative before vaccinating; a positive test will exclude the subject from the trial.
4. Obtain a urine sample for dipstick testing and ensure that protein and blood are no more than trace positive before vaccinating. Should a female subject be menstruating on the day of vaccination, she will not be excluded from vaccination if she has more than trace blood in her urine.
5. Record vital signs (blood pressure, temperature, heart rate, and respiratory rate).
6. Administer the vaccine.
7. Observe for at least 30 minutes after vaccination to evaluate for immediate adverse reactions.

Review of proper use of digital thermometer, injection-site reaction measurement tool, and memory enhancement tool (diary) by study staff during 30-minute post-immunization wait period. Study staff will also discuss signs and symptoms of potential AEs, and indications for use of antipyretics (acetaminophen/paracetamol, or ibuprofen) if fever, headache, or malaise occurs.

Study Day 183 ±1 Day (3 Days after Third Vaccination) (US)

1. Perform basic history and focused physical examination (including injection site), emphasizing examination of any acute complaints.
2. Review memory enhancement tool.
3. Record vital signs.
4. Obtain approximately 10 mL of blood for CBC with differential and platelet count, ALT, and creatinine.

Study Day 187 ±1 Day (7 Days after Third Vaccination) (US)
1. Perform basic history and focused physical examination (including injection site), emphasizing examination of any acute complaints.
2. Record vital signs.
3. Review Days 180-187 memory enhancement tool.
4. Obtain urine for dipstick tests.
5. Obtain approximately 40 mL of blood for CBC with differential and platelet count, and B and T cell studies.

Study Day 194 ±2 Days (14 Days after Third Vaccination) (US)
1. Perform basic history and focused physical examination (including injection site), emphasizing examination of any acute complaints.
2. Record vital signs.
3. Obtain urine for dipstick testing. Obtain urine or serum for β-hCG testing.
4. Obtain approximately 40 mL of blood for CBC with differential and platelet count, ALT, creatinine, anti-dsDNA, anti-AMA1 and anti-$MSP1_{42}$ antibody ELISA, B and T cells, and GIA.

Study Day 210 ±3 Days (US)
1. Perform directed history and assessment of any physical compla$_{in}$t.
2. Record vital signs.
3. Obtain urine for dipstick testing.

Study Day 270 ±14 Days (US)
1. Perform directed history and assessment of any physical complaint.
2. Record vital signs.
3. Obtain urine for dipstick testing.
4. Obtain approximately 5 mL of blood for anti-AMA1 and anti-$MSP1_{42}$ antibody ELISA.

Study Day 360 ±14 Days (US)
1. Perform directed history and assessment of any physical complaint.
2. Record vital signs.
3. Obtain urine for dipstick tests.
4. Obtain approximately 50 mL of blood for anti-dsDNA, anti-AMA1 and anti-$MSP1_{42}$ ELISA, GIA, and T and B cell studies.

Study Day 720 ± 14 Days (US)
For US volunteers, if the volunteer consents, a phone call will be made to ask whether the volunteer has developed any autoimmune disease since his or her participation in the study. The volunteer will also be asked if he or she has been hospitalized or had any other serious illnesses. If so, additional details will be obtained regarding this illness, including medical records if possible. Volunteers will also be reminded to notify the investigators if they develop an autoimmune disease in the future and will be given contact information for such notification.
In the US only SAEs or events possibly relating to autoimmune disease will be captured during the extended follow up period.

Study Day 120 ±14 Days (Day of Third Vaccination) (Mali)

1. Perform basic history and focused physical examination, emphasizing examination of any acute complaints.
2. During the physical examination, study staff will educate the subject regarding signs and symptoms of potential AEs, and indications for use of antipyretics (acetaminophen/paracetamol, or ibuprofen) if fever, headache, or malaise occurs.
3. Obtain approximately 35 mL of blood for CBC with differential and platelet count, ALT, creatinine, anti-AMA1 and anti-$MSP1_{42}$ antibody ELISA, and B cell studies.
4. Obtain blood for malaria smear and filter paper collection
5. For females, obtain a urine or serum sample for β-hCG testing. Ensure that the test is negative before vaccinating; a positive test will exclude the subject from the trial.
6. Obtain a urine sample for dipstick testing and ensure that protein and blood are no more than trace positive before vaccinating. Should a female subject be menstruating on the day of vaccination, she will not be excluded from vaccination if she has more than trace blood in her urine.
7. Obtain urine for chloroquine testing.
8. Record vital signs (blood pressure, temperature, heart rate, and respiratory rate).
9. Administer the vaccine.
10. Observe for at least 30 minutes after vaccination to evaluate for immediate adverse reactions.

Study Day 121 (Mali only) (1 Day after Third Vaccination) (Mali)

1. Perform basic history and focused physical examination (including injection site), emphasizing examination of any acute complaints.
2. Record vital signs.

Study Day 122 (Mali only) (2 Days after Third Vaccination) (Mali)

1. Perform basic history and focused physical examination (including injection site), emphasizing examination of any acute complaints.
2. Record vital signs.

Study Day 123 ±1 Day (3 Days after Third Vaccination) (Mali)

1. Perform basic history and focused physical examination (including injection site), emphasizing examination of any acute complaints.
2. Record vital signs.
3. Obtain approximately 10 mL of blood for CBC with differential and platelet count, ALT, and creatinine.
4. Obtain urine for dipstick test

Study Day 127 ±1 Day (7 Days after Third Vaccination) (Mali)

1. Perform basic history and focused physical examination (including injection site), emphasizing examination of any acute complaints.
2. Record vital signs.
3. Obtain urine for dipstick tests.
4. Obtain approximately 50 mL of blood for CBC with differential and platelet count, and B and T cell studies and serum creatinine.

Study Day 134 ±2 Days (14 Days after Third Vaccination) (Mali)

1. Perform basic history and focused physical examination (including injection site), emphasizing examination of any acute complaints.
2. Record vital signs.
3. Obtain urine for dipstick testing. Obtain urine or serum for β-hCG testing.
4. Obtain approximately 40 mL of blood for CBC with differential and platelet count, ALT, creatinine, anti-dsDNA, anti-AMA1 and anti-$MSP1_{42}$ antibody ELISA, B and T cells, and GIA.
5. Obtain blood for malaria smear and filter paper collection

Study Day 150 ±3 Days (Mali)

1. Perform directed history and assessment of any physical complaint.
2. Record vital signs.
3. Obtain urine for dipstick testing.
4. Obtain blood for malaria smear and filter paper collection

Study Day 180 ±7 Days (Mali)

1. Perform directed history and assessment of any physical complaint.
2. Record vital signs.
3. Obtain blood for malaria smear and filter paper collection.

Study Day 210 ±14 Days (Mali)

1. Perform directed history and assessment of any physical complaint.
2. Record vital signs.
3. Obtain urine for dipstick testing.
4. Obtain approximately 5 mL of blood for anti-AMA1 and anti-$MSP1_{42}$ antibody ELISA.
5. Obtain blood for malaria smear and filter paper collection.

Study Day 240 ±7 Days (Mali)

1. Perform directed history and assessment of any physical complaint.
2. Record vital signs.
3. Obtain blood for malaria smear and filter paper collection

Study Day 270 ±7 Days (Mali)

1. Perform directed history and assessment of any physical complaint.
2. Record vital signs.
3. Obtain blood for malaria smear and filter paper collection.

Study Day 360 ±14 Days (Mali)

1. Perform directed history and assessment of any physical complaint.
2. Record vital signs.
3. Obtain urine for dipstick tests.
4. Obtain approximately 50 mL of blood for anti-dsDNA, anti-AMA1 and anti-$MSP1_{42}$ ELISA, GIA, and T and B cell studies.
5. Obtain blood for malaria smear and filter paper collection

Study Day 720 ± 14 Days (Mali)

An additional visit to the study clinic will be made and the volunteer will be asked about the development of any autoimmune disease or other serious illness and given contact information should an autoimmune disease develop, as above. Adverse events occurring during the extended passive follow up period will be captured as per usual in Mali.

SYMPTOM MEMORY ENHANCEMENT TOOL (US ONLY)

Subjects will be asked to keep daily symptom diaries recording oral temperature once during the day, as well as pain, tenderness, redness, swelling at the injection site and any systemic signs or symptoms for 6 days following each immunization. The size of any injection-site reaction will be measured using a standardized clear plastic measurement device and recorded in the symptom diary. Clinical site staff will review these diaries and record on the source document the daily temperature, injection site erythema, induration, pain and any other systemic sign or symptom noted by the subject. The diaries will not be collected.

PHOTOGRAPHS OF RASH OR INJECTION SITE REACTIONS

If a volunteer develops a rash or injection site reaction, photographs may be taken by the investigators. These photographs will not include the participant's face or any identifying scars, marks, or tattoos.

CONTRAINDICATIONS TO VACCINATION

The following criteria should be checked prior to each immunization and are contraindications to further immunization. The participant will be encouraged to remain in the safety evaluation for doses already received.

- Hypersensitivity reaction following administration of the study vaccine.
- Positive urine or serum β-hCG test.
- Urine dipstick more than trace positive for blood or protein, confirmed by urinalysis. Should a female participant's urine dipstick show more than trace blood, she will not be excluded from vaccination if she is currently menstruating.

INDICATIONS FOR DEFERRAL OF VACCINATION

If any one of the following adverse events occurs at the time of the scheduled vaccination, the participant may be either vaccinated at a later date within the allowable time interval specified in the protocol or withdrawn at the discretion of the Investigator:

- Oral temperature >37.5˚C at the time of vaccination will warrant deferral of immunization until fever resolves (within protocol-defined vaccination window).
- Any other condition that in the opinion of the Investigator poses a threat to the individual if immunized or that may complicate interpretation of the safety of vaccine following immunization.

Such individual(s) will be followed in the clinic until the symptoms resolve or the window for immunization expires. No further vaccination will be performed if the participant does not recover (oral temperature ≤37.5˚C and/or lack of symptoms) within the vaccination window described in **Section 6.5** of this protocol. The participant will be monitored for safety and immunogenicity. If the subject does not receive the second vaccination, some scheduled safety blood draws may not be performed at the discretion of the Principal Investigator. Blood draws scheduled to measure immune response will be obtained if possible. If the individual meets any of the above criteria for deferral on the day of first immunization, the Investigator may elect to exclude the participant from further participation in the study. Eligible alternates will be vaccinated instead.

SUBJECT WITHDRAWAL CRITERIA

A volunteer will not be considered to have completed the trial if any of the following reasons apply:

1. *Research terminated by Sponsor or Investigator* – applies to the situation where the entire study is terminated by the Sponsor or Investigator, or other regulatory authority for any reason.
2. *Withdrawal of consent* – applies to a subject who withdraws consent to participate in the study for any reason.
3. *Noncompliant with protocol*- applies to a volunteer who does not comply with protocol-specific visits or evaluations, on a consistent basis, such that adequate follow-up is not possible and the volunteer's safety would be compromised by continuing in the trial. This also applies to a volunteer who is lost to follow-up and is not reachable by telephone or other means of communication; and is not able to be located.
4. *Developed an Adverse Event* - applies to a participant who is withdrawn from study due to an adverse event, serious or otherwise.
5. *Other* – is used when previous categories do not apply and a written explanation is required.

If a subject withdraws or is withdrawn prior to completion of the study, the reason for this decision will be recorded in the case report forms. Any volunteer who has received at least 1 dose of vaccine will be encouraged to remain in the safety evaluation for the duration of the study. The subject's data will be included in the safety and immunogenicity analysis. If a subject is withdrawn because of an AE or SAE, the participant will be followed until resolution or stabilization of the event.

REPLACEMENT OF SUBJECTS

Subjects who withdraw or are terminated from the study prior to completion will not be replaced.

TREATMENTS THAT COULD POTENTIALLY INTERFERE WITH VACCINE-INDUCED IMMUNITY

Treatment with any of the following medications during the study may exclude a subject from receiving further doses of the study vaccine. However, the subject will be encouraged to remain in the study for the duration of the study for safety evaluations.

- Licensed vaccine in the 2-week period prior to and following each vaccination

- Receipt of immunoglobulins and/or any blood products up to 30 days after administration of the last dose of vaccine

- Chronic oral or intravenous administration (≥14 days) of immunosuppressive doses of steroids, i.e., prednisone >10 mg per day, immunosuppressants or other immune-modifying drugs from each day of vaccination to two weeks following each vaccination

- Any investigational drug or investigational vaccine other than the study vaccine during the study period

- Chloroquine or related compounds (amodiaquine or primaquine) during the study period

Clinical Laboratory Testing

Using standard techniques, the clinical laboratory will perform the following tests:

1. Complete blood count plus white blood cell differential and platelet count*
2. Serum creatinine
3. Alanine aminotransferase (ALT)
4. HIV assay (FDA-approved screening antibody assay with Western Blot Confirmation) (US only)
5. HBsAg ELISA (US) or rapid diagnostic test (Mali)
6. HCV assay (FDA-approved screening antibody assay and immunoblot confirmation or viral PCR confirmation (US) or rapid diagnostic test (Mali)
7. Urinalysis (in the event of an abnormal urine dipstick test at the clinical trial site)**
8. Anti-dsDNA (ELISA)
9. Urine for chloroquine determination (Mali)
10. Malaria smear (Mali)

* The following CBC parameters will be assessed for safety throughout the trial:
WBC, ANC (absolute neutrophil count) and/or absolute granulocyte count, Hemoglobin, and Platelet Count.

** Urine and serum β-hCG testing will be performed at the clinical trial site using an FDA approved product.

Immunologic Laboratory Testing

6.1.1 Antibody Assay (ELISA)

Anti-AMA1 and anti-MSP1$_{42}$ ELISAs will be performed at the Malaria Research and Training Center in Mali and/or the Laboratory of Malaria Immunology and Vaccinology (LMIV) in Rockville, MD. Antibody levels to the 2 antigens will be measured in serum by ELISA. Duplicate assays will be done for both 3D7 and FVO forms of the proteins. Briefly, microwell plates are coated with antigen solution. Plates are washed with TRIS-buffered saline (TBS)

containing Tween-20 (T-TBS) and blocked with TBS containing skim milk powder. After washing with T-TBS, diluted serum samples are added in triplicate and incubated at room temperature for 2 hours. After incubation, unbound antibodies are removed by washing the plates with T-TBS, and alkaline phosphatase-conjugated goat anti-human IgG solution is added to each well and incubated for 2 hours at room temperature. Plates are then washed with T-TBS, followed by adding phosphatase substrate solution to each well; the plates are then covered and incubated for 20 minutes at room temperature for color development. The plates are read immediately at 405 nm with a microplate reader. The optical density values are used to determine antibody levels by comparing to a standard curve generated from a known positive-control plasma included on each ELISA plate.

6.1.2 Growth Inhibition Assay (GIA)

GIAs will be done at the Laboratory for Malaria and Vector Research (LMVR) in Rockville, MD. The GIA is designed to determine whether antibodies obtained from an immunized person can inhibit the process of merozoite invasion into red cells. In this assay, synchronized blood-stage parasites from the FVO and 3D7 lines are incubated *in vitro* with purified IgG from subject sera for a period of 40 hours. During this period, merozoites emerge from the infected red blood cells, invade normal red blood cells, and initiate a new growth cycle. Growth is determined by measuring the production of parasite lactate dehydrogenase activity. The percentage reduction in the number of newly invaded red blood cells in the presence of the post vaccination IgG compared to the number of newly invaded red blood cells in the presence of the Day 0 purified IgG is calculated as the growth inhibitory activity.

6.1.3 B-Cell Assays

B-cell studies will be done at the Malaria Research and Training Center (MRTC) in Mali or the LMIV in Rockville, MD. The analysis of the generation and maintenance of antigen-specific memory B cells will be carried out to determine if these cells can be elicited and maintained by vaccination both in malaria-naïve and semi-immune adults. Peripheral blood lymphocytes will be obtained and assayed for the presence of antigen-specific memory B cells and for the total number of memory B cells using flow cytometry and ELISPOT assays.

6.1.4 T-Cell Assays

T-cell studies will be done at LMVR, in Rockville, MD, or at MRTC and LMIV. Antigen-specific T-cell responses to vaccination will be determined by ELISPOT and by measurement of cytokine levels in supernatants of peripheral blood mononuclear cells. Specimens collected for B cells and T cells will undergo initial processing and cell separation at the CIR-DC and MRTC, and will be shipped to LMIV and distributed to LIG and LMVR in liquid nitrogen shippers as per SOP.

6.1.5 Filter paper

Filter paper will be collected in Mali at specified time points and at unscheduled visits when malaria smears are performed. Filter papers will be stored at MRTC and will be analyzed to determine the genotype of infecting malaria species if clinical data suggest a difference between the vaccine and comparator groups.

7. USE, STORAGE, AND TRACKING OF SPECIMENS AND DATA

Samples and data collected under this protocol will be used to study malaria and related diseases, and possible adverse reactions to vaccination. No genetic testing will be performed. Access to research samples will be limited using either a locked room or a locked freezer. Samples and data will be stored using codes assigned by the investigators or their designees. Data will be kept in password-protected computers. Only investigators or their designees will have access to the samples and data.

Samples will be stored at the LMIV in Rockville, MD or at LMIV's designated repository, Thermo Scientific, Rockville, MD, or at the MRTC in Bamako, Mali. Samples will be tracked using a sample tracking software program, e.g., Freezerworks. Any loss or unanticipated destruction of samples (for example, due to freezer malfunction) or data (for example, misplacing a printout of data with identifiers) will be reported to the IRBs. Such a loss will be reported to the NIAID IRB as a protocol violation under the following classification: the violation compromises the scientific integrity of the data collected for the study.

8. RETENTION OF SPECIMENS FOR FUTURE USE

All specimens collected as part of this trial may, with the subject's permission, be stored for future research. Whether or not a volunteer agrees to storage of specimens will not affect his/her ability to participate in this trial. These samples may be used to learn more about malaria infection and other diseases. These samples will not be sold or used to make commercial products. Samples will be stored only with the volunteer's permission. The volunteer may withdraw permission for future use of specimens at any time. If a volunteer withdraws his or her permission for future use of specimens, those specimens will be destroyed. All samples stored will be labeled with the volunteer's study identification (ID) number, which cannot identify the study subject but is linkable to other research databases (e.g., from questionnaires, clinical assessments, logbooks, etc.) generated by the main study. The database will contain only the study volunteer's ID number. A master log linking the study volunteer ID number to the name of the volunteer will be maintained in a password protected database system with access limited to authorized research team members**.**

At the completion of the protocol (termination), samples and data will either be destroyed, or transferred to another existing protocol, "Research Use of Human Specimens", NIAID Protocol #08-I-N064 or a repository. In the future, other investigators (both at NIH and outside) may wish to study these samples and/or data. In that case, IRB approval must be sought prior to any sharing of samples. Any clinical information shared about the sample with or without patient identifiers would similarly require prior IRB approval. The research use of stored, unlinked, or unidentified samples (for example, as a standard for immunological analyses) may be exempt from the need for prospective IRB review and approval. Exemption requests will be submitted in writing to the NIH Office of Human Subjects Research, which is authorized to determine whether a research activity is exempt. The FMPOS ethics committee will approve additional research on stored samples collected under this protocol, even if such research has been determined to be exempt from NIAID IRB review.

9. ASESSMENT OF SAFETY

DOCUMENTING, RECORDING AND REPORTING OF ADVERSE EVENTS

At each contact with the subject, information regarding adverse events will be elicited by appropriate questioning and examinations and will be immediately documented on a source document, recorded on the Adverse Event Case Report Form (AE CRF) or electronic database and reported to the IND Sponsor, IRB and FDA as outlined below.

DEFINITIONS

ADVERSE EVENTS (AE)

An adverse event (AE) includes any unfavorable or unintended change in anatomical, physiological, or metabolic functions as indicated by physical signs, symptoms, and/or laboratory-detected changes occurring in any phase of the clinical study, whether associated with the study vaccine or active comparator, and whether or not considered vaccination related. This includes an exacerbation of pre-existing conditions and intercurrent illnesses. All AEs must be graded for severity and assessed for relationship to the investigational vaccine as described below.

Adverse Reaction (AR)

An adverse event that is caused by an investigational agent (drug or biologic)

Suspected Adverse Reaction (SAR)

An adverse event for which there is a reasonable possibility that the investigational agent caused the adverse event. "Reasonable possibility" means that there is evidence to suggest a causal relationship between the drug and the adverse event. A suspected adverse reaction implies a lesser degree of certainty about causality than adverse reaction which implies a high degree of certainty.

SERIOUS ADVERSE EVENTS

A serious adverse event (SAE) is an AE, whether considered related to the investigational vaccine or not, resulting in one of the following outcomes:

1. <u>Death</u> during the period of protocol-defined surveillance.
2. <u>Life threatening event</u>: defined as an event that places a subject at immediate risk of death at the time of the event and does not refer to an event that hypothetically might have caused death were it more severe.
3. <u>Hospitalization</u> or prolongation of a hospitalization during the period of protocol-defined surveillance: defined as at least an overnight stay in the hospital or emergency ward for treatment that would have been inappropriate if administered in the outpatient setting.
4. A <u>congenital anomaly or birth defect.</u>
5. A persistent or significant <u>incapacity</u> or substantial disruption of the ability to carry out normal life functions.
6. Any other <u>important medical event</u> that may not result in death, be life threatening, or require hospitalization, may be considered a serious AE when, based upon appropriate medical

judgment, the event may jeopardize the subject and may require medical or surgical intervention to prevent one of the outcomes listed above.

Unexpected Adverse Event

An AE is unexpected if it is not listed in the Investigator's Brochure or Package Insert (for marketed products) or is not listed at the specificity or severity that has been observed. It is the responsibility of the IND Sponsor to make this determination.

Suspected and Unexpected Serious Adverse Reaction (SUSAR)
A SUSAR is a Suspected Adverse Reaction that is both Serious and Unexpected.

Unanticipated Problem (UP)
An Unanticipated Problem is any event, incident, experience, or outcome that is:

1. unexpected in terms of nature, severity or frequency in relation to the research risks that are described in the IRB-approved protocol, informed consent document, Investigator's Brochure, or other study documents and the characteristics of the subject population being studied; and
2. possibly, probably, or definitely related to participation in the research; and
3. places subjects or others at a greater risk of harm (including physical, psychological, economic or social harm) than was previously known or recognized. (Per the IND Sponsor, an AE with a serious outcome will be considered increased risk.)

Unanticipated Problem That is Not An Adverse Event (UPnonAE)
A UP that does not fit the definition of an AE, but which may, in the opinion of the Investigator, involve risk to the subject, affect others in the research study or significantly impact the integrity of research data. Examples include occurrences of breaches of confidentiality, accidental destruction of study records, and unaccounted study drug.

Protocol Violation
A Protocol Violation is any change, divergence, or departure from the study design or procedures in an IRB-approved research protocol that has a major impact on the subject's rights, safety, or well-being and/or the completeness, accuracy or reliability of the study data.

Protocol Deviation
A Protocol Deviation is any change, divergence, or departure from the study design or procedures of an IRB-approved research protocol that does not have a major impact on the subject's rights, safety or well-being, and/or the completeness, accuracy and reliability of the study data.

ASSESSMENT OF ADVERSE EVENTS

9.1.1 Identification of Adverse Events

Assessment of safety will include clinical observation and monitoring of hematological, chemical, and immunologic parameters. Safety will be evaluated by monitoring of subjects for local and systemic adverse reactions during the course of the trial. Subjects will be closely monitored for 30 minutes following each immunization. Additionally, subjects will return to the

clinic on Days 1, (Mali only) 2, (Mali only) 3, 7, and 14 following each vaccination for clinical assessments, and approximately monthly thereafter until completion.

All AEs will be graded for severity and assessed for relationship to the study product. Reactions will be graded as described in **Section 9.1.2** in this protocol. A study clinician will be available 24 hours a day during the study evaluation period; a study clinician will stay in Bancoumana for the duration of the trial and will be available to the study participants at all times. Should a subject call on a study clinician to report an adverse event, it will be fully documented in the subject's study chart, and discussed with the Principal Investigator.

All local and systemic reactions will be captured on the appropriate case report form (CRF). Those assessed as serious will be further reported on the Sponsor's SAE report form. Adverse events judged to be possibly, probably, or definitely related to the study product will be followed to adequate resolution.

9.1.2 Determination of Severity

Severity of AEs will be assessed by the investigator as described in **Appendixes C**, **D**, and **E**. AEs not included in the Appendixes will be graded for severity using the followings definitions:

Grade 1 (Mild):	No effect on activities of daily living
Grade 2 (Moderate):	Partial limitation in activities of daily living (can complete ≥50% of baseline), or treatment given; no or minimal medical intervention/therapy required
Grade 3 (Severe):	Activities of daily living limited to <50% of baseline, medical evaluation/therapy required
Grade 4 (Life-threatening):	Extreme limitation in activity, significant assistance required; immediate medical intervention or therapy required to prevent death
Grade 5:	Death

9.1.3 Association with Receipt of the Study Vaccine

All AEs will be assessed for relationship to the study vaccine using the following definition:

<u>Definitely</u>:	Clear-cut temporal association, and no other possible cause.
<u>Probably</u>:	Clear-cut temporal association and a potential alternative cause is not apparent.
<u>Possibly</u>:	Less clear temporal association; other causes also possible.
<u>Unlikely</u>:	Temporal association between the AE and the vaccine or the nature of the event is such that the vaccine is <u>not</u> likely to have had any reasonable association with the observed illness/event (cause and effect relationship improbable but not impossible).
<u>Not Related</u>:	The AE is completely independent of vaccine administration; and/or evidence exists that the event is definitely related to another cause.

The degree of certainty with which an AE can be attributed to administration of the study vaccine will be determined by how well the event can be understood in terms of one or more of the following:

1. The event being temporally related with vaccination or reproduced on re-vaccination
2. A reaction of similar nature having previously been observed with this type of vaccine and/or formulation
3. The event having been reported in the literature for similar types of vaccines

All local (injection-site) reactions will be considered causally related to vaccination. [Episodes of clinical malaria will be considered as not related to vaccination.]

INVESTIGATOR REPORTING RESPONSIBILITIES TO THE SPONSOR

Serious Adverse Events

SAEs (whether or not they are also UPs) must be reported on the SAE/UP Report Form and sent to the RCHSPB Clinical Safety Office (CSO) by fax or e-mail attachment. All deaths and immediately life-threatening SAEs due to any cause that occur during the course of the study must be reported to RCHSPB Clinical Safety Office by the clinical site Investigator within 1 business day after the clinical site becomes aware of the event, and all other SAEs must be reported as soon as possible, but no later than 3 business days.

Serious adverse events will be reported to:

RCHSPB Clinical Safety Office
5705 Industry Lane
Frederick, MD 21704

Phone: 301-846-5301
Fax: 301-846-6224
E-mail: rchspsafety@mail.nih.gov

In addition, the clinical site Investigator will notify LMIV by email, fax, or telephone within 1 working day of notification of an SAE occurrence. (Contact: Dr. Ruth Ellis, ellisru@niaid.nih.gov, fax 301-480-1962, telephone 301-435-3064) SAEs which occur in Mali will be also be reported to the Medical Monitor within 1 working day of notification of occurrence. (Contact: Mamadou Dembélé, MD, email: hassiramadydembele@yahoo.fr, telephone 50 02/ +223 2022 5003 Cell: + 223 7604 93 87.)

The LMIV Investigator will provide copies of all SAE reports occurring during this trial to the Safety Contact at Coley Pharmaceutical Group, a Pfizer Company. These reports will include related as well as unrelated SAE reports. All SAE reports sent to Coley/Pfizer will be reported on MedWatch Form FDA 3500A and will include the Investigator's assessment of causality.

Unanticipated Problems

Non-serious AEs that are UPS must also be reported on the SAE/UP Report Form and sent to the CSO by fax or e-mail attachment no later than 7 calendar days of site awareness of the event. The UPs that are not AEs will not be reported to the Sponsor CSO.

Pregnancy
Pregnancy itself is not an AE. However, complications of pregnancies are AEs and may be SAEs. Pertinent obstetrical information for all pregnancies will be reported to the CSO via fax or email within 3 business days from site awareness of the pregnancy. Pregnancy outcome data (e.g., delivery outcome, spontaneous, or elective termination of the pregnancy) will be reported to the CSO within 3 business days of the site's awareness of the outcome.

In the event that a female participant should become pregnant and it is known that the participant has been exposed to CPG 7909, LMIV shall inform Coley Pharmaceutical Group, a Pfizer Company, of this pregnancy as soon as possible and in no event later than 15 calendar days after LMIV's initial receipt of the information.

For each pregnancy, LMIV will provide Coley with a completed current version of Coley's Pregnancy Notification Form and Pregnancy Notification Follow-Up Form. These forms are subject to minor changes and the current versions will be supplied by Coley as soon as LMIV informs Coley that a pregnancy has occurred. These forms will be completed by the clinical site Investigator and forwarded to LMIV. At the time a pregnancy is diagnosed, LMIV will submit a completed Pregnancy Notification Form to Coley as well as a Pregnancy Notification Follow-up Form at the time of pregnancy outcome. Reports of exposure during pregnancy will be entered into the Coley safety database and will be forwarded to Coley Pharmaceutical Group, a Pfizer Company, via the country office as noted in **Section 9.4.2.**

INVESTIGATOR REPORTING RESPONSIBILITIES TO THE NIAID IRB

Expedited Reporting to the NIAID IRB
Unanticipated problems that are either AEs or non-AEs and protocol violations will be reported within 7 calendar days of site awareness. Serious Adverse Events that are possibly, probably, or definitely related to the research will be reported to the NIAID IRB within 7 days of site awareness, regardless of expectedness. Investigators will report within 15 days on any other event or condition regardless of grade, which in their judgment represents an event reportable to the NIAID IRB.

Annual Reporting to the NIAID IRB
The following items will be reported to the NIAID IRB in summary at the time of Continuing Review:

- All unanticipated problems (including AEs and non-AEs)
- Expected serious adverse events that are possibly, probably, or definitely related to the research
- Serious adverse events that are not related to the research
- All protocol deviations which in the opinion of the Investigator should be reported
- Any trends or events which in the opinion of the Investigator should be reported

INVESTIGATOR REPORTING RESPONSIBILITIES TO LOCAL IRB(S)

Site Investigators must comply with the reporting requirements of their local IRB/Ethics Committees (i.e. WIRB and FMPOS) The Investigators are also responsible for submitting IND

Safety Reports and UP summaries that are received from the IND Sponsor to their local IRB/Ethics Committee.

SPONSOR'S REPORTING RESPONSIBILITIES

In accordance with the FDA Code of Federal Regulations (21 CFR 312.32), the IND sponsor RCHSPB must report serious and unexpected suspected adverse reactions (SUSARS) to the FDA and all participating Investigators in the form of a written IND Safety Report. AEs that are also UPs will be summarized by the IND Sponsor and distributed to the Investigators. Deaths and life-threatening events with any possible relationship to a study intervention must be reported to the FDA by telephone or fax as soon as possible but within 7 calendar days of RCHSPB's awareness. This initial report must be followed by as complete a written report as possible within 8 additional calendar days. All other IND Safety Reports must be submitted to the FDA as soon as possible, but no later than 15 calendar days after RCHSPB is notified of the SAE.

SAEs that do not meet the requirements for expedited reporting and all documented adverse events will be reported to the FDA in the IND annual report.

The FDA also requires sponsors to submit a written Safety Report of all serious and unexpected adverse events to all participating investigators of a trial. All participating investigators at all sites will be notified of any unexpected SAEs occurring at other sites in an IND Safety Report from RCHSPB. RCHSPB will also notify investigators of any revisions to the protocol or to the consent, or study closure. The study investigators in this study have the responsibility of promptly reporting all SAEs so that RCHSPB can comply with these regulations.

In addition, the Safety Office of RCHSPB will forward to Coley copies of all IND Safety Reports, using the same format as used to notify the FDA. Safety reports to Coley will not contain identifying or confidential information. All SAEs and safety related information are to be in writing and may be transmitted by fax using the Pfizer Investigator-Initiated Research Reportable Event Fax Cover Sheet to:

For U.S. Sites:

Pfizer U.S. Clinical Trial Department
235 East 42nd St. Mailstop: 685/14/01
New York, NY 10017-
Fax: (866) 997-8322
Telephone: (212) 733-8842

For Mali, West Africa:

Sendra Marlene Aklomy KOUAME
Pfizer Afrique de l'Ouest
Bureau de Cote d'Ivoire
Avenue Blohorn
Impassse de la Savonnerie 08 BP 2004
Abidjan 08 Cote d'Ivoire
Email: sendramarleneaklomy.KOUAME@pfizer.com

Telephone: +225 22 48 37 30
Fax: +225 22 48 63 03

HALTING RULES

The Principal Investigator will closely monitor study data as they become available and will make determinations regarding the presence and grading of adverse events. Adverse events will be evaluated with regard to the known complications associated with administration of CPG 7909 and other vaccine components. If a dose of vaccine is considered unacceptably reactogenic (as described in the following criteria), the study will be halted. No new enrollments and no further vaccinations will be administered by the Investigators until reviewed by the SMC and study IND Sponsor. A report of SMC recommendations will be submitted to the IRB. The following criteria will be used to define unacceptable reactogenicity of the malaria vaccine:

1. One or more subjects experience an SAE as defined in **Section 9.2** of this protocol that is determined to be possibly, probably, or definitely related to the vaccine, **or**
2. One or more subjects experience a hypersensitivity reaction that is probably or definitely related to the vaccine, **or**
3. Any severe clinical illness occurs that is not explained by a diagnosis that is unrelated to vaccination, **or**
4. Two or more subjects in any dose cohort (low dose US, high dose US, or Mali) experience any Grade 2 or higher laboratory abnormality (see **Appendix D**), or Grade 3 systemic AE that is determined to be possibly, probably, or definitely related to the vaccine as defined in this protocol. This does not include Grade 2 neutropenia resolving within 7 days of vaccination, which is an expected laboratory abnormality with CPG 7909 and which has not been associated with clinically significant events.

The IRBs, the NIAID, the pharmaceutical supporter(s), the FDA, or other government agencies, may discontinue the study at any time. Subsequent review of serious, unexpected, and related adverse events by the Medical Monitor, SMC or IRB, the IND sponsor, the FDA, and other regulatory authorities may also result in suspension of further administration of vaccine at the clinical site. The FDA, other regulatory authorities, and the study sponsor(s) retain the authority to suspend additional enrollment and administration of vaccine for the entire study as applicable.

10. CLINICAL MONITORING

SITE MONITORING PLAN

To ensure that the rights, safety, and well- being of the human subjects enrolled on this trial and to validate the Investigator's adherence to the protocol, applicable government regulations, and ICH/GCP guidelines, RCHSPB will provide oversight and monitor the compliance of this trial. Monitors will visit the clinical site to monitor all aspects of the trial in accordance with appropriate regulations. The objectives of a monitoring visit will be:

1. to verify the prompt reporting of all data points, including SAEs
2. to check the availability of signed informed consent
3. to compare individual subject's records (CRFs, electronic data, CRIMSON pulls) to the

source documents (supporting data, laboratory specimen records, clinical notes)

4. to ensure protection of study subjects, compliance with the protocol, and accuracy and completeness of records

The monitors will also inspect the clinical site's regulatory files to ensure that applicable regulatory requirements (FDA, OHRP) and guidelines (ICH) are being obeyed. During the monitoring visits, the Principal Investigator and/or designated study staff will be available to discuss the study. The site Principal Investigator will provide direct access and allow the study monitors, LMIV, and regulatory authorities in Mali and US to access all study-related documents.

Prior to the start of the study, the Principal Investigators will be informed of the frequency of the monitoring visits and will be given reasonable notification prior to each visit.

Quality control procedures will be implemented beginning with the data entry system, and data quality control checks that will be run on the database will be generated. Any missing data or data anomalies will be communicated to the site for clarification/resolution.

SAFETY MONITORING PLAN

10.1.1 Safety Monitoring Committee (SMC)

The LMIV, in consultation with the clinical trial sites and RCHSPB, will select 3 independent candidates to serve as monitors to advise RCHSPB and the study investigators on the trial. These individuals will be independent of LMIV and the clinical trial sites. RCHSPB will evaluate the candidates and coordinate the functions of the SMC. The SMC's primary responsibility will be to monitor subject safety. The Principal Investigator is responsible for ensuring that the SMC is aware of all new safety information. The SMC will meet to review the protocol prior to study initiation, and will review cumulative safety data up to at least 1 week after the first two vaccinations are complete in the US, and will give approval to proceed prior to enrollment and vaccinations in Mali. The SMC will also meet if stopping criteria are met, or as needed at the discretion of the SMC and/or study investigators. At any scheduled or unscheduled meeting, the SMC will review cumulative safety data for evidence of study-related AEs, adherence to the protocol, and factors that may affect outcome or study data such as protocol violations and losses to follow-up. One member of the SMC will be a Malian who is independent of the MRTC. Summaries of all SMC meetings will be sent to the FDA after review and approval by the SMC chair.

10.1.2 Medical Monitor

An independent medical monitor, Professor Mamadou Dembélé, has been appointed for oversight of safety in this trial in Mali. The medical monitor will be available to advise the Investigators on study-related medical questions or problems, and to act as a representative for the subjects' welfare. Additionally, the medical monitor may ask to convene a safety monitoring committee meeting for review of any safety issue or adverse event. Should the study be halted after vaccinations have begun in Mali, the medical monitor will participate in the safety data review prior to administration of further vaccinations.

11. **STATISTICAL CONSIDERATIONS**

DESCRIPTION OF STATISTICAL METHODS

This study, like other Phase 1 studies, is basically exploratory rather than confirmatory; its purpose is to estimate AE rates and patterns of immune responses rather than to test formal statistical hypotheses. Estimates will be presented with their 95% confidence intervals. Descriptive approaches will be used to meet the protocol objectives as stated in **Section 2** of this protocol, as well as formal statistical tests as outlined below. Results will be presented in tabular format, as well as graphically where appropriate.

11.1.1 Primary Objective

The primary objective of this study is to assess safety and reactogenicity of BSAM-2/Alhydrogel®+CPG 7909 in malaria-naïve US adults and semi-immune Malian adults.

a. The frequency of systemic and local AEs will be summarized.

b. A line listing of each clinical and laboratory AE classified as local, solicited, or other will be displayed in tables stratified by vaccine allocation and study site.

c. AEs will be summarized by severity and relationship to vaccine.

d. The proportion of subjects with at least 1 adverse event will be compared by vaccine and/or dose group within each study site, and tests performed to assess whether these groups differ with respect to these proportions. To see if there is a difference in adverse events between the initial vaccination and the subsequent vaccinations, a sign test will be performed, where the response for each subject is the difference between the numbers of adverse events in the 2 weeks following each vaccination.

e. SAEs occurring within the study period will be listed by relationship to vaccine.

11.1.2 Secondary Objective

The secondary objective of this study is to determine the antibody response of the combination vaccine to the AMA1 and $MSP1_{42}$ proteins as measured by antibody levels and growth inhibition.

Anti-AMA1 and anti-$MSP1_{42}$ antibody will be measured by ELISA on Study Days 0, 14, 56, 70, 180, and 194, 270, and 360. Peak antibody responses and longevity of response will be shown graphically. Antibody responses between the dose groups (US) and vaccine groups (Mali) will be compared. The primary antibody response will be the change from Day 0 to Day 42 antibody response, and Wilcoxon rank sum tests will be performed. Similar tests for changes between Day 0 and other Study Days will be performed as exploratory analyses. Immunogenicity in US adults (greater than 4-fold increase in antibody titer from baseline to peak response) is expected to be demonstrated. In Mali, interpretation of immunogenicity data is more difficult given pre-existing antibody titers and the likelihood of natural malaria infection during the study, and whether or not a significant difference in antibody response is detected between the groups will not be a determining factor for further studies. In Mali, if a difference in antibody response between the groups is detected, it will be described. However, this study is powered for safety

data alone, and whether or not a significant difference in antibody response is detected between the groups in Mali will not be a determining factor for further studies in children.

Graphs will display parasite growth inhibition expressed as a percentage of inhibition. Non-parametric methods will be used to compare inhibition as a function of treatment group and clone and to examine correlations between levels of antibody as judged by ELISA with GIA activity.

11.1.3 Exploratory Objectives

- To determine the extent to which antibody response to the AMA1 and $MSP1_{42}$ antigens is correlated when the combination vaccine (BSAM-2/Alhydrogel®+CPG 7909) is given.
- To determine T- and B-cell responses to vaccination.

T- and B-cell responses at different study days will be shown graphically for each dose group (US) or treatment group (Mali).

Should the study be terminated early (see **Section 9.5)**, the investigative team will discuss with the SMC the reason for termination and determine which study questions can be addressed in an unbiased manner with the available data. The available data will be analyzed and interpreted in light of early termination.

Sample Size and Power Calculations

The study is powered to provide sufficient safety data before proceeding step-wise (first dose ranging in the US, and then to Malian adults) towards the target population (young children in an endemic area). A sample size of 12 in each group gives a probability of 0.80 for detecting 1 or more adverse events that occur with a probability of 0.125 per subject. We have included 15 per dose group in case of withdrawals or loss to follow-up.

12. HUMAN SUBJECT PROTECTIONS AND ETHICAL OBLIGATIONS

This research will be conducted in compliance with the protocol, Good Clinical Practices (GCP), and all applicable regulatory requirements.

Institutional Review Board

A copy of the protocol, informed consent forms, other information to be completed by participants, such as questionnaires, and any proposed advertising/recruitment materials or letters to the participants will be submitted to the NIAID, FMPOS, and Western IRBs for written approval. The investigator must submit and obtain approval from the named IRBs for all subsequent amendments to the protocol, informed consent documents, and other study documentation referenced above. The investigator will be responsible for obtaining IRB approval of the annual Continuing Review throughout the duration of the study. The investigator will notify the IRB of violations of the protocol and serious adverse events.

Informed Consent Process

Site-specific procedures for informed consent are described in **Sections 4.2** and **4.3**. Informed consent is a process that is initiated prior to the individual's agreeing to participate in the study and continuing throughout the individual's study participation. Extensive discussion of risks and

possible benefits of this therapy will be provided to the participants and their families. Consent forms describing in detail the study agent/intervention(s), study procedures, and risks will be given to the participant, and written documentation of informed consent is required prior to starting study agent/intervention. Consent forms will be approved by all participating IRBs, and the participant will be asked to read and review the document, or will have the document read to him or her in case of illiteracy. Upon reviewing the document, the investigator will explain the research study to the participant and answer any questions that may arise. The participants will sign or fingerprint the informed consent document prior to any procedures being done specifically for the study. The participants will have sufficient opportunity to discuss the study and process the information in the consent process prior to agreeing to participate. The participants may withdraw consent at any time throughout the course of the trial.

The acquisition of informed consent will be documented in the participant's research chart, as required by 21 CFR 312.62. The informed consent form will be signed and personally dated by the participant and the person who conducted the informed consent discussion, and by an additional witness in Mali. The original signed informed consent form will be retained in the participant's chart and a signed and dated copy will be provided to the participant.

JUSTIFICATION FOR EXCLUSION OF CHILDREN

This study will not enroll children. The vaccine candidate being tested in this protocol has not yet been tested in children or malaria-exposed people. It is felt that insufficient data are available to judge the potential risk to children living in malaria-endemic areas. Once immunogenicity and safety are established in US adults and safety is established in malaria-exposed adults in Mali, we plan to vaccinate children in Mali.

PARTICIPANT CONFIDENTIALITY

The investigator will ensure that the subject's anonymity is maintained. Subjects will not be identified in any publicly released reports of this study. All records will be kept confidential to the extent provided by federal, state, and local law. The study monitors and other authorized representatives of the Sponsor may inspect all documents and records required to be maintained by the Investigator, including but not limited to, medical records. The investigator will inform the subjects that the above-named representatives will review their study-related records without violating the confidentiality of the subjects. All laboratory specimens, evaluation forms, reports, and other records that leave the site will be identified only by a coded number in order to maintain subject confidentiality. All records will be kept locked and all computer entry and networking programs will be done with coded numbers only. Clinical information will not be released without written permission of the subject, except as necessary for monitoring by IRB, the FDA, the NIAID, the OHRP, Coley Pharmaceutical Group, a Pfizer Company,, or the sponsor's designee.

RISKS

Risks to the participants are associated with venipuncture and with immunization. These risks are outlined below:

12.1.1 Venipuncture

Risks occasionally associated with venipuncture include pain, bruising, bleeding and infection at

the site of venipuncture, lightheadedness, and rarely, syncope.

12.1.2 Immunization

Possible local vaccine reactions include pain, swelling, erythema, induration, limitation of limb movement for several days, lymphadenopathy, or pruritus at the injection site. Local subcutaneous (SQ) nodules, believed to be granulomatous reactions to aluminum, have been observed with use of aluminum-based adjuvants. Thus, most aluminum-absorbed vaccines are injected intramuscularly (IM) rather than SQ. Systemic reactions such as fever, chills, headache, fatigue, malaise, myalgia, and joint pain may also possibly occur, with some reactions moderate or severe. Immediate hypersensitivity reactions including urticaria, anaphylaxis, or other IgE-mediated responses are possible as with any vaccine. As with any investigational vaccine, there is a theoretical possibility of risks about which we have no present knowledge. Subjects will be informed of any such risks should further data become available.

CPG 7909 is a potent immune activator, and has the theoretical potential to overcome the normal tolerance of the immune system for self antigens and thereby induce autoimmune disease. In a number of murine models, administration of CPG 7909 has been associated with development of certain autoimmune phenomena. However, the relevance of this finding in humans is unclear because, while TLR-9 receptors are widely distributed among immune cells in mice, they appear to be restricted to B cells and plasmacytoid dendritic cells in humans [29]. While subjects in several studies using CPG 7909 have developed detectable antibody to dsDNA, no subject in any study to date has developed signs or symptoms of a lupus-like autoimmune disease. Anti-dsDNA antibodies have generally occurred after multiple vaccinations, but declined to baseline after termination of vaccination. The clinical significance of the antibody elevations is unknown. One patient in a study investigating immune therapy for treatment of melanoma exhibited elevated antimicrosomal antibodies while receiving CPG 7909 along with peptides and Montanide. The patient, who had a known history of sub-clinical enlargement of the thyroid gland with borderline insufficiency of thyroid function associated with elevated TSH and no clinical symptoms, had a long-term exposure to anticancer immune therapy prior to CPG 7909 treatment. Pre-CPG 7909 blood levels of antimicrosomal antibodies were not assayed. No other patients who have received CPG 7909 are known to have developed autoimmune disease. One patient enrolled in a clinical trial using a related CpG (1018-ISS) as an adjuvant for a hepatitis B vaccine (Heplisav™) developed Wegener's Granulomatosis, an autoimmune vasculitis, after vaccination. Subjects in this study will be closely monitored for laboratory evidence of autoimmunity by routinely examining urine for protein and blood (as a marker of autoimmune nephritis). Serum will be collected for anti-dsDNA testing. Clinical signs or symptoms of autoimmunity will also be monitored closely.

Other clinical studies of CPG 7909 used as an adjuvant have shown a moderately increased incidence of mild to moderate local and systemic AEs after vaccination (injection site pain, myalgia, fatigue). Transient lymphopenia, neutropenia, and thrombocytopenia have been seen among patients treated with CPG 7909. These AEs will be monitored and recorded as is standard practice. A Phase 3 clinical study of CPG 7909 in patients with advanced nonsquamous cell lung cancer showed a possible increase in sepsis and thrombocytopenia with bleeding events in subjects receiving CPG 7909. No events such as this have been seen in the studies using CPG

7909 as a vaccine adjuvant described in **Section 1.6**. Subjects with fever are excluded from vaccination and hematologic parameters including platelet counts will be monitored.

With the exception of the Phase 1 study of AMA1-C1/Alhydrogel®+CPG 7909 in Malian adults described in **Section 1.6.3**, CPG 7909 has not been administered to humans in malaria-endemic areas, or in populations with a high burden of infectious disease. It is possible that the safety profile and immunologic response to CPG 7909 may be different in these populations.

Animal studies have found serious, unexpected fetal and maternal problems when CPG 7909 was given to pregnant rats and rabbits. These studies used a dose of CPG 7909 more than 100 times higher than the dose used in this study. These problems included decreased maternal and fetal weight gain as well as a variety of malformations of the fetal skeleton and organs. For this reason, women who are pregnant or who may become pregnant will not be enrolled in the study. Women who participate in the study must use a reliable method of birth control before the start of the study and for at least 3 months after receiving the final vaccination. Pregnancy testing will be completed on female subjects multiple times during the trial. Any female participant interested in contraceptive methods will be referred to the local health center family planning services for evaluation and institution of an appropriate contraceptive method.

BENEFITS

Participants will not receive any direct benefit from participation in this study. It is hoped that information gained in this study will contribute to the development of a safe and effective malaria vaccine.

Mali: Free medical treatment will be provided to all enrolled participants during the active immunization phase and the follow-up period. The pharmacy at the clinic will have sufficient provisions to provide participants with drugs for the treatment of minor illnesses free of charge. If further evaluation or treatment is necessary, the participant will be referred to 1 of the National Hospitals in Bamako. If the investigators judge that a participant requires hospitalization in Bamako, referral and transportation will be arranged and the medical management of the participant will be monitored by a senior physician-investigator and the local Medical Monitor. Medical care for ailments not related to vaccination will not extend beyond the study period. Medical care for ailments related to vaccination will extend at least until the condition has resolved. Participants in Mali will also receive vaccination against Euvax B/Hepatitis B Vaccine, either during the course of the study or after study completion (if they choose to be vaccinated).

Study participants in Mali who develop clinical malaria during the course of the study will be treated according to the guidelines of the Malian National Malaria Control Program.

COMPENSATION

12.1.3 US

Participants will be paid $75 for their screening visit and $75 per visit during participation in the study. On vaccination days the volunteers will be compensated $100 because they are required to stay in the clinic for a substantially longer period of time. Participants will be paid for the screening visit and for each clinic visit if enrolled. Volunteers will only be compensated for

screening if they are enrolled in the trial. Volunteers who attend as alternates on the day of first vaccination but are not enrolled, will receive payments for the screening visit and 1 clinic visit. A bonus of $200 will be paid for completion of all visits. The total payment (up to $1625) will be divided over the course of the study with the bonus dispensed upon completion of the trial.

12.1.4 Mali

Volunteers will not be given any monetary compensation. Volunteers will be given rice and millet, in 3 equal installments, to compensate for the time taken to come to the study clinic for study-related visits. Compensation with cereal rather than money has been decided in consultation with village elders and the amount of cereal has been determined after consultation with the Mali IRB, which recommends about USD $6 for each scheduled visit with blood drawn and USD $3 for each scheduled visit without blood drawn (total value of approximately USD$129), since visits with blood drawn require more time than visits without blood drawn. The first installment of cereal will be distributed after all participants have received the first injection, and the second will be distributed after the second vaccination, and the remainder at the conclusion of the study.

Throughout Mali, the availability of food is subject to seasonal variation in relation to the harvest season. However, there is no recent history of famine or starvation. In the region of our study site, while cases of pediatric malnutrition are occasionally seen at the village health clinic, these are attributable to poor feeding habits rather than to scarcity of food, and the intervention is to educate parents to provide more nutritional foods to small children. The total amount of food will last an average family approximately 4 weeks. The type of food distributed – rice and millet – are staple starches that are typically served accompanied by a sauce containing some sort of meat as well as vegetables, and therefore are only a part of the local diet. This amount of compensation is consistent with what we have provided to participants of longitudinal studies in Mali for several years, and has been carefully considered by the local Malian Ethics Committee, which has determined that it is appropriate compensation for time lost to study procedures, and is not coercive.

13. **DATA HANDLING AND RECORD KEEPING**

SOURCE DOCUMENTATION

Paper CRFs will be used at the Mali site and CRIMSON will be used at the US site. Study data will be collected on case report forms (CRFs) designed for the study and study-specific databases.

Complete source documentation (laboratory test reports, hospital or medical records, progress notes, observations, subject diaries, etc.) is required for every study subject for the duration of the study. The subject’s medical record must record his/her participation in the clinical trial and, after unblinding, the randomization treatment received (with doses and frequency) or other concomitant medications or interventions administered, as well as any adverse reactions experienced during the trial. Data from source documentation and volunteer symptom diaries for subjects enrolled in the study will be entered into the CRIMSON Data System. The data entry is to be completed on an ongoing basis during the study. Data entered into CRIMSON shall be performed by authorized individuals. Corrections to the data system shall be tracked electronically (password protected) with time, date, individual making the correction, and what

was changed. Source documentation should support the data collected in CRIMSON**,** and must be signed and dated by the person recording and/or reviewing the data.

The Investigator is responsible for the accuracy, completeness and timeliness of the data reported to the Sponsor in the CRIMSON Data System. All data entered into CRIMSON should be reviewed by the Investigator and signed as required with written or electronic signature, as appropriate. Data reported in CRIMSON should be consistent with source documents or the discrepancies should be explained. Source documentation will be made available for review or audit by the Sponsor or designee and any applicable Federal authorities.

Retention of Study Records

The investigator is responsible for retaining all essential documents listed in the ICH Good Clinical Practice Guideline. All essential documentation for all participants are to be maintained by the investigators in a secure storage facility for a minimum of three years, per DHHS (45 CFR 46.115(b)). The FDA requires study records to be retained for up to two years after marketing approval or disapproval (21 CFR 312.62), or until at least 2 years have elapsed since the formal discontinuation of clinical development of the investigational agent for a specific indication. These records are also to be maintained in compliance with IRB/EC, state, and federal medical records retention requirements, whichever is longest. All stored records are to be kept confidential to the extent provided by federal, state, and local law. It is the investigator's responsibility to retain copies of source documents until receipt of written notification to the contrary from the Regulatory Compliance and Human Subjects Protection Branch (RCHSPB) of the National Institute of Allergy and Infectious Diseases (NIAID). No study document should be destroyed without prior written agreement between RCHSPB/NIAID and the Principal Investigator. Should the investigator wish to assign the study records to another party and/or move them to another location, the investigator must provide written notification of such intent to RCHSPB/NIAID with the name of the person who will accept responsibility for the transferred records and/or their new location. NIAID must be notified in writing and written NIAID permission must be received by the site prior to destruction or relocation of research records.

Protocol Revisions

No revisions to this protocol will be permitted without documented approval from the IRBs that granted the original approval for the study. Any change to the protocol will be submitted to the sponsor and to the participating IRBs as a protocol amendment; and changes not affecting risk to subjects may request an expedited review. In the event of a medical emergency, the Investigator shall perform any medical procedures that are deemed medically appropriate and will notify the IND Sponsor of all such occurrences.

Publication Policy

Following completion of the study, the investigators may publish the results of this research in a scientific journal. The International Committee of Medical Journal Editors (ICMJE) member journals have adopted a trials-registration policy as a condition for publication. This policy requires that all clinical trials be registered in a public trials registry. At the time of its approval by the NIAID IRB, this trial will be registered in ClinicalTrials.gov, which is sponsored by the National Library of Medicine.

14. **REFERENCES**

References

[1] WHO. www.who.int/tdr/diseases/malaria/diseaseinfo.html . 2004.
Ref Type: Internet Communication

[2] Sachs J, Malaney P. The economic and social burden of malaria. Nature 2002 Feb 7;415(6872):680-5.

[3] WHO. The African Malaria Report. http://www.rbm.who.int/amd2003/amr2003/amr_toc.htm . 2003.
Ref Type: Internet Communication

[4] Richie TL, Saul A. Progress and challenges for malaria vaccines. Nature 2002 Feb 7;415(6872):694-701.

[5] Healer J, Crawford S, Ralph S, McFadden G, Cowman AF. Independent translocation of two micronemal proteins in developing Plasmodium falciparum merozoites. Infect Immun 2002 Oct;70(10):5751-8.

[6] Narum DL, Thomas AW. Differential localization of full-length and processed forms of PF83/AMA-1 an apical membrane antigen of Plasmodium falciparum merozoites. Mol Biochem Parasitol 1994 Sep;67(1):59-68.

[7] Triglia T, Healer J, Caruana SR, et al. Apical membrane antigen 1 plays a central role in erythrocyte invasion by Plasmodium species. Mol Microbiol 2000 Nov;38(4):706-18.

[8] Florens L, Washburn MP, Raine JD, et al. A proteomic view of the Plasmodium falciparum life cycle. Nature 2002 Oct 3;419(6906):520-6.

[9] Silvie O, Franetich JF, Charrin S, et al. A role for apical membrane antigen 1 during invasion of hepatocytes by Plasmodium falciparum sporozoites. J Biol Chem 2004;279(10):9490-6.

[10] Kennedy MC, Wang J, Zhang Y, et al. In vitro studies with recombinant *Plasmodium falciparum* apical membrane antigen 1 (AMA1): production and activity of an AMA1 vaccine and generation of a multiallelic response. Infect Immun 2002 Dec;70(12):6948-60.

[11] Malkin EM, Diemert DJ, McArthur JH, et al. Phase 1 clinical trial of apical membrane antigen 1: an asexual blood-stage vaccine for Plasmodium falciparum malaria. Infect Immun 2005 Jun;73(6):3677-85.

[12] Giersing B, Miura K, Shimp R, et al. Posttranslational Modification of Recombinant Plasmodium falciparum Apical Membrane Antigen 1: Impact on Functional Immune Responses to a Malaria Vaccine Candidate. Infect Immun 2005 Jul;73(7):3963-70.

[13] Deans JA, Knight AM, Jean WC, Waters AP, Cohen S, Mitchell GH. Vaccination trials in rhesus monkeys with a minor, invariant, Plasmodium knowlesi 66 kD merozoite antigen. Parasite Immunol 1988 Sep;10(5):535-52.

[14] Collins WE, Pye D, Crewther PE, et al. Protective immunity induced in squirrel monkeys with recombinant apical membrane antigen-1 of Plasmodium fragile. Am J Trop Med Hyg 1994 Dec;51(6):711-9.

[15] Stowers AW, Kennedy MC, Keegan BP, Saul A, Long CA, Miller LH. Vaccination of monkeys with recombinant *Plasmodium falciparum* apical membrane antigen 1 confers protection against blood-stage malaria. Infect Immun 2002 Dec;70(12):6961-7.

[16] Thomas AW, Trape JF, Rogier C, Goncalves A, Rosario VE, Narum DL. High prevalence of natural antibodies against Plasmodium falciparum 83-kilodalton apical membrane antigen (PF83/AMA-1) as detected by capture-enzyme-linked immunosorbent assay using full-length baculovirus recombinant PF83/AMA-1. Am J Trop Med Hyg 1994 Dec;51(6):730-40.

[17] Lal AA, Hughes MA, Oliveira DA, et al. Identification of T-cell determinants in natural immune responses to the Plasmodium falciparum apical membrane antigen (AMA-1) in an adult population exposed to malaria. Infect Immun 1996 Mar;64(3):1054-9.

[18] Udhayakumar V, Kariuki S, Kolczack M, et al. Longitudinal study of natural immune responses to the Plasmodium falciparum apical membrane antigen (AMA-1) in a holoendemic region of malaria in western Kenya: Asembo Bay Cohort Project VIII. Am J Trop Med Hyg 2001 Aug;65(2):100-7.

[19] Marshall VM, Zhang L, Anders RF, Coppel RL. Diversity of the vaccine candidate AMA-1 of Plasmodium falciparum. Mol Biochem Parasitol 1996 Apr;77(1):109-13.

[20] Polley SD, Conway DJ. Strong Diversifying Selection on Domains of the Plasmodium falciparum Apical Membrane Antigen 1 Gene. Genetics 2001 Aug;158(4):1505-12.

[21] Escalante AA, Grebert HM, Chaiyaroj SC, et al. Polymorphism in the gene encoding the apical membrane antigen-1 (AMA-1) of Plasmodium falciparum. X. Asembo Bay Cohort Project. Mol Biochem Parasitol 2001 Apr;113(2):279-87.

[22] McBride JS, Heidrich HG. Fragments of the polymorphic Mr 185,000 glycoprotein from the surface of isolated Plasmodium falciparum merozoites form an antigenic complex. Mol Biochem Parasitol 1987 Feb;23(1):71-84.

[23] Gerold P, Schofield L, Blackman MJ, Holder AA, Schwarz RT. Structural analysis of the glycosyl-phosphatidylinositol membrane anchor of the merozoite surface proteins-1 and -2 of Plasmodium falciparum. Mol Biochem Parasitol 1996 Jan;75(2):131-43.

[24] Haldar K, Ferguson MA, Cross GA. Acylation of a Plasmodium falciparum merozoite surface antigen via sn-1,2-diacyl glycerol. J Biol Chem 1985 Apr 25;260(8):4969-74.

[25] Blackman MJ, Whittle H, Holder AA. Processing of the Plasmodium falciparum major merozoite surface protein-1: identification of a 33-kilodalton secondary processing product which is shed prior to erythrocyte invasion. Mol Biochem Parasitol 1991 Nov;49(1):35-44.

[26] Blackman MJ, Scott FT, Shai S, Holder AA. Antibodies inhibit the protease-mediated processing of a malaria merozoite surface protein. J Exp Med 1994 Jul 1;180(1):389-93.

[27] Udhayakumar V, Anyona D, Kariuki S, et al. Identification of T and B cell epitopes recognized by humans in the C-terminal 42-kDa domain of the Plasmodium falciparum merozoite surface protein (MSP)-1. J Immunol 1995 Jun 1;154(11):6022-30.

[28] Egan A, Waterfall M, Pinder M, Holder A, Riley E. Characterization of human T- and B-cell epitopes in the C terminus of Plasmodium falciparum merozoite surface protein 1: evidence for poor T-cell recognition of polypeptides with numerous disulfide bonds. Infect Immun 1997 Aug;65(8):3024-31.

[29] Egan AF, Morris J, Barnish G, et al. Clinical immunity to Plasmodium falciparum malaria is associated with serum antibodies to the 19-kDa C-terminal fragment of the merozoite surface antigen, PfMSP-1. J Infect Dis 1996 Mar;173(3):765-9.

[30] Egan AF, Burghaus P, Druilhe P, Holder AA, Riley EM. Human antibodies to the 19kDa C-terminal fragment of Plasmodium falciparum merozoite surface protein 1 inhibit parasite growth in vitro. Parasite Immunol 1999 Mar;21(3):133-9.

[31] O'Donnell RA, Koning-Ward TF, Burt RA, et al. Antibodies against merozoite surface protein (msp)-1(19) are a major component of the invasion-inhibitory response in individuals immune to malaria. J Exp Med 2001 Jun 18;193(12):1403-12.

[32] Singh S, Kennedy MC, Long CA, Saul AJ, Miller LH, Stowers AW. Biochemical and immunological characterization of bacterially expressed and refolded Plasmodium falciparum 42-kilodalton C-terminal merozoite surface protein 1. Infect Immun 2003 Dec;71(12):6766-74.

[33] Qari SH, Shi YP, Goldman IF, Nahlen BL, Tibayrenc M, Lal AA. Predicted and observed alleles of Plasmodium falciparum merozoite surface protein-1 (MSP-1), a potential malaria vaccine antigen. Mol Biochem Parasitol 1998 May 1;92(2):241-52.

[34] Sakihama N, Kimura M, Hirayama K, et al. Allelic recombination and linkage disequilibrium within Msp-1 of Plasmodium falciparum, the malignant human malaria parasite. Gene 1999 Apr 1;230(1):47-54.

[35] Dicko A, Diemert D, Sagara I, et al. Impact of a *Plasmodium falciparum* AMA1 Vaccine on Antibody Responses in Adult Malians. *PLoS One*. 2007 Oct17;2(10):e1045

[36] Dicko A, Sagara I, Ellis RD, et al. Phase 1 study of a combination AMA1 blood stage malaria vaccine in Malian children. *PLoS One*. 2008 3(2):e15673

[37] Mullen GE, Ellis RD, Miura K, et al. Phase 1 Trial of AMA1-C1/Alhydrogel plus CPG 7909: An Asexual Blood-Stage Vaccine for Plasmodium falciparum Malaria. PLoS One. 2008 3(8):e2940

[38] Malkin E, Long CA, Stowers AW, et al. Phase 1 Study of Two Merozoite Surface Protein 1 (MSP1(42)) Vaccines for Plasmodium falciparum Malaria. PLoS Clin Trials 2007 Apr;2(4):e12.

[39] Huaman MC, Martin LB, Malkin E, et al. Ex vivo cytokine and memory T cell responses to the 42-k Da fragment of *Plasmodium falciparum* merozoite surface protein-1 in vaccinated volunteeers. *J Immunol*. 2008 Feb 1:180(3):1451-61

[40] Cooper CL, Davis HL, Morris ML, et al. Safety and immunogenicity of CPG 7909 injection as an adjuvant to Fluarix influenza vaccine. Vaccine 2004 Aug 13;22(23-24):3136-43.

[41] Cooper CL, Davis HL, Morris ML, et al. CPG 7909, an immunostimulatory TLR9 agonist oligodeoxynucleotide, as adjuvant to Engerix-B HBV vaccine in healthy adults: a double-blind phase I/II study. J Clin Immunol 2004 Nov;24(6):693-701.

[42] Cooper CL, Davis HL, Angel JB, et al. CPG 7909 adjuvant improves hepatitis B virus vaccine seroprotection in antiretroviral-treated HIV-infected adults. AIDS 2005 Sep 23;19(14):1473-9.

[43] Rynkiewiczi D RMRJea. Marked Enhancement of Antibody Response to Anthrax Vaccine Adsorbed with CPG 7909 in Healthy Volunteers. 45th Interscience Conference on Antimicrobial Agents and Chemotherapy . 2005.
Ref Type: Abstract

[44] Dolo A, Camara F, Poudiougo B, et al. [Epidemiology of malaria in a village of Sudanese savannah area in Mali (Bancoumana). 2. Entomo-parasitological and clinical study]. Bull Soc Pathol Exot 2003 Nov;96(4):308-12.

[45] Toure YT, Doumbo O, Toure A, et al. Gametocyte infectivity by direct mosquito feeds in an area of seasonal malaria transmission: implications for Bancoumana, Mali as a transmission-blocking vaccine site. Am J Trop Med Hyg 1998 Sep;59(3):481-6.

APPENDIX A: MALARIA COMPREHENSION EXAM

(US)

Johns Hopkins University/Bloomberg School of Public Health
Center for Immunization Research

Malaria BSAM-2/Alhydrogel®+CPG 7909 Comprehension Exam

Date: ________________________ Vol. Initials: _________ Screen # _____________

1. As part of this study, you will be injected with a live malaria parasite..... T F
2. This vaccine will protect you from getting malaria .... T F
3. Women enrolled in this study must not be pregnant or nursing............... T F
4. If you change your mind about being in the study after you are vaccinated you can withdraw your consent………………………………T F
5. This vaccine has never been used in humans before and there may be risks we don't know about .... T F
6. You will receive an injection of the same vaccine three times during the study .... T F
7. If you feel sick during the study, you should keep it to yourself .... T F
8. If you join this study, you will need to be followed for a total of 12 months .... T F
9. Before joining the study you will be tested for HIV, hepatitis B, and hepatitis C... T F
10. You will fill out a memory enhancement tool for 6 days after each vaccination.. T F
11. It is OK to enroll in other investigational agent studies while you are still in this study .... T F
12. It is important for you to stay in the clinic for 30 minutes after each injection.. T F
13. You will have blood drawn during most of your clinic visits.................. T F
14. There is no chance that you will develop an autoimmune disease such as lupus from the vaccine .... T F

15. You cannot get malaria from this vaccine study .... T F

16. Are there any risks to you if you participate in this trial? .... Y N
If you answered Yes, name at least 3 risks.

____

____

____

Total # correct before review: ______ Total # correct after review: ________

Reviewed by: ______________________________ Date: ________________

MALARIA COMPREHENSION EXAM

(MALI)

Phase 1 Study of the Safety and Immunogenicity of BSAM-2/Alhydrogel® +CPG 7909, an Asexual Blood Stage Vaccine for *Plasmodium falciparum* Malaria in adults in the US and in Bancoumana, Mali

---

____________________ Census ID #

______________________________ Name (first, last)

1. As part of the study, you will be injected with a live malaria parasite....…………..T F
2. There is a chance you could get sick from this vaccine…………….……………..T F
3. Women enrolled in this study should not become pregnant up until 3 months after the last shot….……………………………………………………….….….…...T F
4. If you change your mind about being in the study after you are vaccinated, you can withdraw your consent……..……………………………….……………..…T F
5. This vaccine has only been given to a small number of people before and there may be risks we don't know about…………………………...……………………………….….…T F
6. You will have your blood drawn as part of this study…………..……………….….…T F
7. There is no chance that you will develop an autoimmune disease such as lupus from the vaccine……………………………………………………………..…… T F
8. You will get 3 vaccinations in this study…………………………………….….…T F
9. If you feel sick during the study, you shouldn't tell anyone…………………....….T F
10. If you join the study, you need to be followed in our clinic for 12 months…........…T F
11. Everybody in this study will get the same kind of vaccine……………….….….…T F
12. Are there any risks to you if you participate in this trial? ………………………… Y N
    If you answered Yes, name it/them.
    ____
    ____
    ____

- Total number correct before review…………………..……… ___
- Total number correct after review…………………………….. ___

Reviewed by ______________________________ Date ___/___/___

Volunteer signature or fingerprint _________________________ Date ___/___/___

Witness signature __________________________ Date ___/___/___

APPENDIX B: SCHEDULE OF PROCEDURES/EVALUATIONS

		Month		0						1	2						3
**Procedures**	**Blood Volume[1]**	**Day**	Pre	0	1	2	3	7	14	28	56[4]	57	58	59	63	70	84
Complete History/Physical			X														
Obtain Informed Consent			X														
Interim Clinical Evaluation				X	X	X	X	X	X	X	X	X	X	X	X	X	X
CBC[2, 3]	5 mL		X	X			X	X	X		X			X	X	X	
ALT/Creatinine	5 mL		X	X			X	X[7]	X		X			X	X[7]	X	
Urine dipstick test			X	X			X[7]	X	X	X	X			X[7]	X	X	X
Urine pregnancy test (females)			X	X					X		X					X	
Urine for chloroquine and metabolites (Mali)				X							X						
HIV (US)	5 mL		X														
HbsAg & HCV ELISA (US)	5 mL		X														
Anti-dsDNA			X													X	
Hepatitis B and C dipstick test (Mali)			X														
Baseline stored serum[3]	5 mL			X													
**VACCINATION**				**X**							**X**						
Anti-AMA1/MSP1 antibody ELISA	5 mL			X					X		X					X	
B cell analysis	20 mL			X							X				X		
GIA and/or T cell studies[5]	20 mL			X											X	X	
Malaria smears and filter paper collection (Mali)			X	X							X					X	
**Blood Volume in mL Mali (US)[6]**			15 (20)	60			10	10	15		35 (40)			10	50	40	
**Total Blood Volume in mL Mali (US)**			15 (20)	75 (80)			85 (90)	95 (95)	110 (110)		145 (150)			155 (150)	205 (195)	245 (235)	

[1]Total blood volume to drawn over course of study is 455 mL in Mali and 460 mL in the US.
[2]CBC parameters to be assessed for safety: WBC, absolute neutrophil count, hemoglobin, and platelet count.
[3]If subsequent to vaccination the subject develops clinical signs of autoimmune or other disease, this sample will be used for baseline laboratory tests as indicated. In Mali the serum saved will be that remaining after ALT/Creatinine is performed and will not require additional blood to be drawn.
[4]Days of second and third vaccinations can range from Study Day 56-65, in which case subsequent follow-up should be adjusted accordingly (i.e., Day 1 after second vaccine instead of Day 29, etc.)
[5]Plasma remaining from T cell studies will be used for GIA. Serum from days when GIA is not scheduled will be reserved and may be used for GIA if additional data points are needed.
[6]In Mali, serum remaining from ALT/Creatinine at screening will also be used for anti-dsDNA. The totals shown here and at Study Day 70 reflect this.

APPENDIX B SCHEDULE OF PROCEDURES/EVALUATIONS (continued)

		Month	6 (4)						7 (5)	8 (6)[7]	9 (7)	10 (8)[7]	11(9)[7]	12	24[8]
		**Day (US)**	180[4]			183	187	194	210		270			360	720
**Procedures**	**Blood Volume[1]**	**Day (Mali)**	120[4]	121	122	123	127	134	150	180	210	240	270	360	720
Complete History/Physical															
Obtain Informed Consent															
Interim Clinical Evaluation			X	X	X	X	X	X	X	X	X	X	X	X	X
CBC[2, 3]	5 mL		X			X	X	X							
ALT/Creatinine	5 mL		X			X	X[7]	X							
Urine dipstick			X			X[7]	X	X	X		X			X	
Urine pregnancy test (females)			X					X							
Urine for chloroquine and metabolites (Mali)			X												
HIV (US)	5 mL														
HbsAg & HCV ELISA (US)	5 mL														
Anti-dsDNA								X						X	
Hepatitis B and C dipstick (Mali)															
Baseline stored serum[3]	5 mL														
**VACCINATION**			X												
Anti-AMA1/MSP1 antibody ELISA	5 mL		X					X			X			X	
B cell analysis	20 mL		X				X							X	
GIA and/or T cell studies[5]	20 mL						X	X						X	
Malaria smears and filter paper collection (Mali)			X					X	X	X	X	X	X	X	
**Blood Volume in mL Mali (US)[6]**			35 (40)			10	50	40			5			50	
**Total Blood Volume in mL Mali (US)**			280 (275)			290 (285)	340 (330)	380 (370)			385 (375)			435 (425)	

[7] These procedures/ visits will occur in Mali only.

[8] This visit is optional in the US

APPENDIX C: TOXICITY TABLE

**Local Adverse Event**	**Grade**	**Intensity**
Pain at injection site	1	Pain that is easily tolerated
	2	Pain that interferes with daily activity or treatment given
	3	Pain that prevents daily activity
Erythema at injection site	1	>0 mm - ≤20 mm
	2	>20 mm - ≤50 mm
	3	>50 mm
Swelling at injection site	1	>0 mm - ≤20 mm
	2	>20 mm - ≤50 mm
	3	>50 mm
Induration at injection site	1	>0 mm - ≤20 mm
	2	>20 mm - ≤50 mm
	3	>50 mm
**Solicited Adverse Event**	**Grade**	**Intensity**
Fever (oral)	1	100.4°F – 101.5°F (38.0°C – 38.6°C)
	2	101.6°F – 102.4°F (38.7°C – 39.1°C)
	3	≥102.5°F (≥39.2°C)
Headache	1	Headache that is easily tolerated
	2	Headache that interferes with daily activity, or treatment given
	3	Headache that prevents daily activity
Nausea	1	Nausea that is easily tolerated
	2	Nausea that interferes with daily activity, or treatment given
	3	Nausea that prevents daily activity
Malaise	1	Malaise that is easily tolerated
	2	Malaise that interferes with daily activity, or treatment given
	3	Malaise that prevents daily activity
Myalgia	1	Myalgia that is easily tolerated
	2	Myalgia that interferes with daily activity, or treatment given
	3	Myalgia that prevents daily activity
Arthralgia	1	Arthralgia that is easily tolerated
	2	Arthralgia that interferes with daily activity, or treatment given
	3	Arthralgia that prevents daily activity
Urticaria	1	Requiring no medications
	2	Requiring PO or topical treatment or IV medications or steroids for ≤24 hours
	3	Requiring IV medication or steroids for >24 hours

APPENDIX D: TOXICITY TABLE FOR GRADING LABORATORY ADVERSE EVENTS

US

Laboratory Test	Grade 1	Grade 2	Grade 3	Grade 4 (Life threatening)
Hgb (female) – decrease from testing laboratory LLN in gm/dl	1.0 - <1.5	≥1.5 & <2.0	≥2.0	Requires transfusion
Hgb (male) – decrease from testing laboratory LLN in gm/dl	≥1.5 & <2.0	≥2.0 & <2.5	≥2.5	Requires transfusion
Absolute neutrophil count (ANC, cells/mm^3)	1000-1499	500-999	<500	<500 with fever
Leukopenia (WBC, cells/mm^3)	<3500 - ≥2500	<2500 - ≥1500	<1500	<1500 with fever
Platelets (cells/mm^3)	125,000 – 135,000	100,000 – 124,000	20,000-99,000	<20,000
ALT	1.25 – 2.5 x ULN	2.6 – 5.0 x ULN	>5.0 x ULN	>10 x ULN and requires hospitalization
Creatinine	1.1 – 1.5 x ULN	1.6 – 3.0 x ULN	>3.0 x ULN	>5.0 x ULN and requires dialysis
Urine protein	2+ or 0.5-1 gm loss/day	3+ or 1-2 gm loss/day	4+ or >2 gm loss/day	NA
Hematuria	2+ confirmed by 5-10 rbc/hpf	3+ confirmed by >10 rbc/hpf	gross, with or without clots, OR red blood cell casts	NA

Mali

Laboratory Test	Grade 1 (Mild)	Grade 2 (Moderate)	Grade 3 (Severe)	Grade 4 (Life threatening)
Hemoglobin<br>Males<br>Females	 8.5 – 9.5 g/dL<br>7.0 – 8.0 g/dL	 7.5 – 8.4 g/dL<br>6.0 – 6.9 g/dL	 <7.5 g/dL<br><6.0 g/dL	Requires transfusion
Platelets	75,000 - 99,999/mm^3	50,000 - 74,999/mm^3	20,000 – 49,999/mm^3	<20,000/ mm^3
WBCs (Increase)	≥11,500 – 15,000/ mm^3	>15,000 - 20,000 /mm^3	>20,000/mm^3	NA
WBCs (Decrease)	<3500-2500/ mm^3	<2500-1000/ mm^3	<1,000 / mm^3	<1,000/ mm^3 with fever
Absolute Neutrophil Count	1000 -1500/mm^3	500-999/mm^3	<500/mm^3	<500/mm^3 with fever
Absolute Granulocyte Count				
Creatinine	1.1 - 1.5 x ULN	1.6 - 3.0 x ULN	>3.0 x ULN	>5.0 x ULN and requires dialysis
ALT	1.25 - 2.5 x ULN	2.6 - 5 x ULN	>5 x ULN	>10 x ULN and requires hospitalization
Proteinuria	2+ or 500 mg - 1 gm loss/day	3+ or >1- 2 gm loss/day	4+ or >2 gm loss/day	NA
Hematuria	2+ confirmed by 5-10 rbc/hpf	3+ confirmed by >10 rbc/hpf	gross, with or without clots, OR red blood cell casts	NA

ULN= upper limits of normal

APPENDIX E: TABLE FOR GRADING ADVERSE EVENTS

These tables are to be used to grade unexpected adverse events not described in Appendixes C and D.

**Vital Signs***	**Mild (Grade 1)**	**Moderate (Grade 2)**	**Severe (Grade 3)**	**Life threatening (Grade 4)**
Tachycardia – beats per minute	110-120	120-140	>140	>160 with symptoms**
Bradycardia – beats per minute	50-54	45-49	<45	<35 with loss of consciousness
Hypertension (systolic) – mm Hg (with repeat testing at same visit)	141-150	151-170	>170	>200 with symptoms**
Hypertension (diastolic) – mm Hg (with repeat testing at same visit)	91-100	100-110	>110	>120 with symptoms**
Hypotension (systolic) – mm Hg (with repeat testing at same visit)	85-89 (and symptomatic)	80-84 (and symptomatic)	<80	<70 with loss of consciousness
* Subject should be at rest for measurement of vital signs<br>** Severe headache, chest pain, altered consciousness, or other signs of acute organ damage				

**Systemic**	**Mild (Grade 1)**	**Moderate (Grade 2)**	**Severe (Grade 3)**	**Life Threatening (Grade 4)**
Anorexia	Loss of appetite without decreased oral intake lasting greater than 48 hours	Loss of appetite associated with decreased oral intake without significant weight loss	Loss of appetite associated with significant weight loss	NA
Vomiting	1-2 episodes/24 hours	>2 episodes/24 hours	Prevents daily activity, requires outpatient IV hydration	Requires hospitalization
Diarrhea	2-3 loose stools/24 hours	4-5 loose stools/24 hours	>6 loose stools or requires outpatient IV hydration	Requires hospitalization
Constipation	NA	Persistent constipation requiring regular use of dietary modifications, laxatives, or enemas	Obstipation with manual evacuation indicated	NA
Fatigue	No interference w/activity	Some interference w/activity	Significant, prevents daily activity	NA

Arthritis	Mild pain with inflammation, erythema or joint swelling – but not interfering with function	Moderate pain with inflammation, erythema or joint swelling – interfering with function, but not with activities of daily living	Severe pain with inflammation, erythema or joint swelling –and interfering with activities of daily living	NA
Mucocutaneous Reaction/Rash	Erythema; pruritus or localized macular rash	Diffuse, maculo-papular rash, dry desquamation	Vesiculation or moist desquamation or ulceration	Requires hospitalization
Vasovagal episode (associated with a procedure of any kind)	Present without loss of consciousness	Present with transient loss of consciousness	NA	NA
Vertigo	Causes no or minimal interference with usual daily activities	Causes greater than minimal interference with usual daily activities	Inability to perform daily activities	NA
Cough	transient- no treatment	persistent cough; treatment responsive	Paroxysmal cough; uncontrolled with treatment	Requires hospitalization
Bronchospasm, Acute	transient; no treatment; 70% - 80% FEV_1 of peak flow	requires treatment; normalizes with bronchodilator; FEV_1 50% - 70% (of peak flow)	no normalization with bronchodilator; FEV_1 25% - 50% of peak flow; or retractions present	Requires hospitalization
Dyspnea	Dyspnea on exertion	Dyspnea with normal activity	Dyspnea at rest	Requires hospitalization
